# Five Tetramic Acid Derivatives Isolated from the Iranian Fungus *Colpoma* *quercinum* CCTU A372

**DOI:** 10.3390/biom11060783

**Published:** 2021-05-22

**Authors:** Gian Primahana, Abolfazl Narmani, Frank Surup, Rémy Bertrand Teponno, Mahdi Arzanlou, Marc Stadler

**Affiliations:** 1 Department Microbial Drugs, Helmholtz Centre for Infection Research GmbH (HZI), Inhoffenstrasse 7, 38124 Braunschweig, Germany; Gian.Primahana@helmholtz-hzi.de (G.P.); Abolfazl.Narmani2@gmail.com (A.N.); Frank.Surup@helmholtz-hzi.de (F.S.); remyteponno@gmail.com (R.B.T.); 2 Research Center for Chemistry, Indonesian Institute of Sciences (LIPI), Kawasan Puspiptek, Serpong, 15314 Tangerang Selatan, Indonesia; 3 Department of Plant Protection, Faculty of Agriculture, University of Tabriz, Tabriz 51666, Iran; Arzanlou@hotmail.com; 4 Department of Chemistry, Faculty of Science, University of Dschang, P.O. Box 67, Dschang, Cameroon

**Keywords:** colposetin A–C, colpomenoic acid A and B, tetramic acid derivatives

## Abstract

Submerged mycelial cultures of the ascomycete *Colpoma* *quercinum* CCTU A372 were found to produce five previously undescribed tetramic acids, for which we propose the trivial names colposetins A–C (**1**–**3**) and colpomenoic acids A and B (**4** and **5**), along with the known compounds penicillide (**6**) and monodictyphenone (**7**). The planar structures of **1**–**5** were determined by high-resolution electrospray ionization mass spectrometry (HR-ESIMS) and extensive 1D and 2D nuclear magnetic resonance (NMR) spectroscopy. Their absolute configurations were determined by the combination of electronic circular dischroism (ECD) analysis, *J*-based configurational analysis, and a rotating-frame Overhauser effect spectroscopy (ROESY) experiment. Colposetin B displayed weak antimicrobial activity against *Bacillus subtilis* and *Mucor hiemalis* (MIC 67 µg/mL).

## 1. Introduction

Fungi are a valuable group of organisms with promising biotechnological and industrial applications. One of them is a producer of secondary metabolites with various biological activities ranging from medicines to agrochemicals [1]. In the last two decades, the discovery rate of novel fungal secondary metabolites has accelerated significantly [2]. Mostly, they were isolated from fungi from underexplored genera, from interesting ecologies, that interact with other fungi, plants, or animals, and that exist in geographic areas that so far have not been exhaustively explored [3].

In the last several years, numerous secondary metabolites have been isolated from underexplored genera or habitats. For example, a novel isoindolinone and indanone, chaetosisoindolinone and chaetosindanone were isolated from a new species of plant associated fungus *Chaetosphaeronema achilleae* collected in Iran [4], udagawanones A and B, two new α-pyrone derivatives isolated from the endophyte *Neurospora udagawae* [5], 3′-malonyl-daldinin F and pseudofuscochalasin A isolated from *Hypoxylon fuscum* complex [6], seven novel xanthone-anthraquinone heterodimers isolated from *Jugulospora vestita* (Sordariomycetes, Ascomycota) [7], pseudopalawanone from *Pseudopalawania siamensis* gen. et sp. nov., collected in northern Thailand [8], and five new diketopiperazines from *Batnamyces globulariicola* gen. et sp. nov. (Chaetomiaceae), a fungus associated with roots of the medicinal plant *Globularia alypum* in Algeria [9].

During our survey on endophytic fungi in August 2016, in the Kaleybar region in northwestern Iran, apparently healthy and symptomless shoots were collected from Willow (*Salix* sp.), from which an isolate of the fungus *Colpoma quercinum* CCTU A372 was obtained and identified through morphological and molecular phylogenetic methods. *Colpoma* is a member of the Rhytismataceae (class Leotiomycetes; order Rhytismatales) and comprises ca. 14 species of plant-associated inoperculate discomycetes with erumpent apothecia [10]. These species are considered saprotrophs or weakly parasitic. The current study is dedicated to the description of the production, isolation, and characterization of the secondary metabolites of this Iranian fungus.

## 2. Materials and Methods

### 2.1. General Experiment

An Agilent 1100 HPLC system (Agilent Technologies, Santa Clara, CA, USA) was used for analytical reversed-phase (RP) measurement and fractionation. Column: XBridge C18 100 × 2.1 mm, 3.5 µm (Waters, Milford, MA, USA); solvent A (H_2_O/ACN, 95:5) and solvent B (H_2_O/ACN, 5:95) supplemented with 5 mM ammonium acetate buffer (5 mmol NH_4_OAc + 0.04 mL/L CH_3_COOH); flowrate 0.3 mL/min using gradient elution system starting from 10% to 100% B in 30 min, hold for 10 min at 100% B and followed by postrun for equilibrating to the initial condition for 10 min; column temperature was set to 40 °C; 96-well microtiter plates were used for fractionation; and the eluates were collected every 30 s. HR-ESIMS (high-resolution electrospray ionization mass spectrometry) data were recorded on a MaXis ESI-TOF (electrospray ionization-time of flight) mass spectrometer (Bruker Daltonics, Bremen, Germany) coupled to an Agilent 1260 series HPLC-UV system and equipped with C18 Acquity UPLC BEH (ultraperformance liquid chromatography) (ethylene bridged hybrid) (Waters) column; DAD-UV detection at 200–600 nm; solvent A (H_2_O) and solvent B (ACN) supplemented with 0.1% formic acid as a modifier; flowrate 0.6 mL/min, 40 °C, gradient elution system with the initial condition 5% B for 0.5 min, increasing to 100% B in 19.5 min and holding at 100% B for 5 min. HPLC-DAD/MS measurements were performed using an amaZon speed ETD (electron transfer dissociation) ion trap mass spectrometer (Bruker Daltonics) and measured in positive and negative ion modes simultaneously. HPLC system (column C18 Acquity UPLC BEH (Waters), solvent A: H_2_O; solvent B: acetonitrile (ACN) supplemented with 0.1% formic acid, gradient conditions: 5% B for 0.5 min, increasing to 100% B in 20 min, maintaining isocratic conditions at 100% B for 10 min, flow rate 0.6 mL/min, UV/Vis detection 200−600 nm). Bruker Compass DataAnalysis 4.4 SR1 was used to analyze the data, including determining the molecular formula using the Smart Formula algorithm (Bruker Daltonics). Purification was performed with a preparative HPLC system on an Agilent 1100 series (Santa Clara, CA, USA) equipped with a 180-fraction collector, binary pump, and diode-array UV detector. ChemStation software Rev. B.04.03.SP1 was used as the system controller. NMR spectra were collected on a Bruker 700 MHz Avance III spectrometer equipped with a 5 mm TCI cryoprobe (^1^H: 700 MHz, ^13^C: 175 MHz), locked to the respective deuterium signal of the solvent. Chemical shifts are given in parts per million [ppm] and coupling constants in Hertz [Hz]. Optical rotations were measured using Anton Paar MCP-150 Polarimeter (Graz, Austria) with 100 mm path length and sodium D line at 589 nm. The UV spectra were measured on a Shimadzu (Kyoto, Japan) UV/Vis 2450 spectrophotometer using methanol (Uvasol, Merck, Darmstadt, Germany) as a solvent. ECD (electronic circular dichroism) spectra were measured using a J-815 spectropolarimeter (Jasco, Pfungstadt, Germany).

### 2.2. Fungal Material and Morphological Analysis

During our routine screening program for novel anti-infective from endophytic fungi, in August 2016, apparently healthy and symptomless 2–3 year old shoots were collected from willow (*Salix* sp.) in Kaleybar region in northwestern Iran, and fungal endophytes were obtained according to previously described protocols [11]. In brief, 10–20 cm length shoots were surface-sterilized for 45–60 s in 70% ethanol, then 10 min in 3% sodium hypochloride (NaClO), and 30 s in 70% ethanol. Samples then dried on sterile filter paper under the clean bench. In total, 4–5 pieces from each sample were cut and transferred on malt extract agar (MEA) plate supplemented with 100 mg/L streptomycin sulphate and 100 mg/L ampicillin. Purification was conducted by hyphal tip technique. Pure cultures were preserved on MEA in 2 mL microtube slants at 4 °C in the University of Tabriz Culture Collection (CCTU). Cultural characteristics of *C. quercinum* CCTU A372 including colony characters and pigment production, shape, and growth rate and microscopic characteristics were recorded on PDA (potato dextrose agar), MEA, and OA (oatmeal agar).

### 2.3. Molecular Analysis, Sequencing, and Phylogenetic Analysis

Genomic DNA was extracted from fresh mycelium of culture grown on MEA. Fungal mycelia were harvested and subjected to DNA extraction using the EZ-10 Spin Column Genomic DNA Miniprep kit (Bio Basic Canada Inc., Markham, ON, Canada) following the manufacturer’s protocol. Molecular analysis was carried out using sequence data of partial large nuclear ribosomal RNA subunit (nrLSU) region, and internal transcribed spacer (ITS) regions based on LROR/LR5 [12] and ITS1/ITS4 [13] primer pairs, respectively. The amplicons were sequenced in both directions using the same primer set. Sequence files were edited using SeqMan software in the Lasergene package (DNASTAR Inc., Madison, WI, USA), and consensus sequence was computed using the forward and reverse sequences. The consensus sequences were compared with sequences in the GenBank using the Basic local alignment search tool (BLAST). Sequences were deposited in GenBank with the accession numbers MZ220485 and MZ203827 for LSU and ITS, respectively. The dataset of ITS-rDNA and LSU gene sequences data for references strains sequences were downloaded from GenBank and included in alignment files. Bayesian analyses were accomplished according to previously published procedure [14].

### 2.4. Scale-up Production, Extraction, and Isolation

Seed cultures were prepared by inoculating five plugs of 14 days well-grown culture in YMG agar (containing malt extract 10 g, glucose 4 g, yeast extract 4 g, and 40 g agar in 1 L tap water and pH adjusted to 6.3 before sterilization) in 500 mL flask filled with 200 mL of medium and incubated for 10 days. After 10 days, 5 mL of the seed cultures were used to inoculate seven liter medium (two batches) with the same composition of the agar medium. After five days, the consumption of glucose was monitored regularly (using Medi-Test, Macherey Nagel, Düren, Germany), and the fermentation was stopped at day 13.

Mycelia and supernatant were separated by vacuum filtration. The mycelial biomass (c.a 327 g) was extracted with 500 mL ethyl acetate (3 times, *w*/*v* ratio 1:2) for 30 min (temperature 40 °C, 10% power intensity) under an ultrasonic bath (Sonorex Digital 10 P, Bandelin Electronic GmH&Co.KG, Berlin, Germany). The combined ethyl acetate was dried under vacuum to provide 670 mg of mycelial crude extract and then subjected to flash chromatography (Grace Reveleris^®^, Columbia, MD, USA) (silica cartridge 24 g, solvent A: DCM, solvent B: acetone, solvent C: ([DCM/acetone 8:2]:MeOH), gradient: 100% A for 5 min, increasing to 37% B in 12 min, increasing to 100% B in 5 min followed by increasing to 100% solvent mixture C in 7 min and holding at 100% solvent C in 5 min). Eleven fractions were collected, even though fractions 1–4 were found to contain almost exclusively fatty acids and discharged. Fraction 5 (21 mg) was further purified by preparative reversed-phase (RP) HPLC (Gemini, 10 µm column, 250 × 21.2 mm (Phenomenex, Torrance, CA, USA), solvent A: water + 0.1% HCOOH), solvent B: acetonitrile + 0.1% HCOOH), flow rate 20 mL/min and UV detection at 210, 240, and 300 nm, gradient: 40% B isocratic for 2 min, from 40% to 57% B in 5 min, and 57% B isocratic for 43 min) to afford penicillide (**6**) (0.8 mg, *t*_R_ = 11.6 min) and monodictyphenone (**7**) (1.1 mg, *t*_R_ = 27.3 min). Fractions 7–10 were combined (53 mg) due to its similar chromatogram profile and purified using the same column and solvent with isocratic conditions 67% B for 60 min to afford compound **1** (4.8 mg, *t*_R_ = 19.3 min) (see Figure S3 in SI for mycelial crude extract).

To the supernatant, 3% (*v*/*v*) of amberlite XAD-16 resin (Rohm and Haas, Frankfurt, Germany) was added and stirred for 2 h. The XAD resin was collected by sieving and washed with distilled water and then extracted in a glass column (5 × 40 cm) with acetone (three portions of 500 mL each, flow rate 15 mL/min). The combined acetone was evaporated to the water phase (250 mL) and extracted with an equal amount of ethyl acetate three times. The ethyl acetate layers were combined, dried over anhydrous sodium sulfate, and evaporated. The lipophilic component was eliminated by partitioned over MeOH/n-heptane to afford 280 mg crude extract. The crude extract was subsequently purified using PLC 2250 (Gilson, WI, USA) over RP-HPLC (Gemini, 10 µm column, 250 × 50 mm, solvent A: water + 0.1% HCOOH, solvent B: acetonitrile + 0.1% HCOOH), flow rate 20–40 mL/min and UV detection at 210, 240, and 300 nm, gradient: starting from 30% B with flowrate 20 mL/min for 2 min increasing to 40% B in 8 min with flowrate 40 mL/min followed by increasing to 70% B in 70 min and finally in 20 min increasing from 70% to 100% B and holding at 100% B for another 10 min to afford compound **2** (1.8 mg, *t*_R_ = 94 min), compound **3** (2.8 mg, *t*_R_ = 87 min), compound **4** (2.1 mg, *t*_R_ = 70 min), and compound **5** (1.6 mg, *t*_R_ = 67 min)(see Figure S4 in SI for supernatant crude extract).

Colposetin A (**1**) white solid; ${\left[\alpha \right]}_{D}^{20}$ −444 (c 0.1, MeOH); CD (MeOH): λ_max_ (Δɛ) 236 (−2.7), 284 (−1.6); UV/Vis (MeOH) λ_max_ (log ε) 266 (3.62), 294 (3.71) nm; NMR data (^1^H: 700 MHz, ^13^C NMR 176 MHz, CD_3_OD) see Table 1; HR-ESIMS: [M+H]^+^ *m/z* 476.2651, calcd. 476.2643 for C_26_H_38_NO_7_, [M+Na]^+^ *m/z* 498.2462, calcd. 498.2473 for C_26_H_37_NaNO_7_, [2M+Na]^+^ *m/z* 973.5036, calcd. 973.5033 for C_52_H_74_N_2_NaO_1_4, *t_R_* = 13.32 min.

Colposetin B (**2**) white solid; ${\left[\alpha \right]}_{D}^{20}$ −375 (c 0.1, MeOH); CD (MeOH): λ_max_ (Δɛ) 236 (−2.3), 284 (−1.6); UV/Vis (MeOH) λ_max_ (log ε) 247 (3.85), 291 (3.91) nm; NMR data (^1^H: 700 MHz, ^13^C NMR 176 MHz, CD_3_OD) see Table 1 [M+H]^+^ *m/z* 418.2592, calcd. 418.2588 for C_24_H_36_NO_5_, [M+Na]^+^ *m/z* 440.2410, calcd. 440.2407 for C_24_H_35_NaNO_5_, [2M+H]^+^ *m/z* 835.5110, calcd. 807.5102 for C_48_H_71_N_2_O_10_, [2M+Na]+ *m/z* 857.4928, calcd. 857.4923 for C_48_H_70_NaN_2_O_10_, *t_R_* = 13.40 min.

Colposetin C (**3**) yellowish solid; ${\left[\alpha \right]}_{D}^{20}$ −62 (c 0.1, MeOH); CD (MeOH): λ_max_ (Δɛ) 236 (−2.3); UV/Vis (MeOH) λ_max_ (log ε) 279 (3.19) nm; NMR data (^1^H: 700 MHz, ^13^C NMR 176 MHz, CD_3_OD) see Table 1; [M+H]^+^ *m/z* 307.1900, calcd. 307.1904 for C_18_H_27_O_4_, [M+Na]^+^ *m/z* 329.1720, calcd. 329.1720 for C_18_H_26_NaO_4_, [2M+H]^+^ *m/z* 613.3733, calcd. 613.3733 for C_36_H_53_O_8_, [2M+Na]+ *m/z* 635.3551, calcd. 635.3551 for C_36_H_52_NO_8_, *t_R_* = 11.85 min.

Colpomenoic acid A (**4**) yellowish solid; ${\left[\alpha \right]}_{D}^{20}$ −25 (c 0.1, MeOH); UV/Vis (MeOH) λ_max_ (log ε) 235 (4.57), 295 (3.92) nm; NMR data (^1^H: 700 MHz, ^13^C NMR 176 MHz, CD_3_OD) see Table 1; [M+H]^+^ *m/z* 434.2537, calcd. 434.2537 for C_24_H_36_NO_6_, [M+Na]^+^ *m/z* 456.2356, calcd. 456.2357 for C_24_H_35_NaNO_6_, [2M+H]^+^ *m/z* 835.5110, calcd. 807.5102 for C_48_H_71_N_2_O_10_, *t_R_* = 12.02 min.

Colpomenoic acid B (**5**) yellowish solid; ${\left[\alpha \right]}_{D}^{20}$ −27 (c 0.1, MeOH); UV/Vis (MeOH) λ_max_ (log ε) 229 (4.41), 286 (3.97) nm; NMR data (^1^H: 700 MHz, ^13^C NMR 176 MHz, CD_3_OD) see Table 1; [M+H]^+^ *m/z* 432.2378, calcd. 432.2381 for C_24_H_34_NO_6_, [M+Na]^+^ *m/z* 454.2197, calcd. 454.2200 for C_24_H_33_NaNO_6_, *t_R_* = 11.66 min.

### 2.5. Antimicrobial Activity Assay

Antimicrobial activity was determined by our established protocol, according to Becker et al. [15]. In general, all of the pure compounds were dissolved in MeOH with a concentration of 1 mg/mL. Assessment of the antimicrobial activity was determined using the serial dilution assay method to calculate the minimum inhibitory concentration (MIC). In this method, the compounds were diluted in a range between 67 and 0.5 µg/mL in 96 well-plate and incubated with the test organism overnight. The next day, growth of the test organisms was visually observed, and MIC was determined in the lowest concentration where a clear zone (no growth of test organism) was observed. In our attempt for novel anti-infective compounds, an established standard protocol using various organisms representing a broad spectrum of pathogens of clinical interest, as well as sensitive indicator strains, was used (bacteria: *Bacillus subtilis* DSM 10, *Staphylococcus aureus* DSM 346, *Acinetobacter baumanii* DSM 30008, *Chromobacterium violaceum* DSM 30191, *Escherichia coli* DSM 1116 and *Pseudomonas aeruginosa* PA14; mycobacteria: *Mycobacterium smegmatis* ATCC 700084 and fungi: *Candida albicans* DSM 1665, *Schizosaccharomyces pombe* DSM 70572, *Mucor hiemalis* DSM 2656, *Wickerhamomyces anomala* DSM 6766 and *Rhodotorula glutinis* DSM 10134). Methanol as a solvent to dissolve the pure compounds was used as the negative control. Oxytetracycline, ciprofloxacin, gentamycin, and kanamycin were used as positive controls against bacteria, whereas nystatin was used against fungi. A detailed experimental procedure can be found in the SI.

### 2.6. Cytotoxicity Assay

Cytotoxicity of compounds **1**‒**5** was initially determined against two cancer cell lines (mouse fibroblasts L-929 and human endocervical adenocarcinoma KB-3.1) by using a 5-day MTT assay according to an established procedure [15].

In 96-well flat-bottom microtiter plates, cell lines with serial dilution of the test compounds (final range: 37 to 0.6 × 10^−3^ µg/mL) were incubated for five days. After five days, the cells were dyed using 3-(4,5-dimethyl-2-thiazolyl)-2,5-diphenyl-2H-tetrazolium bromide (MTT), which is only converted to its purple formazan derivative by living cells. The half-maximum inhibitory concentration (IC_50_ in µM) was calculated via the colorimetric method in a microplate reader using absorption at 595 nm. In addition, when an inhibition with an IC_50_ < 50 µM was observed, other cell lines were subjected to the test compounds to evaluate their further cytotoxicity: PC-3 (human prostate adenocarcinoma), SK-OV-3 (human ovary adenocarcinoma), MCF-7 (human breast adenocarcinoma), A-431 (human squamous carcinoma), and A549 (human lung carcinoma). A detailed procedure, including the colorimetric method to determine the cell viability, can be found in the SI.

## 3. Results and Discussion

**Figure 1:**
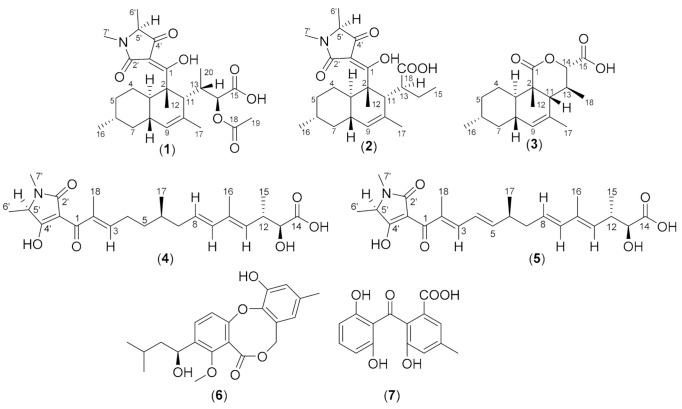
Structure of isolated compounds **1**–**7**.

Purification of the crude extract from *C. quercinum* CCTU A372 (see morphological analysis and molecular identification, Figure S1, Figure S2, and Table S1 in Supplementary Information (SI)) using Si-Flash and preparative RP (reversed-phase) chromatography techniques led to the isolation of five previously undescribed compounds to which we gave trivial names colposetins A–C (**1**–**3**), colpomenoic acid A and B (**4** and **5**) together with the known metabolites penicillide (**6**) and monodictyphenone (**7**) (Figure 1).

**Figure 2:**
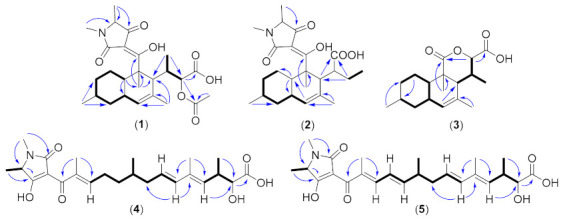
Key correlations of compounds **1**–**5**, selected COSY (bold bond) and HMBC (blue arrows).

Colposetin A (**1**) was isolated as a white solid. The molecular formula of C_26_H_37_NO_7_ was deduced from the HR-ESIMS which showed the molecular ion clusters [M+H]^+^ at *m/z* 476.2651 (calcd for C_26_H_38_NO_7_ 476.2643) and [M+Na]^+^ at *m/z* 498.2462 (calcd for C_26_H_37_NaNO_7_ 498.2473) (Figures S3–S5 in SI), implying nine double-bond equivalents. The ^1^H NMR spectrum of **1** showed signals of an olefinic proton at *δ*_H_ 5.37 (s, H-9), an oxymethine at *δ*_H_ 4.65 (d, *J* = 8.6, H-14), and seven methyls, including one methyl attached to a heteroatom. The ^1^H and ^13^C NMR as well as the HSQC (heteronuclear single-quantum coherence) spectrum revealed the presence of 24 carbons assigned as seven methyl, three methylene, eight methines, and six quaternary carbons, of which three were carbonyls at *δ*_C_ 172.5 (C-18), 174.2 (C-15), and 194.9 ppm (C-4’). The presence of decalin-fused ring system was clearly supported by the HMBC (heteronuclear multiple-bond correlation) cross-peak between the olefinic proton at *δ*_H_ 5.37 (s, H-9), and the carbons at *δ*_C_ 49.8 (C-11), 43.4 (C-7), and 41.3 (C-3) (see Figures S6–S11 in SI). In addition, three methyl groups were attached on the decalin moiety at C-6, C-10, and C-2 based on the HMBC correlations. A fragment of 2-acetoxy-butanoic acid was present in colposetin A as evidenced by the HMBC correlations of CH_3_-20 to C-14 (*δ*_H_/*δ*_C_, 4.65/77.1 ppm), oxymethine H-14 (*δ*_H_ 4.65) to the carboxyl at C-15 (*δ*_C_ 174.3) and C-18 (*δ*_C_ 172.5) as well as CH_3_-19 to C-18. This fragment was attached to C-11, based on the HMBC correlations from Me-20 and the oxymethine proton H-14 to C-11 (*δ*_C_ 49.8) (Figure 2).

Compound **2** also isolated as a white solid showed ion clusters at *m/z* 418.2592 [M+H]^+^ (calcd. for C_24_H_36_NO_5_: 418.2588) and *m/z* 440.2410 [M+Na]^+^ (calcd. for C_24_H_35_NaNO_5_: 440.2407) indicating a molecular formula of C_24_H_35_NO_5_ (accounting for eight double-bond equivalents) (Figures S12–S14 in SI). Interpretation of 1D and 2D NMR data revealed that the structure of **2** was very similar to that of **1**, the difference being only in the side chain at C-11. Instead of 2-acetoxy-butanoic acid in **1**, colposetin B was bearing butyric acid at C-11 position supported by the HMBC correlation from methylene protons at *δ*_H_ 1.86 (m, H-14a), 1.17 (m, H-14b) to C-11 (*δ*_C_ 29.5) of the decalin moiety. Furthermore, an HMBC correlation was observed between the methine proton at *δ*_H_ 2.24 (brs, H-11) and the carboxyl group at *δ*_C_ 177.5 (C-18) (see Figure S15–S20 SI).

The molecular formula of colposetin C (**3**) isolated as a yellowish solid was deduced as C_18_H_26_O_4_ from the HR-ESIMS, which showed molecular ion clusters [M+H]^+^ at *m/z* 307.1900 (calcd. for C_18_H_27_O_4_: 307.1904) and [M+Na]^+^ at *m/z* 329.1720 (calcd. for C_18_H_26_NaO_4_: 329.1720) (see Figures S21–S23 in SI). According to the interpretation of 1D and 2D NMR data, the decalin moiety was also present in **3**, but lacking the tetramic acid moiety. A third ring system named 3-methyl-6-oxotetrahydro-2H-pyran-2-carboxylic acid was connected at C-1 and C-11, deduced from the HMBC correlations from Me-12 to C-1 and C-2 as well as from the oxymethine at *δ*_H_ 4.89 ppm (d, *J* = 1.3, H-14) to C-1 and C-15 (Figures S24–S29 in SI).

**Figure 3:**
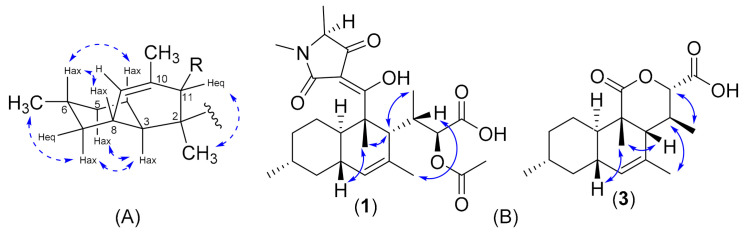
Key ^1^H, ^1^H NOE correlations of decalin moiety of compound **1** (**A**) and ROESY (solid blue arrows) of compound **1** and **3** (**B**).

**Figure 4:**
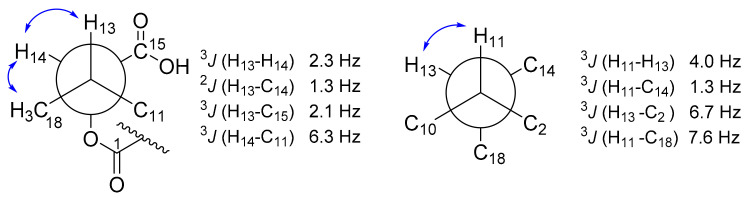
Selected conformation of Newman projection of compound **3** for determining stereocenter at C-13 and C-14 using *J*-based configurational analysis; blue arrow indicated ROESY correlations.

The stereochemistry of compounds **1**–**3** was assigned by a combination of several methods, including CD spectroscopy, ROESY experiment, and *J*-based configurational analysis. The ROESY observations of the decalin moiety of our compounds (Figure 3) were similar to the observed NOESY data of paecilosetin A [16] indicating a (2S*, 3R*, 6R*, 8S*, 11R*) relative configuration. The negative Cotton effect at 234 and 285 nm (see Figures S30 and S31 in SI) is diagnostic for the 2*S*,5′*S* absolute configuration [16,17]. Thus, we concluded the absolute configuration of the decalin moiety as (2S, 3*R*, 6*R*, 8*S*, 11*R*, 5′*S*). *J*-based configurational analysis was chosen to determine the stereocenter at C-13 and C14. However, some of the coupling constants were not observed in the HSQC-Hecade and *J*-HMBC experiments of compound **1**; therefore, the *J*-based configurational method gave equivocal results. To address this problem, we opted to use compound **3** as a model for *J*-based configurational analysis. For a compound with 1,2 adjacent methine system such as in **3**, the relative stereogenic center can be represented by six possible staggered rotamers (see Figures S32 and S33 in SI for all rotamers). The small coupling constant (1.3 Hz) was observed between ^2^*J*(H-13/C-14) indicating an anti-periplanar configuration between H-13 and –OC1 [18]. A small ^3^*J*(H-13/H-14) = 2.3 Hz as well as a small ^3^*J*(H-13/C-15) = 1.7 Hz indicated for gauche-like conformation between H-13/H-14 and H-13/C-15 (Figure 4). Furthermore, a rather large ^3^*J*(H-14/C-11) = 6.3 Hz indicated an anti-periplanar configuration between H-14 and C-11 (see Figures S34 and S35 in SI). Using the same technique, a small ^3^*J*(H-11/H-13) = 4.0 Hz and ^3^*J*(H-11/H-14) = 1.3 Hz also indicated gauche-like conformation between H-11/H-13 and H-11/H-14. An antiperiplanar conformation between H-13/C-2 and H11/C-18 was deduced from the large coupling of ^3^*J*(H-13/C-2) = 6.7 Hz and ^3^*J*(H-11/C-18) = 7.6 Hz, thus completing the stereocenter assignment in the compound family **1**–**3**. Taken together, 13*S* and 14*S* absolute configurations were concluded.

The molecular formula of colpomenoic acid A (**4**) was deduced to be C_24_H_35_NO_6_ from the ion clusters at *m/z* 434.2537 [M+H]^+^ (calcd. for C_24_H_36_NO_6_: 434.2537) and *m/z* 456.2356 [M+Na]^+^ (calcd. for C_24_H_35_NaNO_6_: 456.2357) (Figures S36–S38 SI). The ^1^H NMR of **4** (Table 2) exhibited the signals of four olefinic protons (*δ*_H_ 5.37–6.98), one oxymethine (*δ*_H_ 4.06) and six methyls. Careful analysis of the ^13^C NMR and HSQC spectrum revealed 23 carbons which could be sorted out to eight olefinic carbons, three methylenes, three methines (one oxymethine), and seven quaternary carbons, including three carbonyls. The interpretation of 1D and 2D NMR spectral data showed that the tetramic acid moiety was present in **4**. A fragment connected at C-3′ of the tetramic acid moiety named hydroxy-tetramethyl-oxopentadeca-trienoic acid was confirmed from the interpretation of ^1^H,^1^H COSY (correlation spectroscopy), HSQC, and HMBC data. Four methyl substituents were located at C-12, C-10, C-6, and C-2 according to the HMBC correlations of CH_3_-15 to C-13 and C-11, CH_3_-16 to C-11 and C-9, CH_3_-17 to C-7 and C-5, and CH_3_-18 to C-3 and C-1. The hydroxyl group was placed at C-13 based on the HMBC correlations of the oxymethine proton H-13 (*δ*_H_ 4.06) to C-14 and C-15. The *E* configuration of the Δ^8,9^ double bond was assigned from the vicinal coupling constant of 15.0 Hz depicted between H-8 (δ_H_ 5.60, dt, *J* = 15.0, 10.0 Hz) and H-9 (δ_H_ 6.05, d, *J* = 15.0). Furthermore, the methyl-substituted Δ^2,3^, Δ^10,11^ double bonds were established as *E* from the ^1^H,^1^H ROESY correlations of CH_3_-18 and H_2_-4, CH_3_-16 and H-8 as well as H-9 and H-11 (see Figures S39–S44 in SI).

The molecular formula C_24_H_33_NO_6_ of colpomenoic acid B (**5**) was determined by the HR-ESIMS which exhibited ion clusters at *m/z* 432.2378 [M+H]^+^ (calcd. for C_24_H_34_NO_6_: 432.2381) and 454.2197 [M+Na]^+^ (calcd. for C_24_H_33_NaNO_6_: 454.2200) (Figures S45–S47 SI). The ^1^H NMR and ^13^C NMR data of **5** were very similar to those of **4**, together with mass spectrometry data showing two hydrogen deficiencies indicating one additional double bond in **5**. The new double bond occurred between C-4 and C-5, characterized by the presence of olefinic signals at *δ*_H_/*δ*_C_ 6.53/125.9 (C-4) and *δ*_H_/*δ*_C_ 6.15/151.9 (C-5), which was not present in **4**. The configurations of the double bonds at Δ^2,3^, Δ^8,9^, and Δ^10,11^ in **5** were similar to those in compound **4**, and the additional double bond at Δ^4,5^ was assigned as *E* from the large vicinal coupling constant of 14.5 Hz (see Figures S48–S53 in SI). Since compounds **4** and **5** are produced from the same organism, we suspected that the stereocenter has a similar configuration to **1**–**3**.

**Figure 5:**
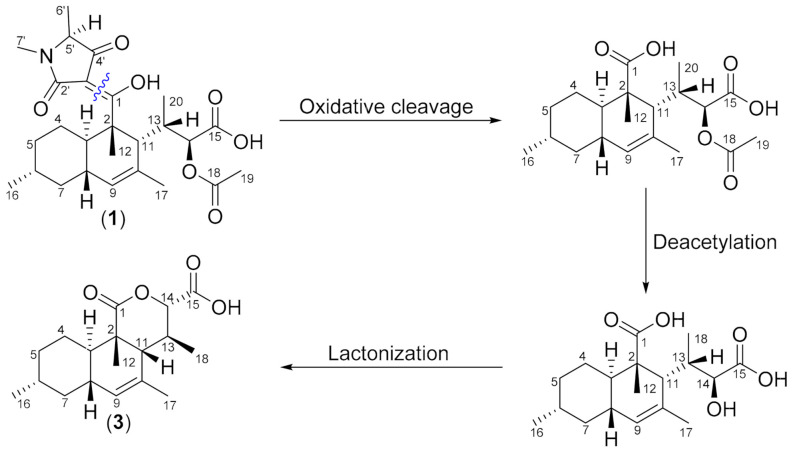
Proposed biogenetic pathway for the formation of compound **3** from **1**.

**Figure 6:**
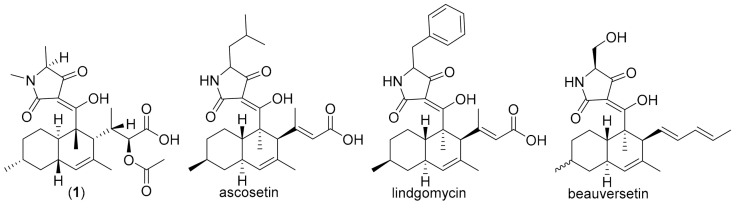
Chemical structures of selected known compounds related to this study.

Natural products containing tetramic acid moieties are common in nature and have been isolated from various sources such as terrestrial or marine bacteria, sponges and fungi [19]. Equisetin from *Fusarium* spp. was the first discovered member of the so-called 3-decalinoyltetramic acid family from fungi [20]. This was followed by other compounds, including altersetin [21], ophiosetin [22], simplicone B [23], and oxaleimide A [24]. Fungal tetramic acids are commonly synthesized from hybrid polyketide synthases (PKS) and non-ribosomal peptide synthetases (NRPS) [25]. It is now known that the decalin ring system in 3-decalinoyltetramic acids results from intramolecular Diels–Alder (IMDA) cycloadditions [24,26]. Although the biosynthetic gene cluster for **1**–**5** in our *Colpoma* strain remains to be identified after generation and analysis of the genome sequence, we hypothesize that compound **1**–**3** are derived from an IMDA reaction of **4** or **5** as precursor via a different biogenetic pathway. Furthermore, compound **3** could derive from metabolite **1** by the oxidative cleavage of the C_1_-C_3′_ double bond followed by deacetylation and lactonization (Figure 5). The core structure of **1**, **2**, and **3** were similar to ascosetin [27], lindgomycin [28], and beauversetin [28] isolated from fungi, with the differences in the substituent at C11 and C5′ (see Figure 6). So far, only one record of endolichenic fungus *Colpoma* sp. CR1565A identified from the Costa Rican plant *Henriettea tuberculosa* (Melatomataceae) was studied for their secondary metabolites and found to produce exclusively compounds that belong to the diketopiperazine family [29]. According to our literature survey, this is the first report of tetramic acid derivatives isolated from this genus.

The isolated compounds (**1**–**5**) were subjected to antimicrobial and cytotoxicity assays (Table 3) using our established screening platform, according to Becker et al. [15]. In our antimicrobial test panel, only colposetin B exhibited weak activity against *Bacillus subtilis* DSM 10 and *Mucor hiemalis* DSM 2656 with MIC 67 µg/mL, respectively. On the other hand, no inhibition was observed to the other test organism until the highest concentration at 67 µg/mL. Regarding cytotoxicity, compounds **1**–**5** were tested using an MTT assay against mouse fibroblast L-929 and human endocervical adenocarcinoma KB-3.1 cell lines in the initial screening. Only colposetin B displayed cytotoxic activity with IC_50_ 57.5 µM (mouse fibroblast L-929) and 5.7 µM (Hela KB-3.1), whereas compound **1**, **3** and **4**–**5** were not active. Furthermore, colposetin B was tested against several different cell lines. Significant cytotoxicity was observed against MCF-7, SK-OV-3, and A-431 with IC_50_ ranging 6.2–8.1 µM (see Table 3). Against A-549 and PC-3 cell lines, colposetin B only showed moderate cytotoxicity with IC_50_ 16.5 and 17.9 µM, respectively (see Figures S56–S62 for the graph calculating IC_50_). It is known that tetramic acid showed broad activity against Gram-positive bacteria and cytotoxicity against several cancer cell lines [30]. Recently, Larson et al. investigated the mode of action of antimicrobial properties of tetramic acid derivatives pyrrolocin C and equisetin against Gram-positive bacteria. They found that the ability of drug (compounds) penetration was responsible for the different activity. They concluded that pyrrolocin C and equisetin inhibit bacterial acetyl-CoA carboxylase in fatty acid biosynthesis by using transcriptomic data, metabolomic analysis, fatty acid rescue, and acetate incorporation experiments [31], but this finding was rather inconclusive and needed further investigation. Regarding its cytotoxicity, the mode of action of the tetramic acid compounds is not well understood and may become an interesting topic for further study.

## 4. Conclusions

The five new secondary metabolites isolated from the endophytic fungus *Colpoma quercinum* CCTU A372 have proven that fungi are a prolific source for novel secondary metabolites, and many are waiting to be discovered. Tetramic acid is known to possess broad bioactivity. For example, macrocidins are known to have herbicidal activity [32] and recently have also been found to have an antibiofilm effect against *Staphylococcus aureus* [33]. Although only weak to moderate antimicrobial activity was observed from our compounds, further investigation of another biological assay remains necessary.

## Figures and Tables

**Table 1:** ^1^H and ^13^C NMR spectroscopic data of compound **1**–**3** in CD_3_OD (^1^H 700 MHz; ^13^C 176 MHz).

Pos.	1		2		3	
	δH, m (*J* in Hz)	δC, Type	δH, m (*J* in Hz)	δC, Type	δH, m (*J* in Hz)	δC, Type
1	-	200.7, C	-	200.1, C	-	177.3, C
2	-	51.7, C	-	49.8, C	-	46.5, C
3	1.91, m	41.3, CH	2.17, m	40.1, CH	1.35, m	44.2, CH
4	1.96; 0.98, m	30.0, CH2	2.11; 1.02, m	30.0, CH2	1.68; 1.21, m	28.5, CH2
5	1.80; 1.08, m	37.6, CH2	1.15, m	37.3, CH2	1.79; 0.86, m	37.2, CH2
6	1.54, m	35.1, CH	1.52, m	35.3, CH	1.45, m	34.0, CH
7	1.83; 0.81, m	43.4, CH2	1.72; 0.96, m	42.6, CH2	1.83; 0.74, m	43.9, CH2
8	1.85, m	41.5, CH	1.80, m	42.4, CH	1.86, m	38.8, CH
9	5.37, s	130.0, CH	5.20, s	128.8, CH	5.39, s	130.5, CH
10	-	132.7, C	-	132.8, C	-	131.5, C
11	3.02, s (br)	49.8, CH	2.24, s (br)	53.0, CH	2.18, d (4.04)	48.4, CH
12	1.41, s	16.0, CH3	1.38, s	14.7, CH3	1.11, s	19.5, CH3
13	2.02, t (7.6)	37.8, CH	2.92, m	50.2, CH	2.53, m	32.2, CH
14	4.65, d (8.6)	77.1, CH	1.86, 1.17, m	29.5, CH2	4.89, d (1.3)	83.0, CH
15	-	174.3, C	0.72, t (7.2)	13.7, CH3	-	174.4, C
16	0.93, d (7.0)	23.1, CH3	0.92, d (7.0)	23.1, CH3	0.91, d (7.0)	22.8, CH3
17	1.78, s	24.4, CH3	1.77, s	15.5, CH3	1.66, s	21.8, CH3
18	-	172.5, C	-	177.5, C	1.18, d (7.0)	15.5, CH3
19	2.08, s	21.3, CH3	-	-	-	-
20	1.01, d (7.7)	17.4, CH3	-	-	-	-
1′	-	-	-	-	-	-
2′	-	n.d	-	n.d	-	-
3′	-	n.d	-	n.d	-	-
4′	-	194.9, C	-	194.5, C	-	-
5′	3.73, s (br)	62.8, CH	3.74, m	62.9, CH	-	-
6′	1.33, d (7.0)	15.4, CH3	1.34, br	15.5, CH3	-	-
7′	2.99, s	27.2, CH3	3.01, s	27.1, CH3	-	-

n.d: not detected.

**Table 2:** ^1^H and ^13^C NMR spectroscopic data of compound **1** and **2** in CD_3_OD (^1^H 700 MHz; ^13^C 176 MHz).

Pos.	4		5	
	δH, m (*J* in Hz)	δC, Type	δH, m (*J* in Hz)	δC, Type
1	-	186.0, C	-	184.4, C
2	-	130.4. C	-	128.6, C
3	6.98, t (7.2)	146.9, CH	7.64, d (9.2)	143.7, CH
4	2.32, m	27.7, CH_2_	6.53, dd, (15.0, 11.0)	125.9, CH
5	1.54; 1.40, m	36.3, CH_2_	6.15, dd (15.0, 7.7)	151.9, CH
6	1.63, m	34.4, CH	2.45, dt (14.0, 7.0 (2x))	39.5, CH
7	2.15; 1.99, m	41.4, CH_2_	2.15; 2.33, m	41.2, CH_2_
8	5.60, dt (15.0, 10.0)	127.6, C	5.58, m	126.8, CH
9	6.05, d (15.0)	137.8, CH	6.07, d (15.0)	138.2, CH
10	-	135.6, C	-	135.6, C
11	5.37, d (10.0)	131.2, CH	5.39, d (10.0)	131.7, CH
12	3.05, ddd (10.0, 7.0, 4.0)	37.9, CH	3.03, m	37.9, CH
13	4.06, d (4.0)	75.7, CH	4.06, d (4.0)	75.7, CH
14	-	177.1, C	-	177.1, C
15	1.09, d (7.0)	18.1, CH_3_	1.09, d (7.0)ov	18.1, CH_3_
16	1.74, d (1.1)	13.1, CH_3_	1.74, d (1.0)	13.1, CH_3_
17	0.93, d (7.0)	20.0, CH_3_	1.08, d (7.0)ov	20.1, CH_3_
18	1.90, s	12.6, CH_3_	1.99, s	12.8, CH_3_
1′	-	-	-	-
2′	-	174.9, C	-	175.2, C
3′	-	n.d	-	n.d
4′	-	195.8, C	-	196.1, C
5′	3.80, q (6.9)	63,1, CH	3.80, q (6.9)	63.3, CH
6′	2.97, s	27.0, CH_3_	2.98, s	27.0, CH_3_
7′	1.34, d (7.0)	15.5, CH_3_	1.35, d (7.0)	15.5, CH_3_

n.d: not detected.

**Table 3:** Antimicrobial and cytotoxicity activities of **1**–**5**.

Microorganism	1	2	3	4	5	Ref
	MIC (µg/mL)					(µg/mL)
*Bacillus subtilis* DSM 10	n.i	67	n.i	n.i	n.i	8.3 ^a^
*Staphylococcus aureus* DSM 346	n.i	n.1	n.i	n.i	n.i	1.7 ^a^
*Mucor hiemalis* DSM 2656	n.i	67	n.i	n.i	n.i	4.2 ^b^
*Pichia anomala* DSM 6766	n.i	n.i	n.i	n.i	n.i	4.2 ^b^
*Rhodotorula glutinis* DSM 10134	n.i	n.i	n.i	n.i	n.i	1.0 ^b^
*Acinetobacter baumanii* DSM 30008	n.i	n.i	n.i	n.i	n.i	0.26 ^c^
*Escherichia coli*	n.i	n.i	n.i	n.i	n.i	1.7 ^d^
**Cell lines**	**IC_50_ (µM)**					**Ref ^e^ (µM)**
mouse fibroblast L-929	n.d	57.5	n.d	n.d	n.d	8.3 × 10^−4^
endocervical adenocarcinoma KB-3.1	n.d	5.7	n.d	n.d	n.d	5.3 × 10^−5^
human breast adenocarcinoma MCF-7	n.t	7.9	n.t	n.t	n.t	7.1 × 10^−5^
human lung carcinoma A-549	n.t	16.5	n.t	n.t	n.t	5.5 × 10^−5^
human prostate cancer PC-3	n.t	17.9	n.t	n.t	n.t	2.7 × 10^−4^
ovarian carcinoma SK-OV-3	n.t	8.1	n.t	n.t	n.t	2.4 × 10^−4^
squamous cell carcinoma A-431	n.t	6.2	n.t	n.t	n.t	8.3 × 10^−5^

n.i: no inhibition; n.d: not detected; n.t: not tested; ^a^: ocytetracycline; ^b^: nystatin; ^c^: ciprofloxacin; ^d^: oxytetracycline; ^e^: epothilone B.

## Data Availability

The data presented in this study are available in the Supplementary Information.

## Supplementary Materials

The following are available online at https://www.mdpi.com/article/10.3390/biom11060783/s1, Figure S1: Morphological characteristics of *Colpoma quercinum* CCTU A372. a-b- 14 day old culture on PDA, MEA and OA, Figure S2: Consensus phylogram (75% majority rule) of 670 trees resulting from a Bayesian, Figure S3: HPLC-UV/Vis chromatogram at 210 nm of the mycelial crude extract of *C. quercinum* CCTU A372, Figure S4: HPLC-UV/Vis chromatogram at 210 nm of the supernatant crude extract of C. *quercinum* CCTU A372, Figure S5: HPLC-DAD/MS chromatogram of colposetin A, Figure S6: HR-ESIMS chromatogram of colposetin A, Figure S7: UV/vis spectrum of colposetin A in MeOH, Figures S8–S13: 1D and 2D NMR spectrum of colposetin A in CD_3_OD, Figure S14: HPLC-DAD/MS chromatogram of colposetin B, Figure S15: HR-ESIMS chromatogram of colposetin B, Figure S16: UV/vis spectrum of colposetin B in MeOH, Figures S17–S22: 1D and 2D NMR spectrum of colposetin B in CD_3_OD, Figure S23: HPLC-DAD/MS chromatogram of colposetin C, Figure S24: HR-ESIMS chromatogram of colposetin C, Figure S25: UV/vis spectrum of colposetin C in MeOH, Figures S26–S31: 1D and 2D NMR spectrum of colposetin C in CD_3_OD, Figure S32: CD spectrum of compounds 1 and 2, Figure S33: CD spectrum of compound 3, Figure S34: *J*-based configurational analysis of six hypothetical rotamers represents 13*S*, 14*S*, Figure S35: *J*-based configurational analysis of six hypothetical rotamers represents 11*R*, 13*S*, Figure S36: ^1^H, ^13^C HSQC-Hecade NMR spectrum of colposetin C in CD_3_OD (700 MHz), Figure S37: ^1^H, ^13^C *J*-HMBC NMR spectrum of colposetin C in CD_3_OD (700 MHz), Figure S38: HPLC-DAD/MS chromatogram of colpomenoic acid A, Figure S39: HR-ESIMS chromatogram of colpomenoic acid A, Figure S40: UV/vis spectrum of colpomenoic acid A in MeOH, Figure S41–S46: 1D and 2D NMR spectrum of colpomenoic acid A in CD_3_OD, Figure S47: HPLC-DAD/MS chromatogram of colpomenoic acid B, Figure S48: HR-ESIMS chromatogram of colpomenoic acid B, Figure S49: UV/vis spectrum of colpomenoic acid B in MeOH, Figures S50–S55: 1D and 2D NMR spectrum of colpomenoic acid B in CD_3_OD, Figure S56: Graph for calculating IC_50_ of colposetin B against mouse fibroblasts L-929, Figure S57: Graph for calculating IC_50_ of colposetin B against human endocervical adenocarcinoma KB-3.1, Figure S58: Graph for calculating IC_50_ of colposetin B against human breast adenocarcinoma MCF-7, Figure S59: Graph for calculating IC_50_ of colposetin B against human lung carcinoma A-549, Figure S60: Graph for calculating IC_50_ of colposetin B against human prostate cancer PC-3, Figure S61: Graph for calculating IC_50_ of colposetin B against ovarian carcinoma SK-OV-3, Figure S62: Graph for calculating IC_50_ of colposetin B against squamous cell carcinoma A-431, Table S1: List of reference taxa and corresponding reference sequences selected for the molecular phylogeny.
