# Supplementary material for: Five Tetramic Acid Derivatives Isolated from the Iranian Fungus Colpoma quercinum CCTU A372
Source: Biomolecules. 2021 May 22;11(6):783. doi: 10.3390/biom11060783 (PMC8224775; doi:10.3390/biom11060783)
Supplement: Supplementary file 1 [file biomolecules-11-00783-s001.zip › biomolecules-1233086-supplementary.pdf]

Supporting Information

Five Tetramic Acid Derivatives Isolated from the Iranian Fungus *Colpoma quercinum* CCTU A372

Gian Primahana [1,2,‡], Abolfazl Narmani [1,3,‡], Frank Surup [1], Rémy Bertrand Teponno [1,4], Mahdi Arzanlou [3] and Marc Stadler [1,*]

[1] Department Microbial Drugs, Helmholtz Centre for Infection Research GmbH (HZI), Inhoffenstrasse 7, 38124 Braunschweig, Germany; Gian.Primahana@helmholtz-hzi.de (G.P.); Frank.Surup@helmholtz-hzi.de (F.S).; Marc.Stadler@helmholtz-hzi.de (M.S.)

[2] Research Center for Chemistry, Indonesian Institute of Sciences (LIPI), Kawasan Puspiptek, Serpong, 15314 Tangerang Selatan, Indonesia

[3] Department of Plant Protection, Faculty of Agriculture, University of Tabriz, Tabriz 51666, Iran; Abolfazl.Narmani2@gmail.com (A.N.); Arzanlou@hotmail.com

[4] Department of Chemistry, Faculty of Science, University of Dschang, P.O. Box 67, Dschang, Cameroon; remyteponno@gmail.com (R.B.T.)

[‡]Author contributed equally

Correspondence: Prof. Dr. Marc Stadler, Department Microbial Drugs, Helmholtz Centre for Infection Research GmbH (HZI), Inhoffenstrasse 7, 38124 Braunschweig, Germany

Email: Marc.Stadler@helmholtz-hzi.de

Table of contents

Morphological analysis and molecular identification 5

Figure S1. Morphological characteristics of *C. quercinum* CCTU A372. A-C: 14 days old culture on PDA (A), MEA (B) and OA (C) (the upper half: colony on the surface; the lower half: colony on the reverse) 6

Table S1. List of reference taxa and corresponding reference sequences selected for the molecular phylogeny. 6

Figure S2. Consensus phylogram (75% majority rule) of 670 trees resulting from a Bayesian........ 7

Figure S3. HPLC-UV/Vis chromatogram at 210 nm of the mycelial crude extract of *C. quercinum* CCTU A372 8

Figure S4. HPLC-UV/Vis chromatogram at 210 nm of the supernatant crude extract of *C. quercinum* CCTU A372 9

Figure S5. HPLC-DAD/MS chromatogram of colposetin A........ 10

Figure S6. HR-ESIMS chromatogram of colposetin A 10

Figure S7. UV/vis spectrum of colposetin A in MeOH........ 10

Figure S8. ^{1}H NMR spectrum of colposetin A in CD_3OD (700 MHz)........ 11

Figure S9. ^{13}C NMR spectrum of colposetin A in CD_3OD (700 MHz) 12

Figure S10. ^{1}H,^{1}H COSY NMR spectrum of colposetin A in CD_3OD (700 MHz)........ 13

Figure S11. ^{1}H,^{13}C HSQC-DEPT NMR spectrum of colposetin A in CD_3OD (700 MHz, 176 MHz) 14

Figure S12. ^{1}H,^{13}C HMBC NMR spectrum of colposetin A in CD_3OD (700 MHz, 176 MHz) 15

Figure S13. NOESY NMR spectrum of colposetin A in CD_3OD (700 MHz) 16

Figure S14. HPLC-DAD/MS chromatogram of colposetin B........ 17

Figure S15. HR-ESIMS chromatogram of colposetin B 17

Figure S16. UV/vis spectrum of colposetin B in MeOH........ 17

Figure S17. ^{1}H NMR spectrum of colposetin B in CD_3OD(700 MHz)........ 18

Figure S18. ^{13}C NMR spectrum of colposetin B in CD_3OD (700 MHz)........ 19

Figure S19. ^{1}H,^{1}H COSY NMR spectrum of colposetin B in CD_3OD (700 MHz)........ 20

Figure S20. ^{1}H,^{13}C HSQC-DEPT NMR spectrum of colposetin B in CD_3OD (700 MHz, 176 MHz) 21

Figure S21. ^{1}H,^{13}C HMBC NMR spectrum of colposetin B in CD_3OD (700 MHz, 176 MHz)........ 22

Figure S22. NOESY NMR spectrum of colposetin B in CD_3OD (700 MHz)........ 23

Figure S23. HPLC-DAD/MS chromatogram of colposetin C........ 24

Figure S24. HR-ESIMS chromatogram of colposetin C 24

Figure S25. UV/vis spectrum of colposetin C in MeOH........ 24

Figure S26. ^{1}H NMR spectrum of colposetin C in CD_3OD (700 MHz)........ 25

Figure S27. ^{13}C NMR spectrum of colposetin C in CD_3OD (700 MHz)........ 26

Figure S28. ^{1}H,^{1}H COSY NMR spectrum of colposetin C in CD_3OD (700 MHz)........ 27

Figure S29. ^{1}H,^{13}C HSQC-DEPT NMR spectrum of colposetin C in CD_3OD (700 MHz, 176 MHz) 28

Figure S30. $^{1}H,^{13}C$ HMBC NMR spectrum of colposetin C in CD_3OD (700 MHz, 176 MHz)........... 29
Figure S31. ROESY NMR spectrum of colposetin C in CD_3OD (700 MHz)..................................... 30
Figure S32. CD spectrum of compounds 1 and 2.. 31
Figure S33. CD spectrum of compound 3. 31
Figure S34. *J*-based configurational analysis of six hypothetical rotamers represents 13*S*, 14*S* 32
Figure S35. *J*-based configurational analysis of six hypothetical rotamers represents 11*R*, 13*S*......... 32
Figure S36. ^{1}H, ^{13}C HSQC-Hecade NMR spectrum of colposetin C in CD_3OD (700 MHz).............. 33
Figure S37. ^{1}H, ^{13}C *J*-HMBC NMR spectrum of colposetin C in CD_3OD (700 MHz)........................ 34
Figure S38. HPLC-DAD/MS chromatogram of colpomenoic acid A... 35
Figure S39. HR-ESIMS chromatogram of colpomenoic acid A 35
Figure S40. UV/vis spectrum of colpomenoic acid A in MeOH... 35
Figure S41. ^{1}H NMR spectrum of colpomenoic acid A in CD_3OD (700 MHz)................................. 36
Figure S42. ^{13}C NMR spectrum of colpomenoic acid A in CD_3OD (700 MHz)................................ 37
Figure S43. $^{1}H,^{1}H$ COSY NMR spectrum of colpomenoic acid A in CD_3OD (700 MHz).................. 38
Figure S44. $^{1}H,^{13}C$ HSQC-DEPT NMR spectrum of colpomenoic acid A in CD_3OD (700 MHz, 176 MHz) 39
Figure S45. $^{1}H,^{13}C$ HMBC NMR spectrum of colpomenoic acid A in CD_3OD (700 MHz, 176 MHz)40
Figure S46. ROESY NMR spectrum of colpomenoic acid A in CD_3OD (700 MHz).......................... 41
Figure S47. HPLC-DAD/MS chromatogram of colpomenoic acid B.. 42
Figure S48. HR-ESIMS chromatogram of colpomenoic acid B 42
Figure S49. UV/vis spectrum of colpomenoic acid B in MeOH (log ε) [neutral]) λ_{max} (log ε) 229 (4.41), 286 (3.97) nm.. 42
Figure S50. ^{1}H NMR spectrum of colpomenoic acid B in CD_3OD (700 MHz)................................. 43
Figure S51. ^{13}C NMR spectrum of colpomenoic acid B in CD_3OD (700 MHz)................................ 44
Figure S52. $^{1}H,^{1}H$ COSY NMR spectrum of colpomenoic acid B in CD_3OD (700 MHz) 45
Figure S53. $^{1}H,^{13}C$ HSQC-DEPT NMR spectrum of colpomenoic acid B in CD_3OD (700 MHz, 176 MHz) 46
Figure S54. $^{1}H,^{13}C$ HMBC NMR spectrum of colpomenoic acid B in CD_3OD (700 MHz, 176 MHz)47
Figure S55. ROESY NMR spectrum of colpomenoic acid B in CD_3OD (700 MHz).......................... 48
Figure S56. Graph for calculating IC_{50} of colposetin B against mouse fibroblasts L-929 49
Figure S57. Graph for calculating IC_{50} of colposetin B against human endocervical adenocarcinoma KB-3.1 50
Figure S58. Graph for calculating IC_{50} of colposetin B against human breast adenocarcinoma MCF-7 51
Figure S59. Graph for calculating IC_{50} of colposetin B against human lung carcinoma A-549 52
Figure S60. Graph for calculating IC_{50} of colposetin B against human prostate cancer PC-3 53
Figure S61. Graph for calculating IC_{50} of colposetin B against ovarian carcinoma SK-OV-3 54
Figure S62. Graph for calculating IC_{50} of colposetin B against squamous cell carcinoma A-431....... 55

Protocol: Antimicrobial Activity Assay 56
Protocol: Cytotoxicity Assay 57

Morphological analysis and molecular identification

Colony diameter of *Colpoma quercinum* CCTU A372 was 15, 20 and 45 mm in 14 days at 25 °C on PDA, MEA and OA medium growing, respectively. Colonies were flat on PDA and OA and slightly raise on MEA. Colony colour on the surface of PDA and MEA was brown and light brown to yellow on OA. Colony colour on the reverse was dark brown to dark on PDA and MEA, respectively and colony colour was light brown to yellow on the reverse of OA (Figure S1).

Megablast search analysis at NCBI's GenBank nucleotide database showed high similarity with reference sequence of *Colpoma* from GenBank. A phylogeny inferred based on combination of ITS-rDNA and *LSU* gene sequence data obtained in this study together with sequence data from GenBank (Table S1). The final sequence alignment of the ITS-rDNA sequence and *LSU* gene comprising 20 internal taxa had 1078 characters (ITS-rDNA: 1-528, LSU: 529-1078) and 389 unique site patterns (ITS-rDNA: 248, LSU: 141). *Potebniamyces_pyri* was served as the outgroup taxon. Bayesian analyses were performed using the best-fitting substitution (GTR+G+I for ITS-rDNA and SYM+I+G for LSU) model and resulted in 892 generations. After discarding the first 25% of generations as burn-in, the remaining 670 (75%) generations were used to calculate the consensus Bayesian tree and posterior probabilities. Results indicated that the isolate used in this study clustered together with *Colpoma quercinum* in same clade with highly supported value (Figure S2). There are about 30 species of *Colpoma* known worldwide, which are distinguished according to the characteristics of Ascomata, asci and ascospores on host plants. Sequence data are only available for *Colpoma quercinum* and considering the fact that this strain have isolated as endophytic, the morphological characteristics of sexual stage of this species on the woody host is not available for comparison with other species of *Colpoma*. But, results indicated that the isolate used in this study clustered together with *Colpoma quercinum* in same clade with highly supported value.

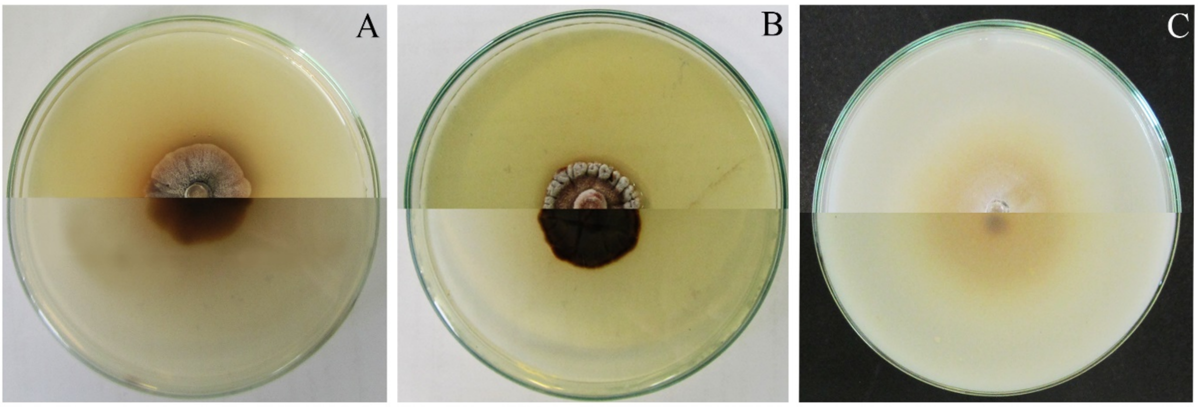

Figure S1. Morphological characteristics of *C. quercinum* CCTU A372. A-C: 14 days old culture on PDA (A), MEA (B) and OA (C) (the upper half: colony on the surface; the lower half: colony on the reverse)

Table S1. List of reference taxa and corresponding reference sequences selected for the molecular phylogeny.

Species	GenBank Acc No	
	ITS	LSU
Coccomyces dentatus	AY544657	DQ491499
Colpoma quercinum	EU833991	U92306
Cudonia circinans	AY533013	EU784190
Cudonia lutea	AF433140	AF433150
Cudonia sichuanensis	AF433137	AF433147
Cyclaneusma minus	FJ176868	AF013222
Lophodermium pinastri	AY004334	AY422490
Meria laricis	DQ470954	U92299
Rhytisma acerinum	FJ495190	GQ253100
Rhytisma salicinum	FJ495191	AY465516
Rhytisma huangshanense	FJ495192	GQ253101
Spathularia flavida	AY541496	AF433155
Tryblidiopsis pinastri	DQ470983	U92307

Lophodermium autumnale	HQ902151	HQ902158
Lirula macrospore	HQ902152	HQ902159
Lophodermium piceae	HQ902153	HQ902160
Lirula yunnanensis	HQ902149	HQ902156
Lirula exigua	HQ902148	HQ902155
Lirula exigua	HQ902150	HQ902157
Lirula exigua	HQ902147	HQ902154
Potebniamyces pyri	DQ470949	AY608642
Colpoma quercinum* CCTU A372		

* Isolate used in this study.

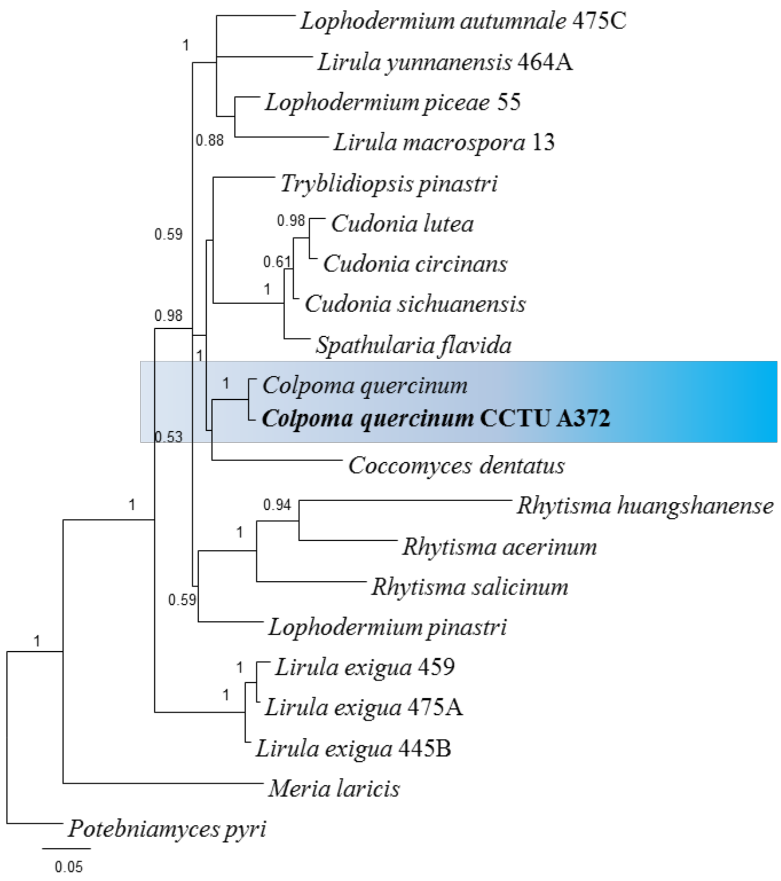

Figure S2. Consensus phylogram (75% majority rule) of 670 trees resulting from a Bayesian analysis of ITS-rDNA region and *LSU* gene sequence alignment using MrBayes v. 3.2.2. The scale bar indicates 0.05 expected changes per site. The tree was rooted to *Potebniamyces_pyri*

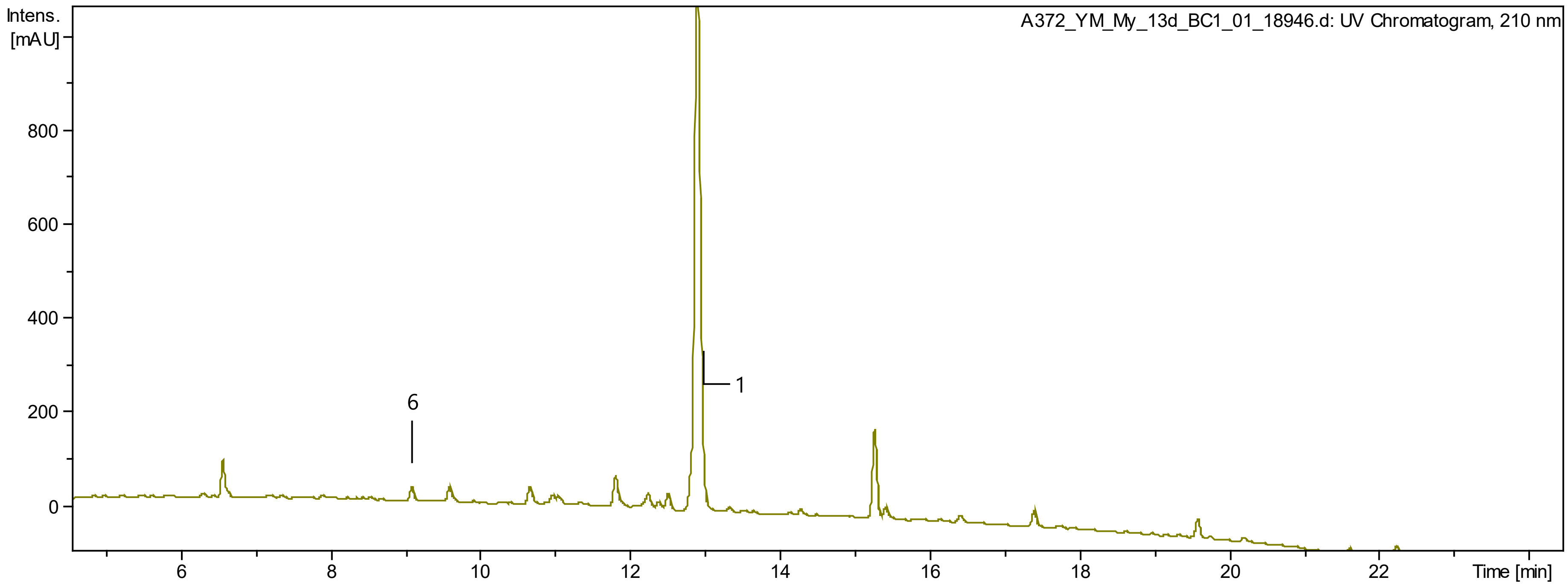

Figure S3. HPLC-UV/Vis chromatogram at 210 nm of the mycelial crude extract of *C. quercinum* CCTU A372 showing colposetin A (**1**) and penicillide (**6**). Monodictyphenone (**7**) was not detected in the crude extract.

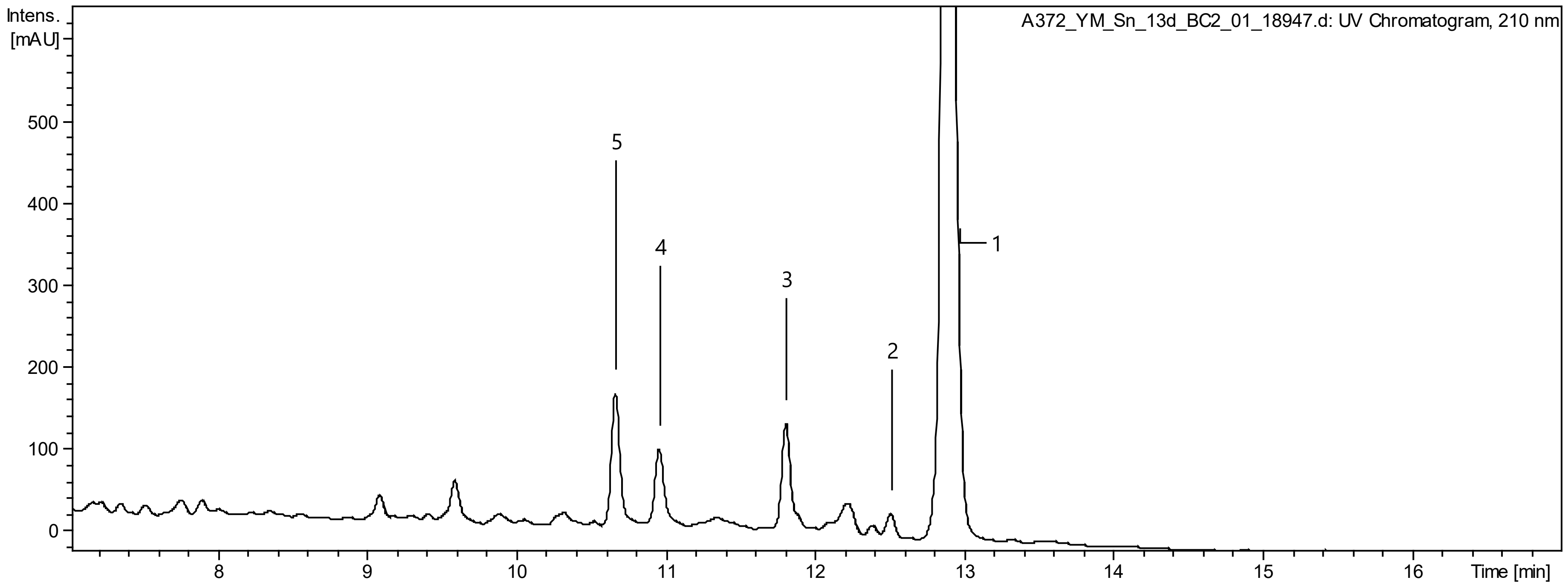

Figure S4. HPLC-UV/Vis chromatogram at 210 nm of the supernatant crude extract of *C. quercinum* CCTU A372. Colposetin A-C: **1-3**, colpomenoic acid A: **4** and colpomenoic acid B: **5**.

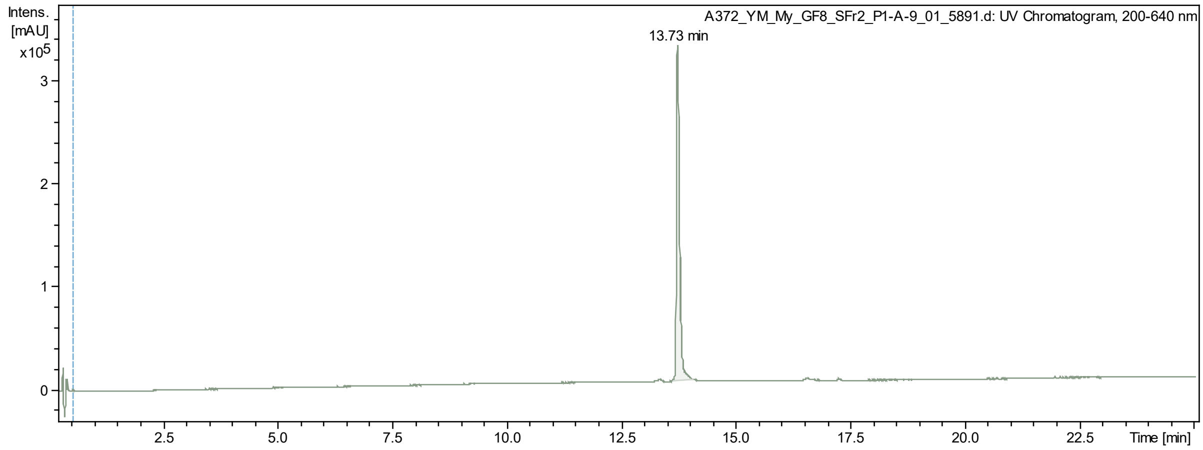

Figure S5. HPLC-DAD/MS chromatogram of colposetin A

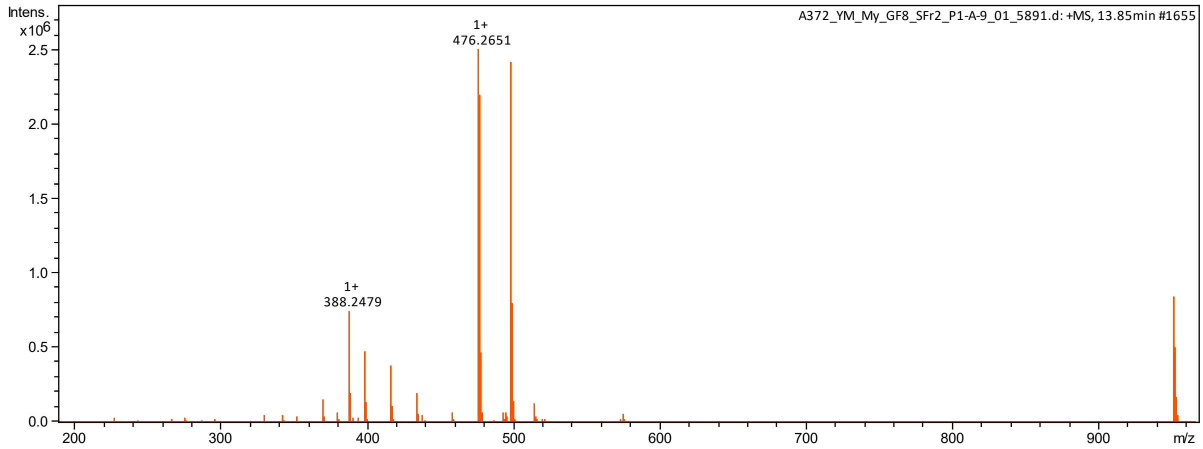

Figure S6. HR-ESIMS chromatogram of colposetin A

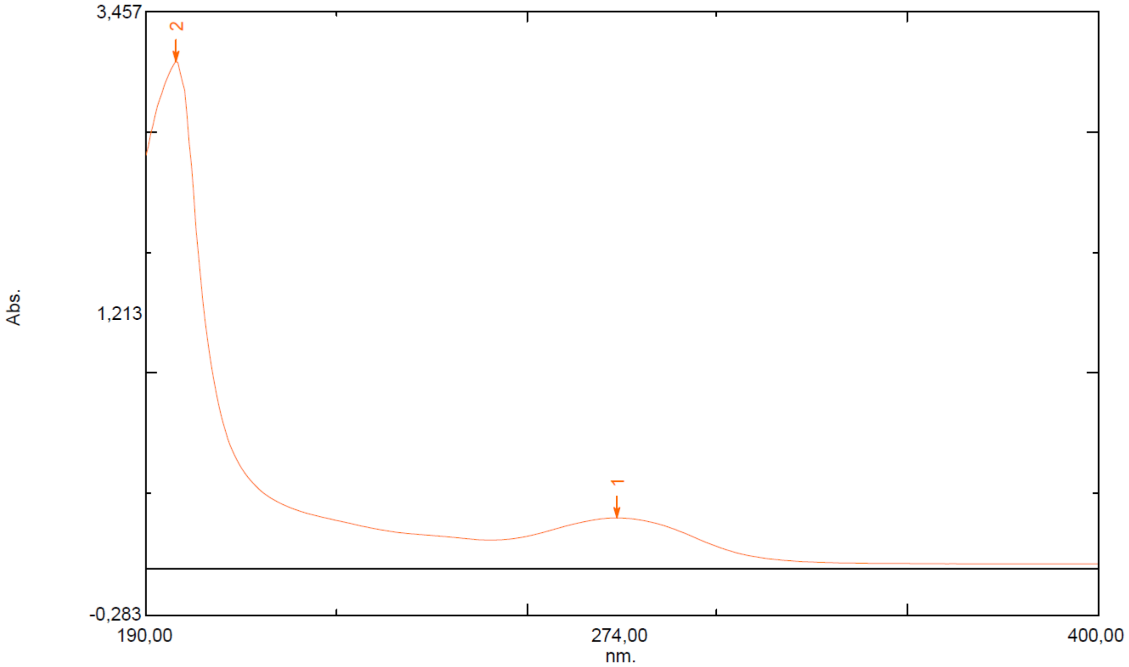

Figure S7. UV/vis spectrum of colposetin A in MeOH (log ε) [neutral] λ_{max} (log ε) 266 (3.62), 294 (3.71) nm.

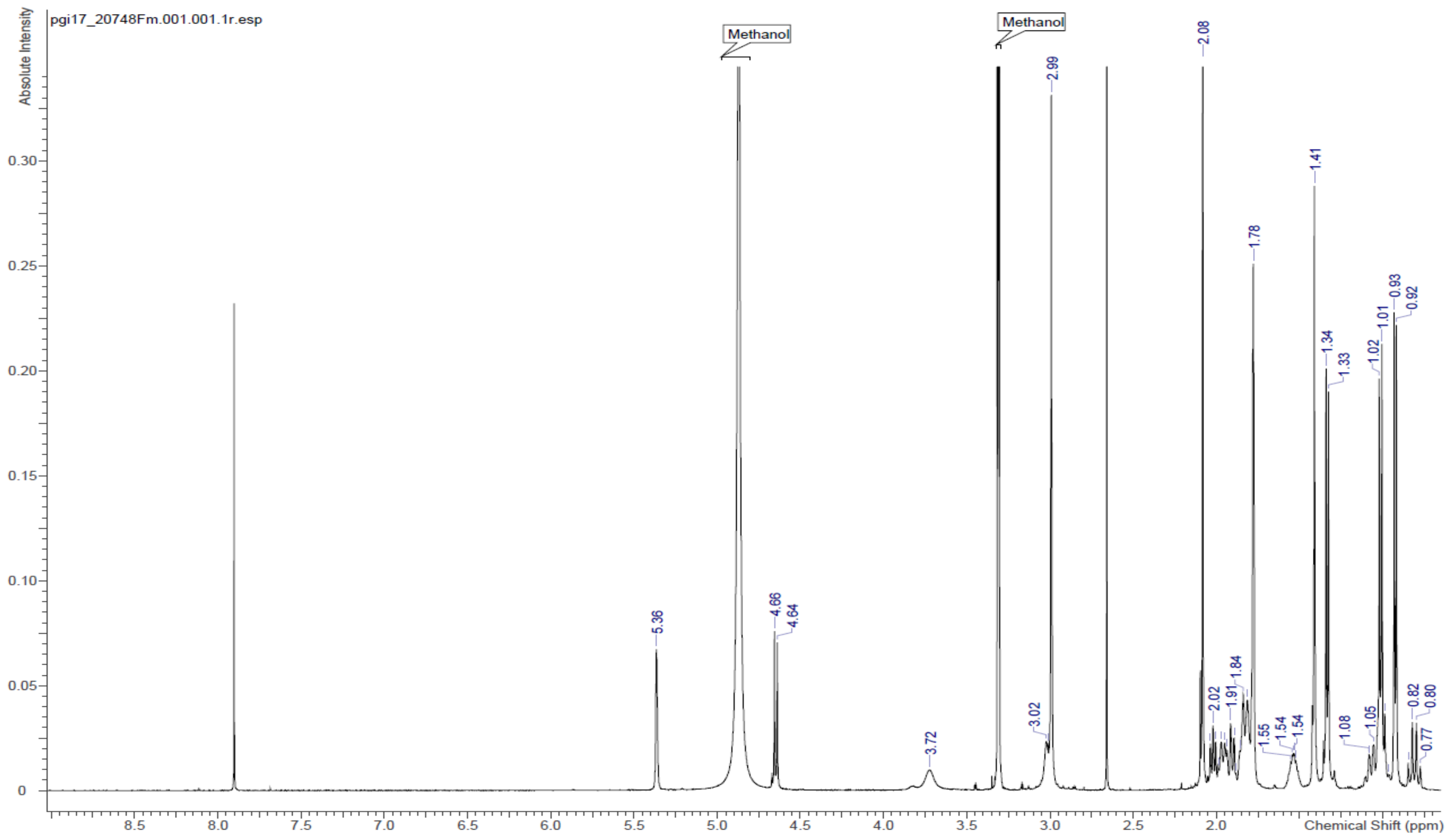

Figure S8. ^{1}H NMR spectrum of colposetin A in CD_3OD (700 MHz)

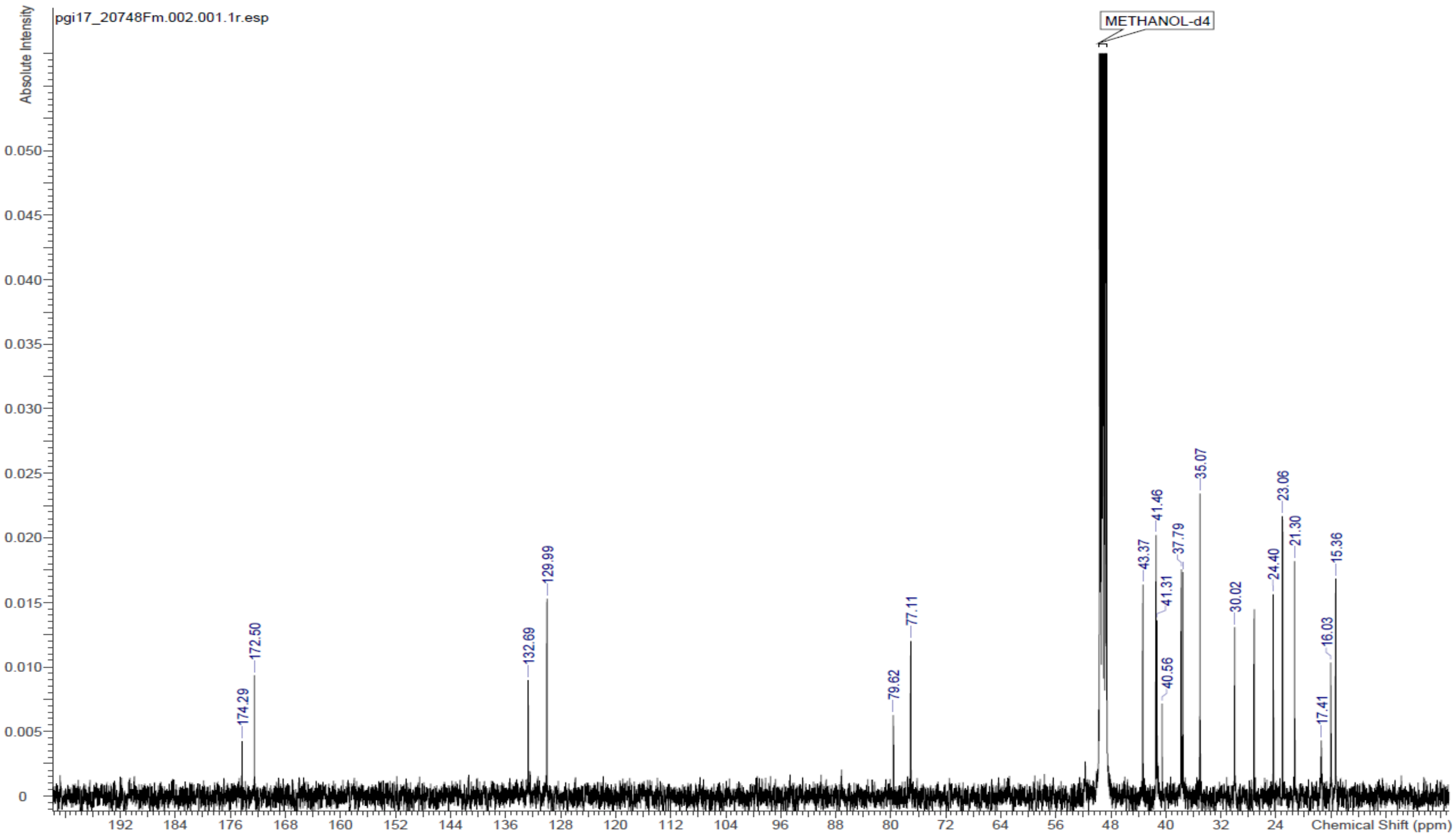

Figure S9. ^{13}C NMR spectrum of colposetin A in CD_3OD (700 MHz)

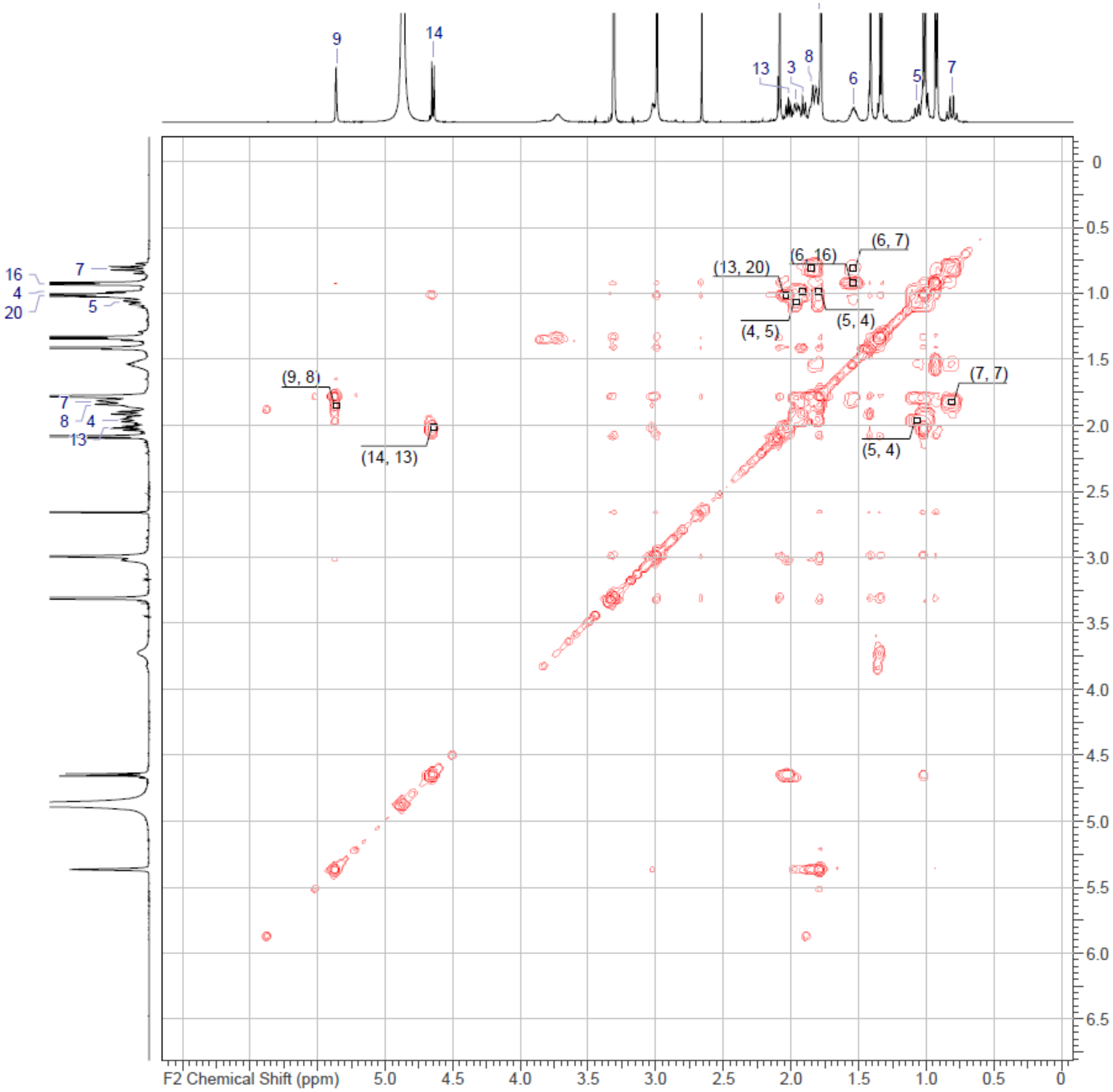

Figure S10. $^{1}H,^{1}H$ COSY NMR spectrum of colposetin A in CD_3OD (700 MHz)

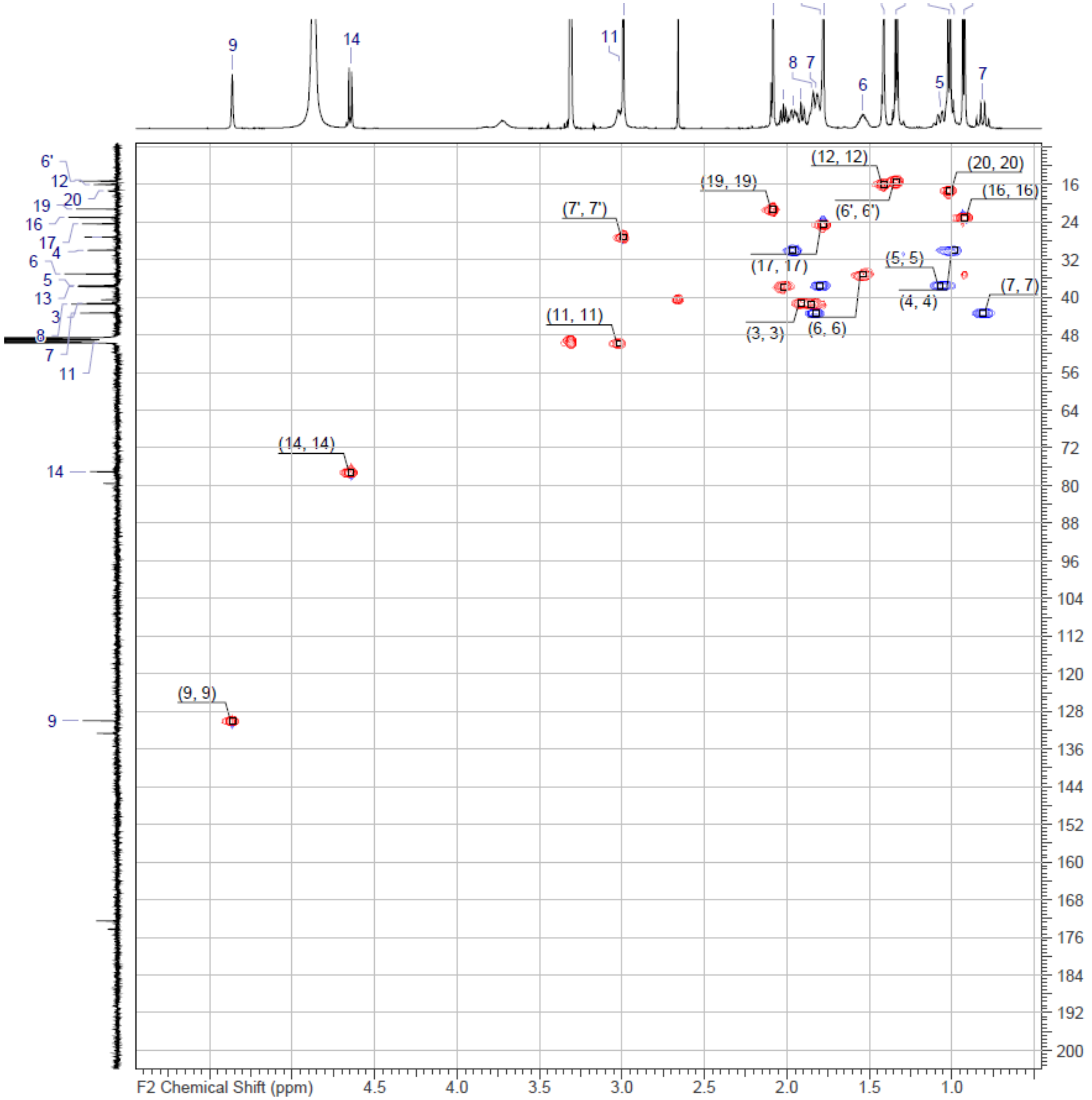

Figure S11. ^{1}H,^{13}C HSQC-DEPT NMR spectrum of colposetin A in CD_3OD (700 MHz, 176 MHz)

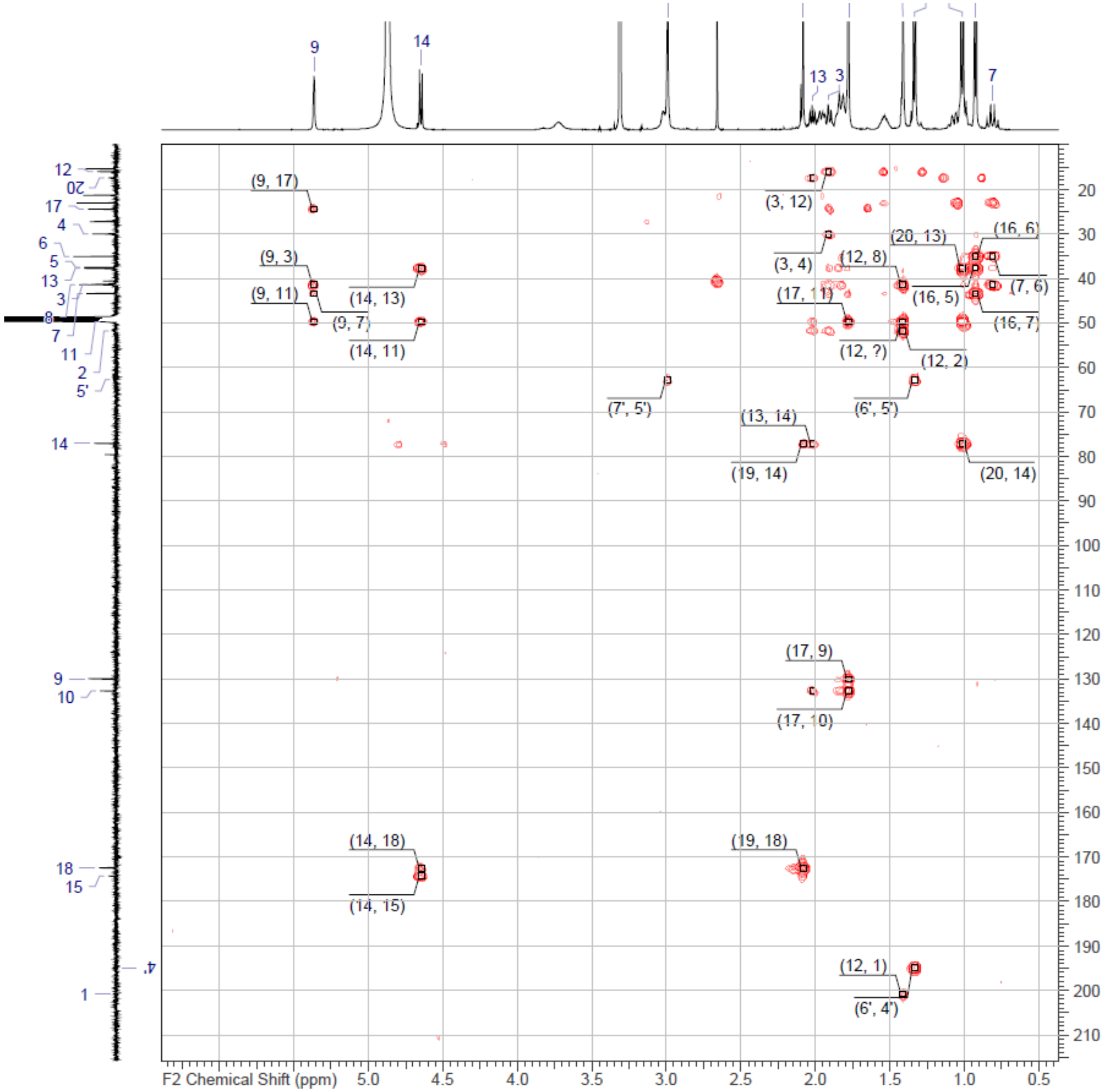

Figure S12. ^{1}H,^{13}C HMBC NMR spectrum of colposetin A in CD_3OD (700 MHz, 176 MHz)

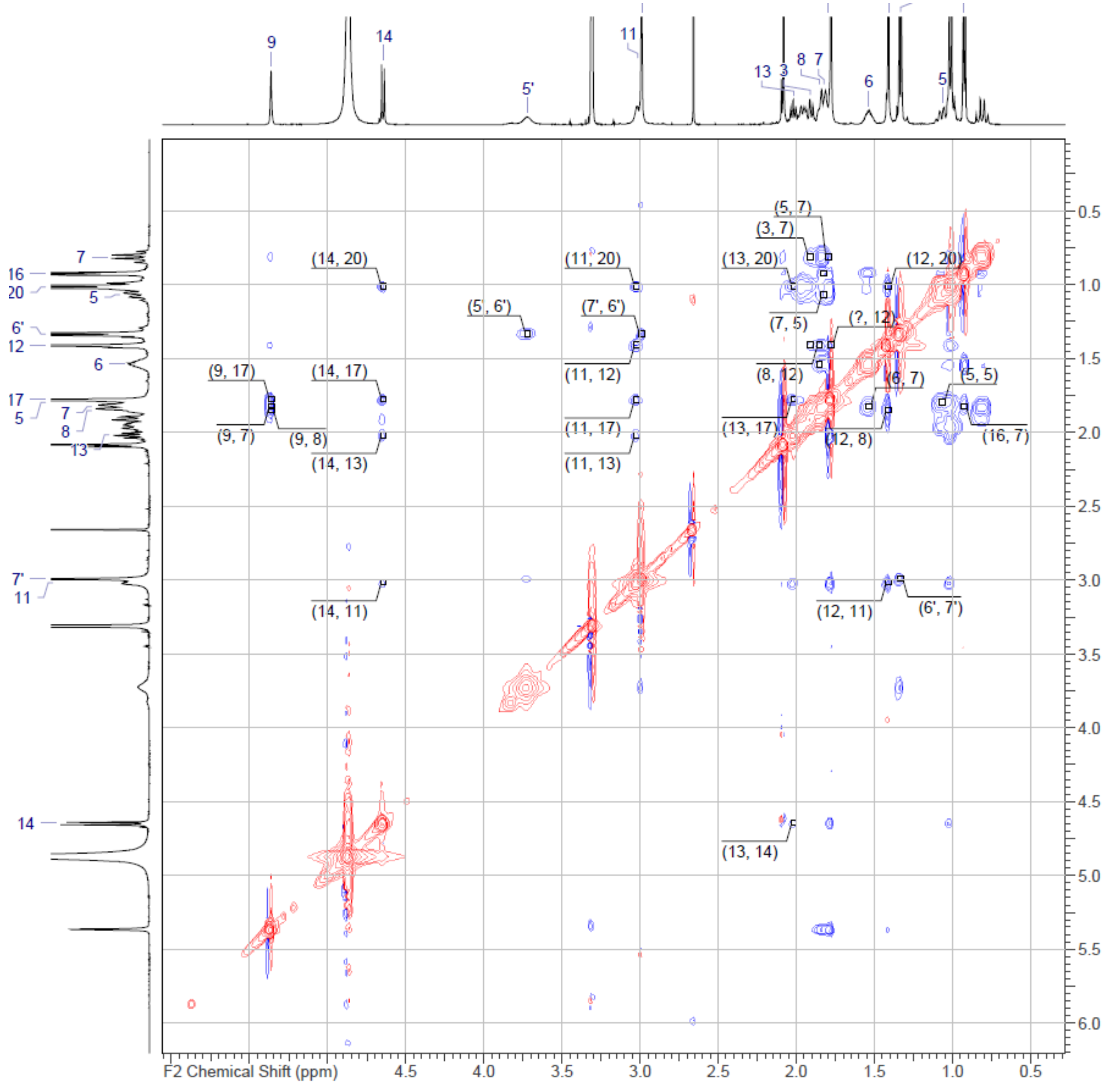

Figure S13. NOESY NMR spectrum of colposetin A in CD_3OD (700 MHz)

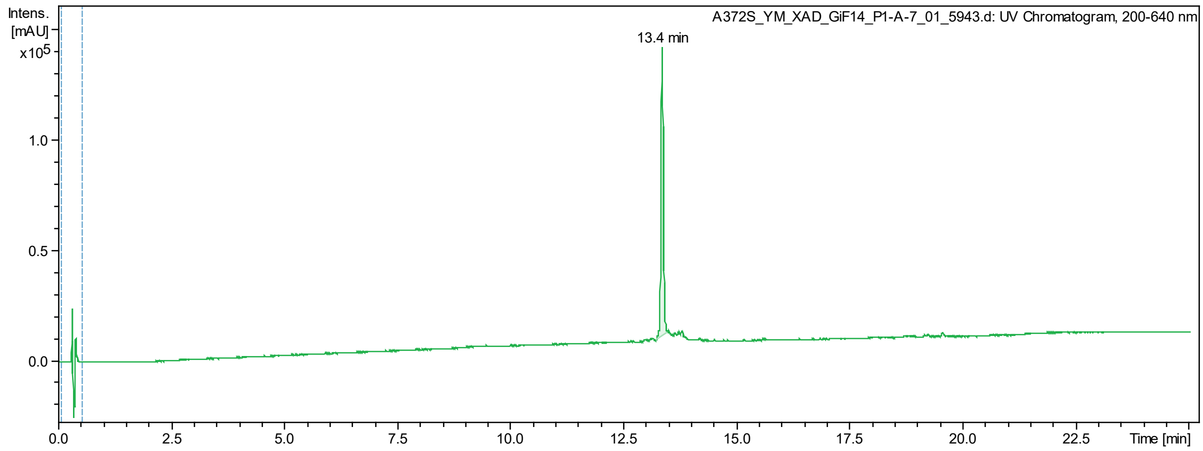

Figure S14. HPLC-DAD/MS chromatogram of colposetin B

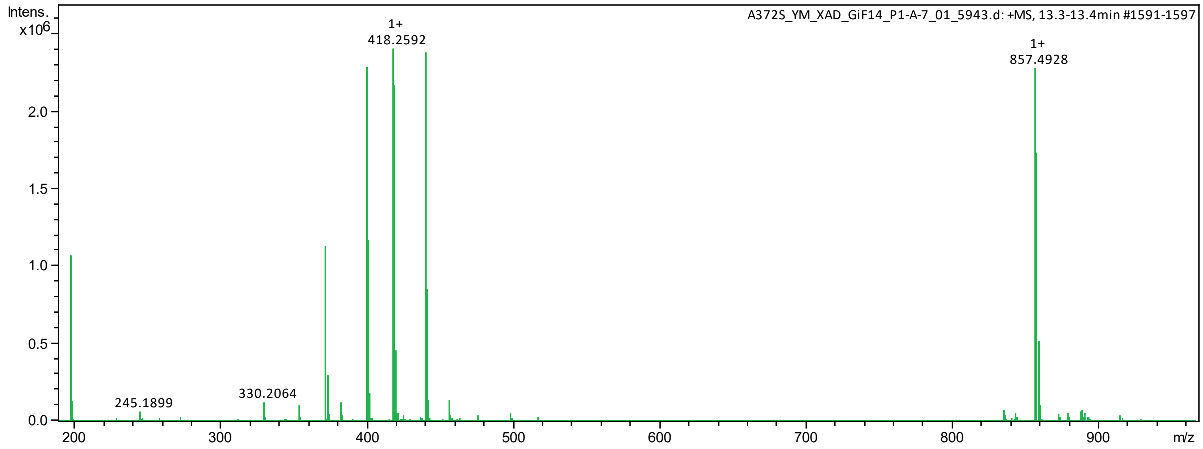

Figure S15. HR-ESIMS chromatogram of colposetin B

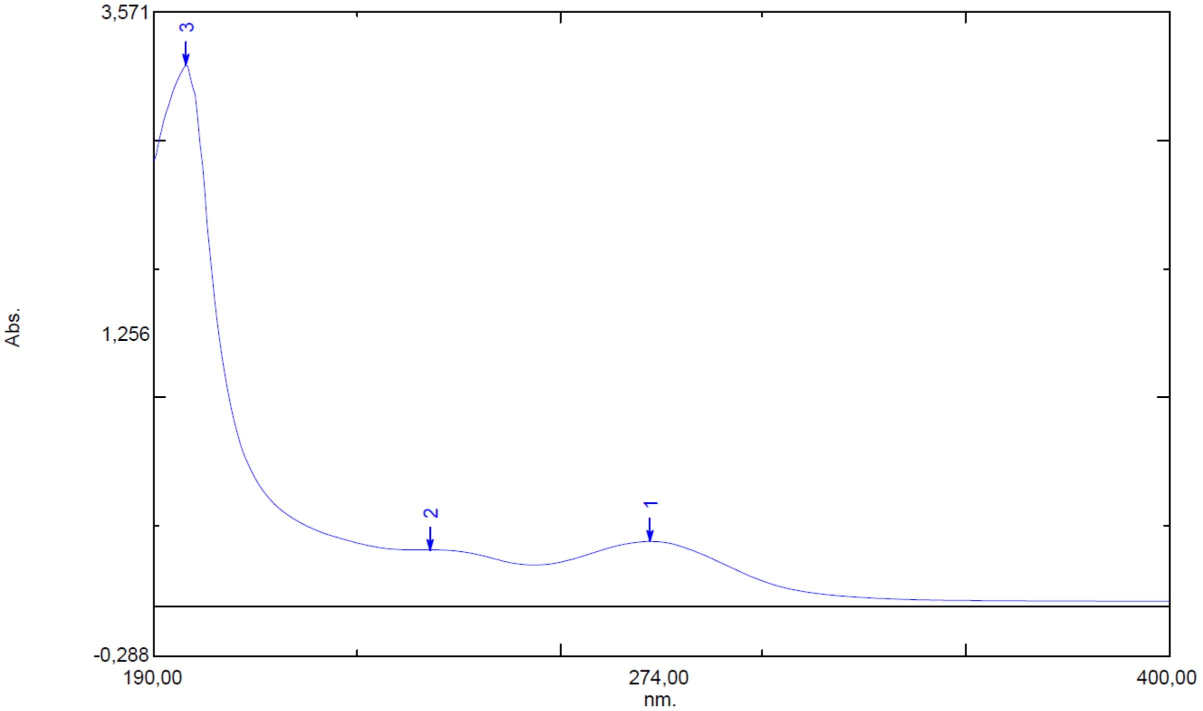

Figure S16. UV/vis spectrum of colposetin B in MeOH (log ε) [neutral] λ_{max} (log ε) 247 (3.85), 291 (3.91) nm

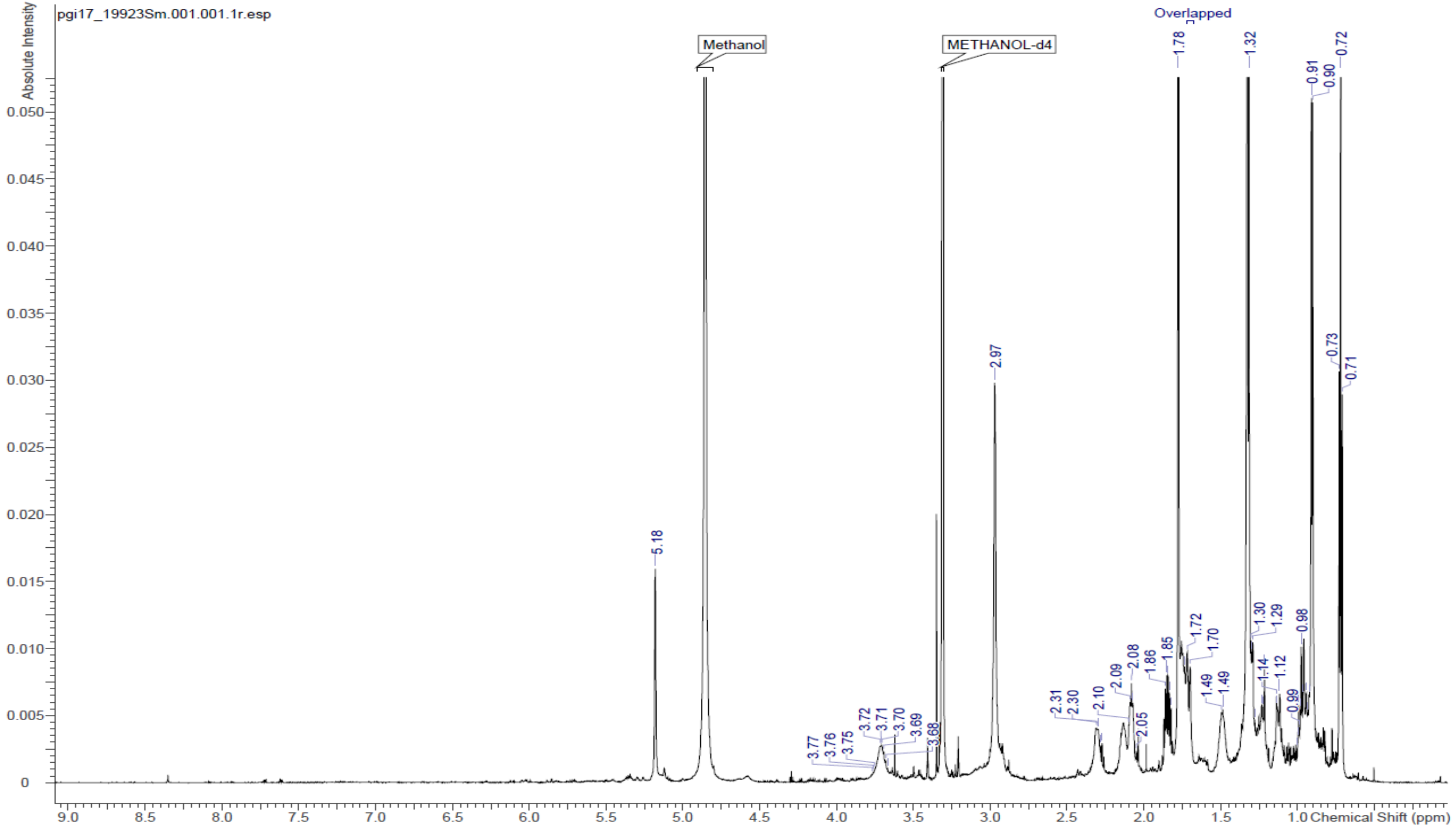

Figure S17. ^{1}H NMR spectrum of colposetin B in CD_3OD(700 MHz)

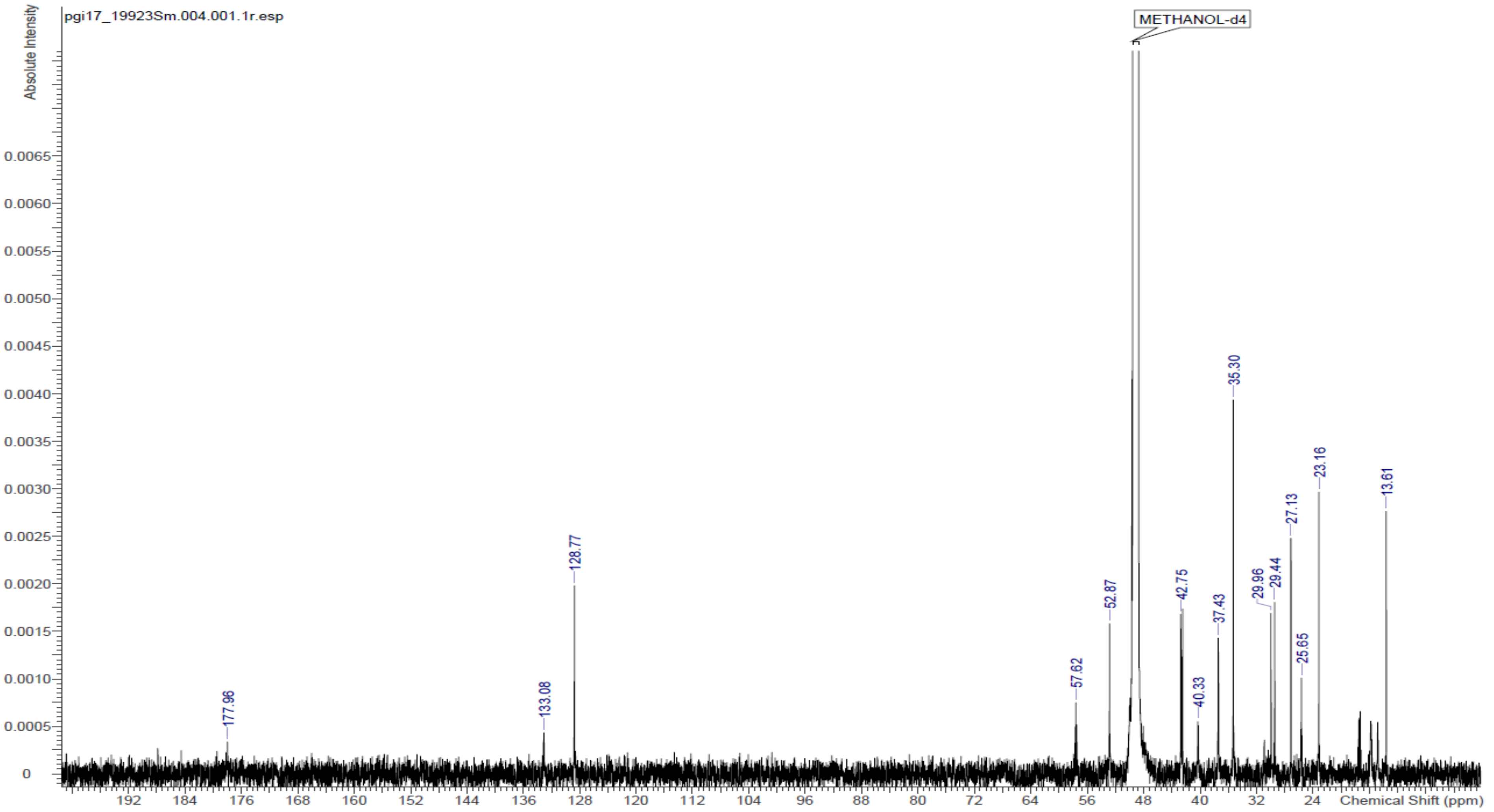

Figure S18. ^{13}C NMR spectrum of colposetin B in CD_3OD (700 MHz)

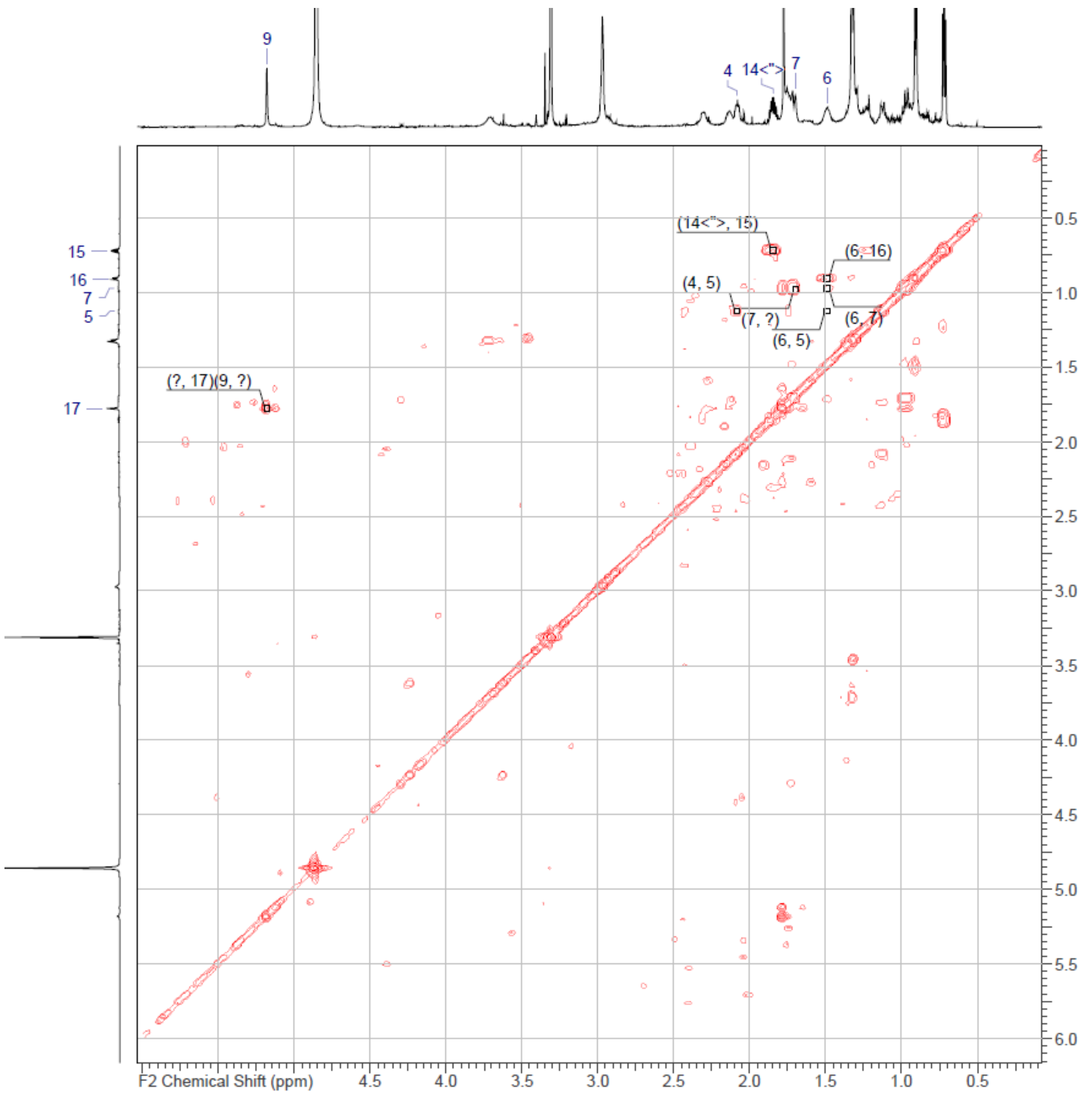

Figure S19. ^{1}H,^{1}H COSY NMR spectrum of colposetin B in CD_3OD (700 MHz)

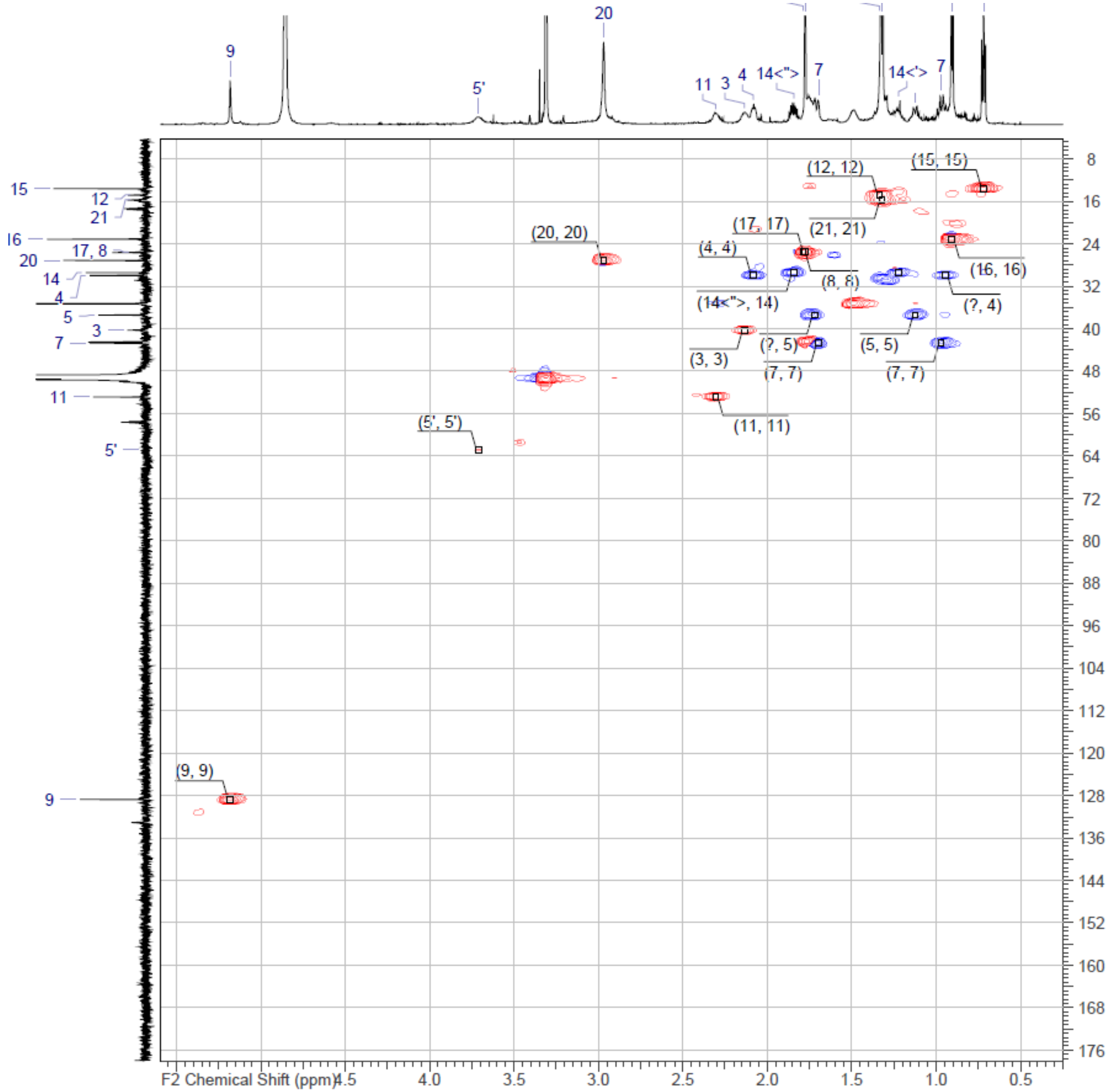

Figure S20. ^{1}H,^{13}C HSQC-DEPT NMR spectrum of colposetin B in CD_3OD (700 MHz, 176 MHz)

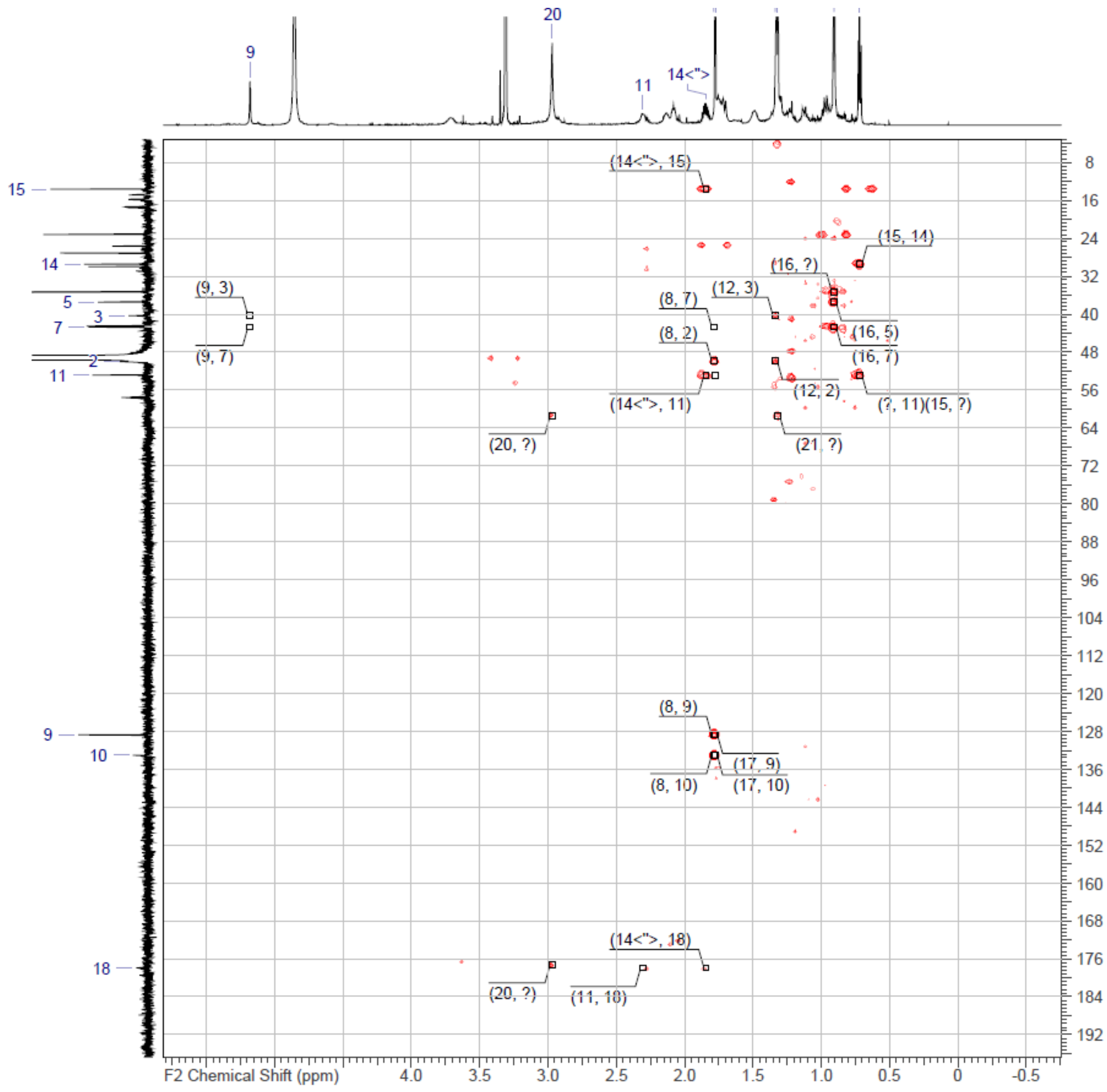

Figure S21. ^{1}H,^{13}C HMBC NMR spectrum of colposetin B in CD_3OD (700 MHz, 176 MHz)

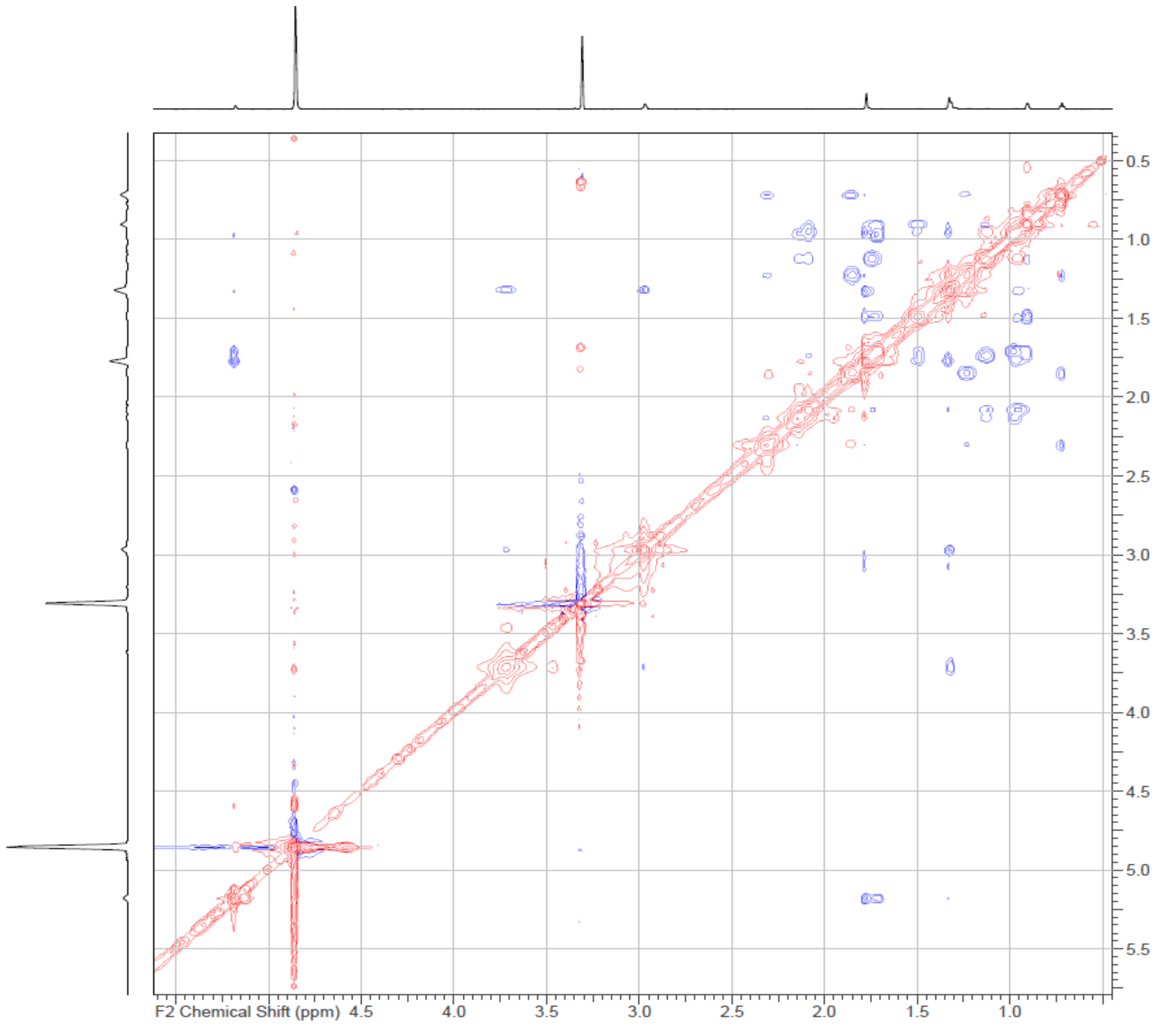

Figure S22. NOESY NMR spectrum of colposetin B in CD_3OD (700 MHz)

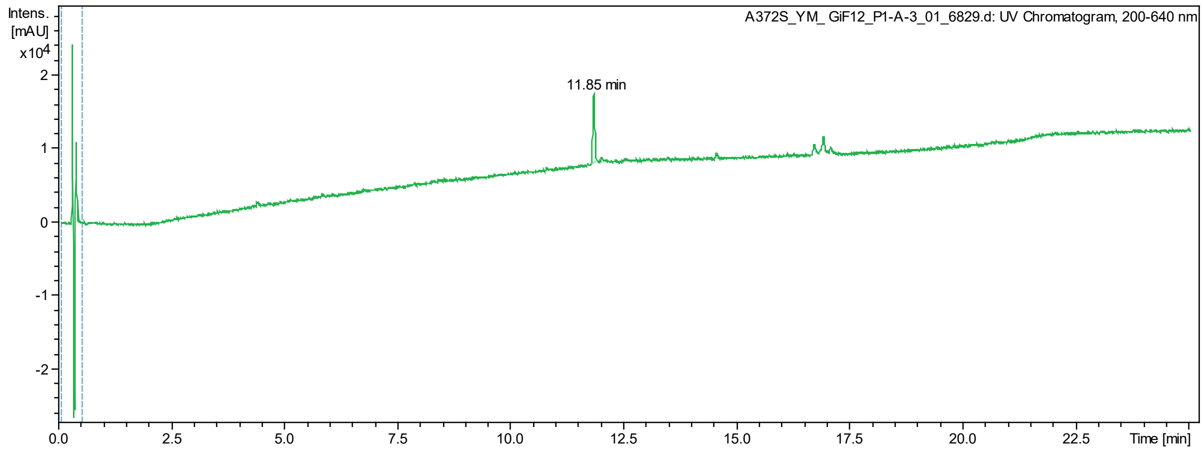

Figure S23. HPLC-DAD/MS chromatogram of colposetin C

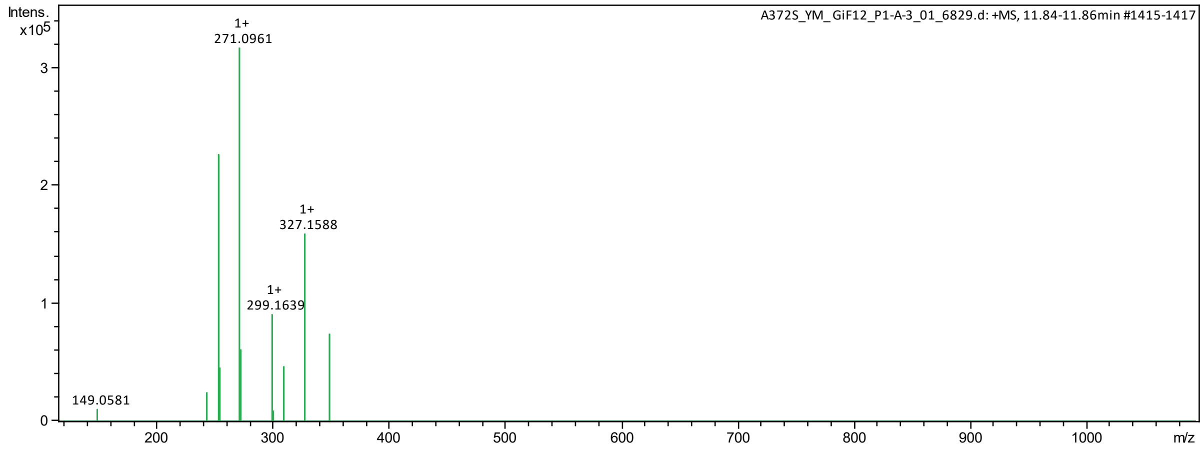

Figure S24. HR-ESIMS chromatogram of colposetin C

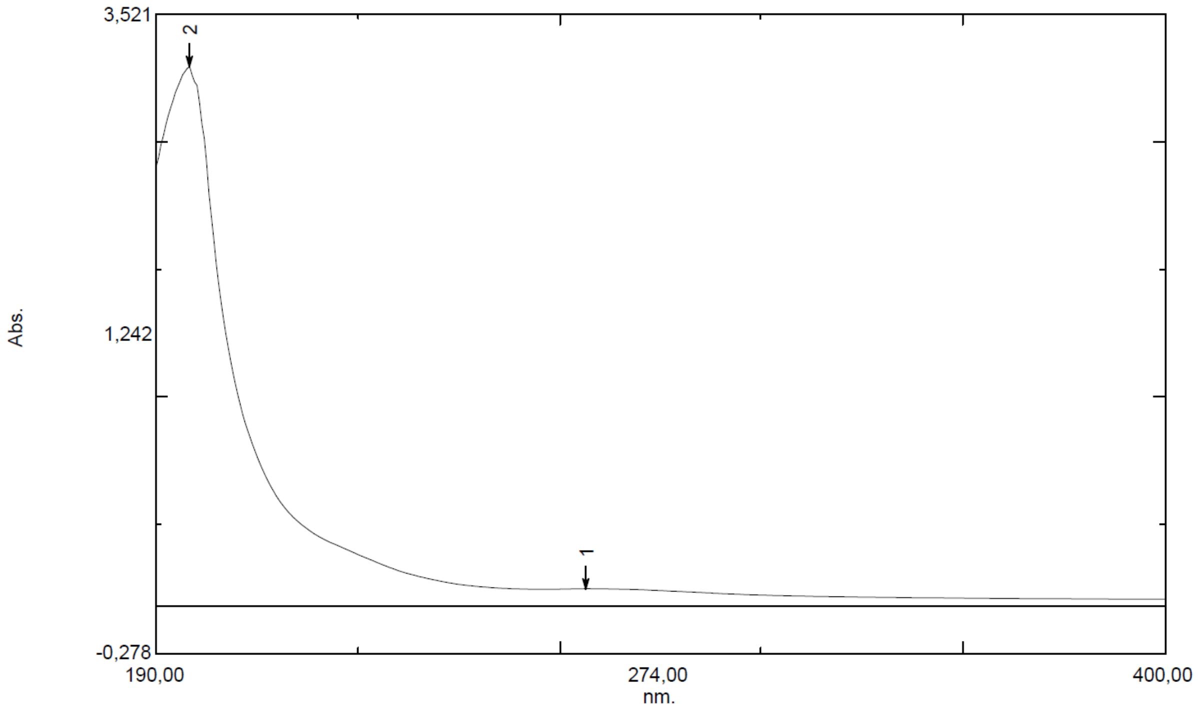

Figure S25. UV/vis spectrum of colposetin C in MeOH (log ε) [neutral] λ_{max} (log ε) 279 (3.19) nm

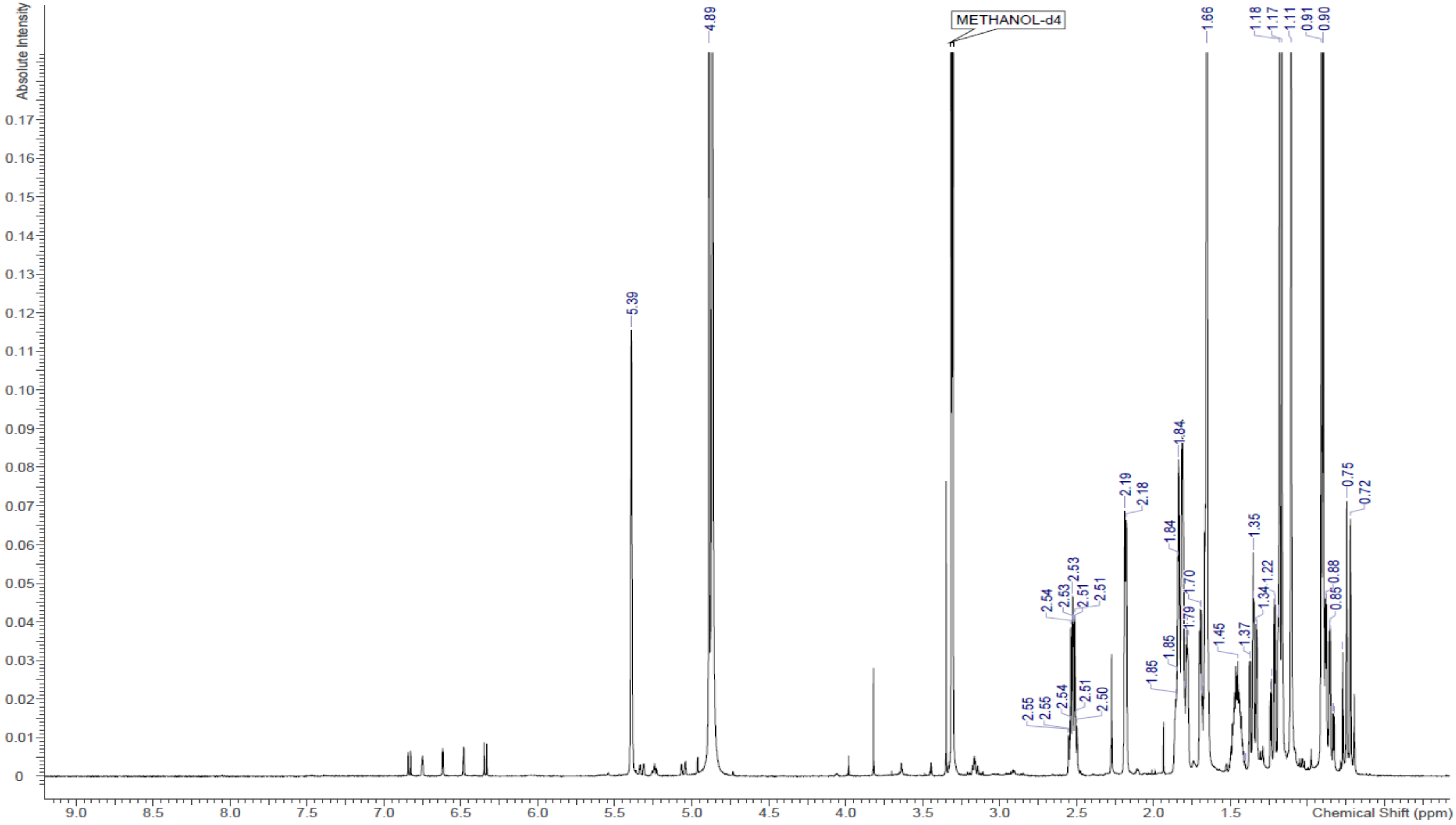

Figure S26. ^{1}H NMR spectrum of colposetin C in CD_3OD (700 MHz)

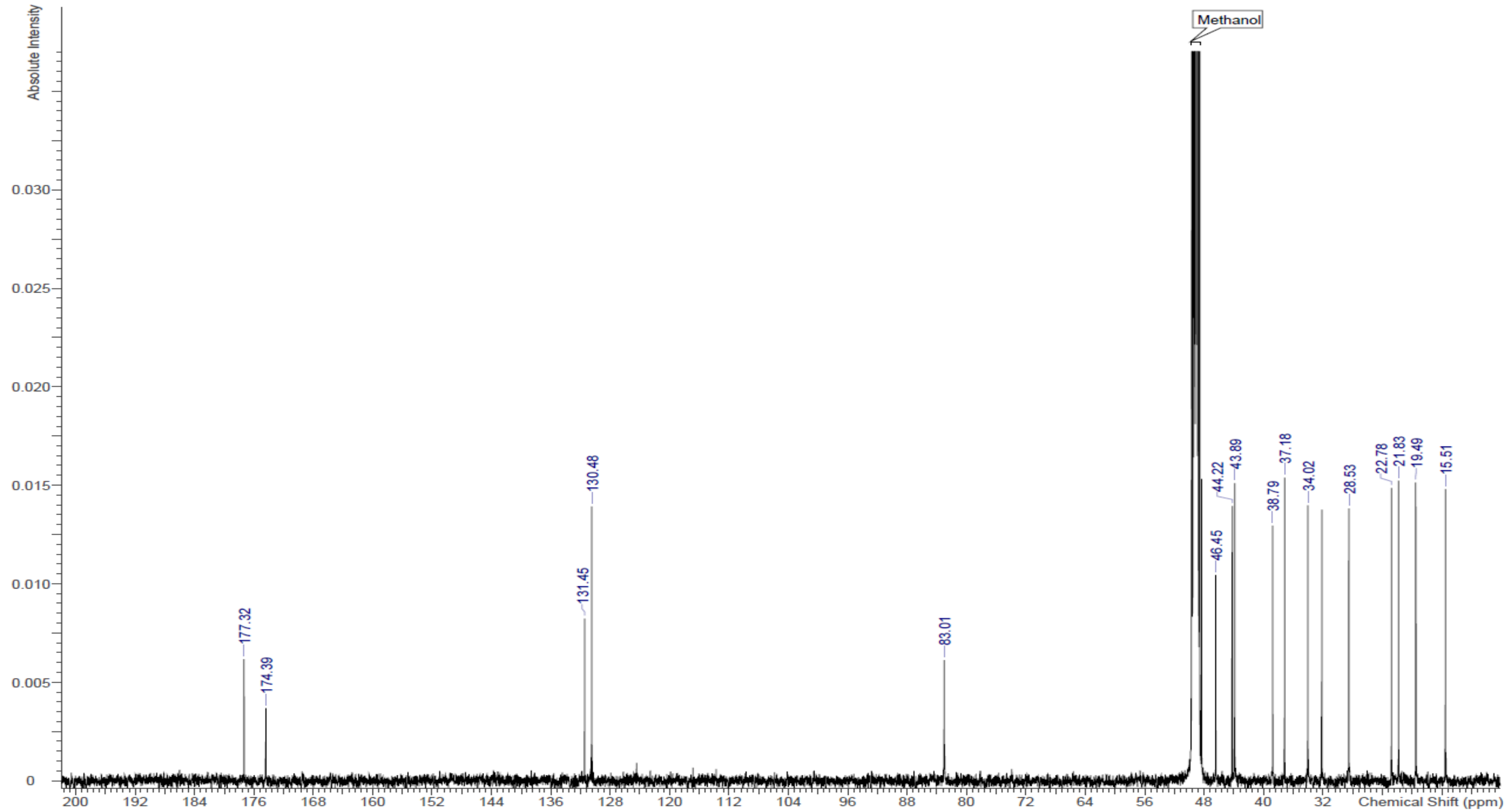

Figure S27. ^{13}C NMR spectrum of colposetin C in CD_3OD (700 MHz)

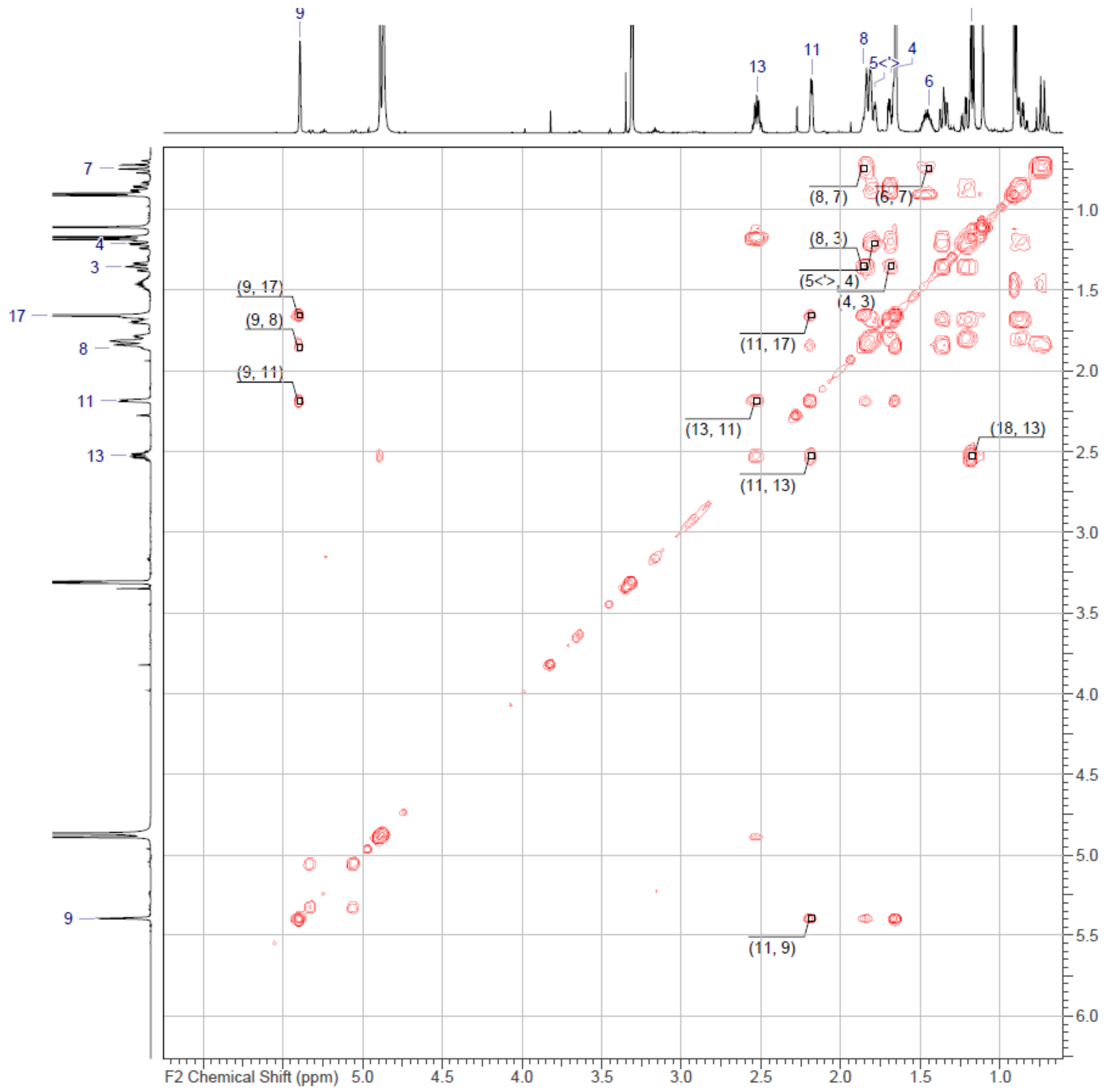

Figure S28. ^{1}H,^{1}H COSY NMR spectrum of colposetin C in CD_3OD (700 MHz)

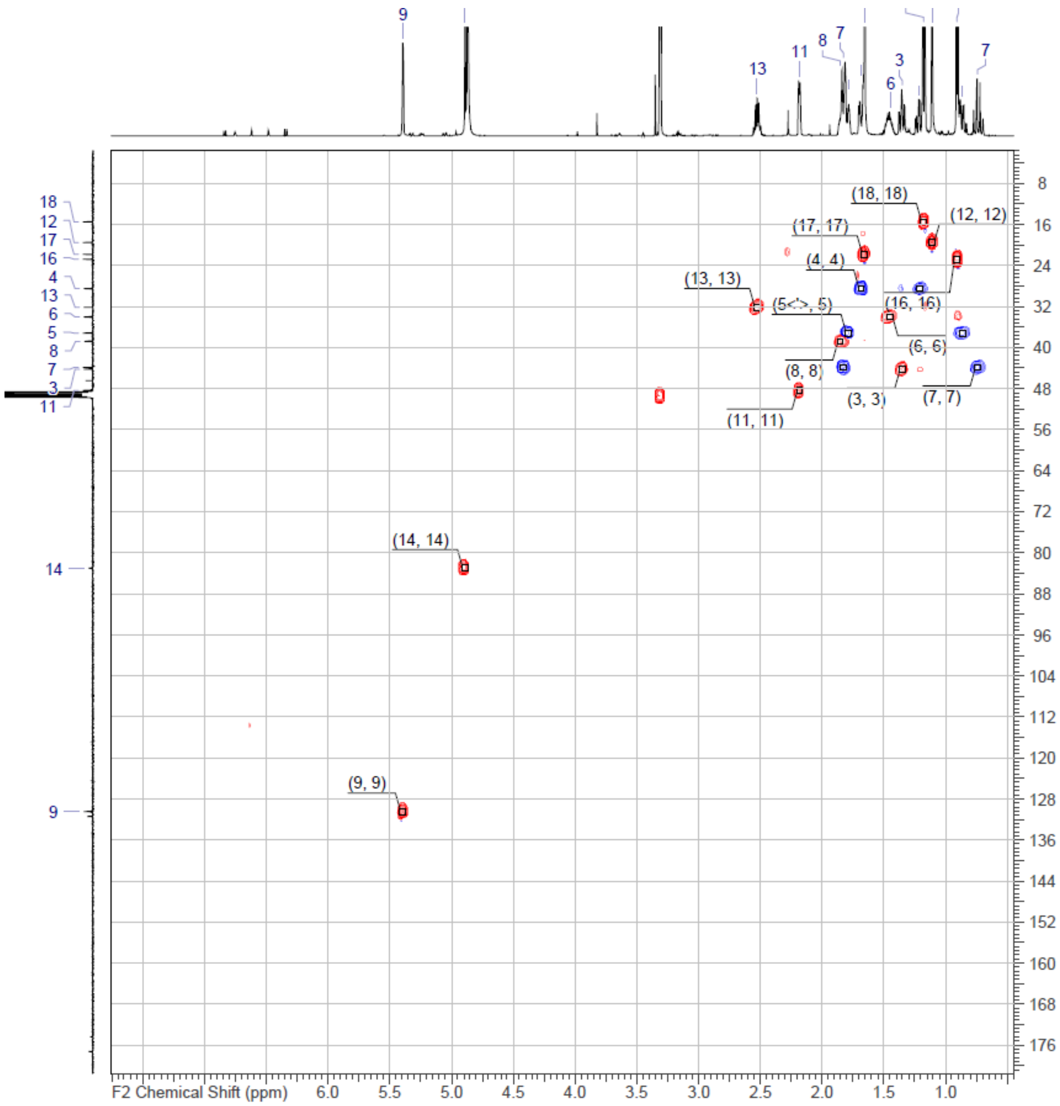

Figure S29. ^{1}H,^{13}C HSQC-DEPT NMR spectrum of colposetin C in CD3OD (700 MHz, 176 MHz)

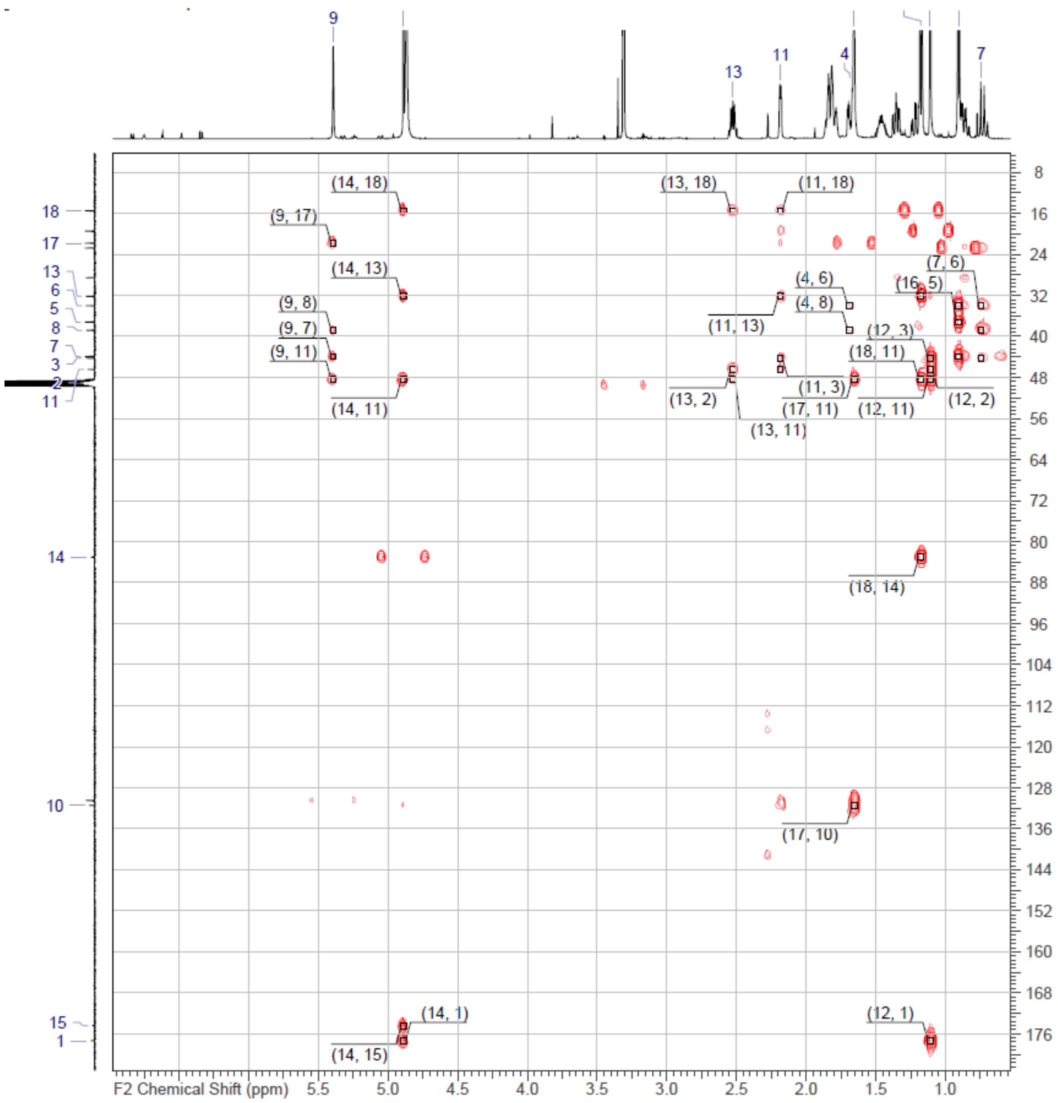

Figure S30. $^{1}H,^{13}C$ HMBC NMR spectrum of colposetin C in CD_3OD (700 MHz, 176 MHz)

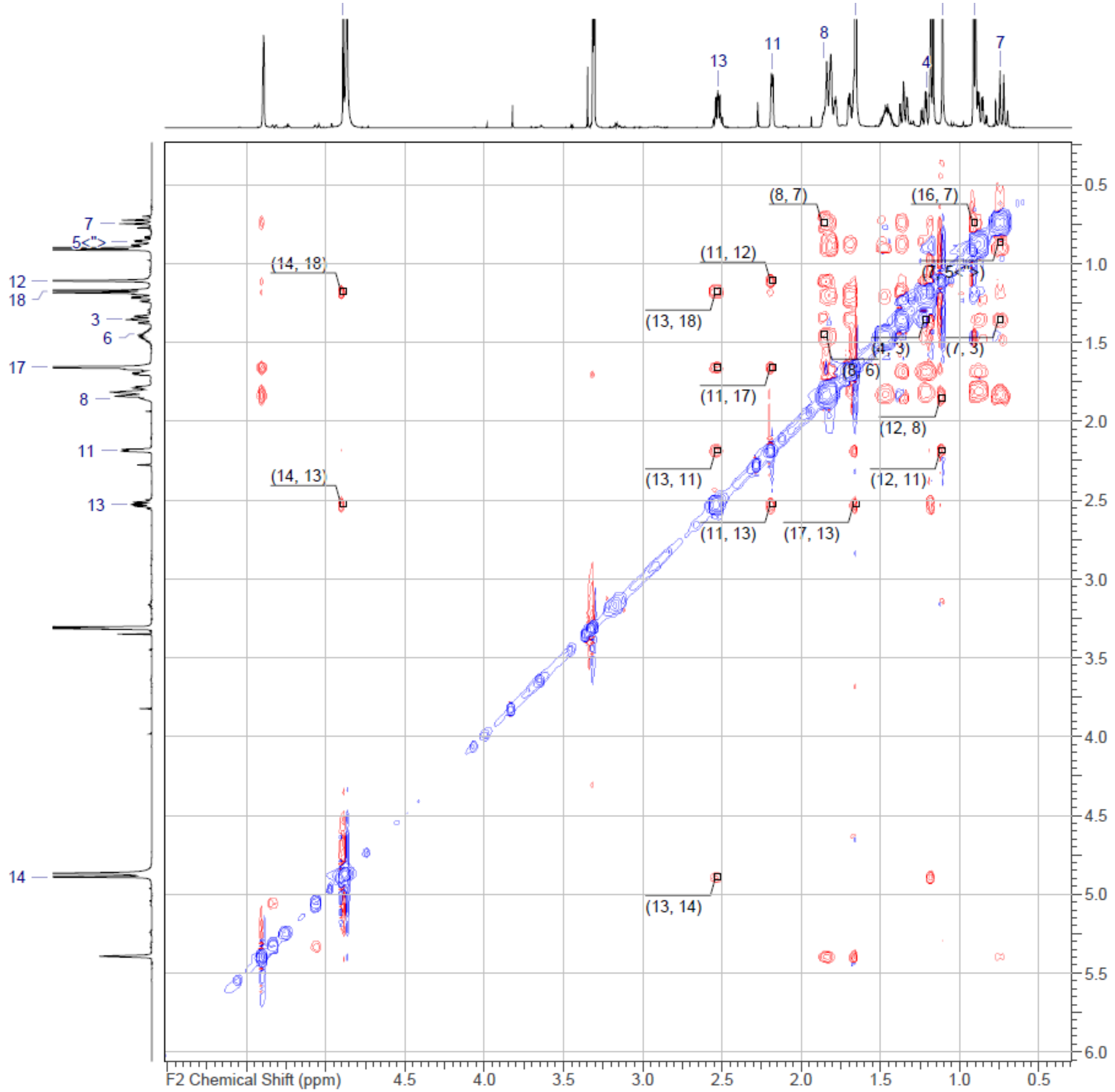

Figure S31. ROESY NMR spectrum of colposetin C in CD_3OD (700 MHz)

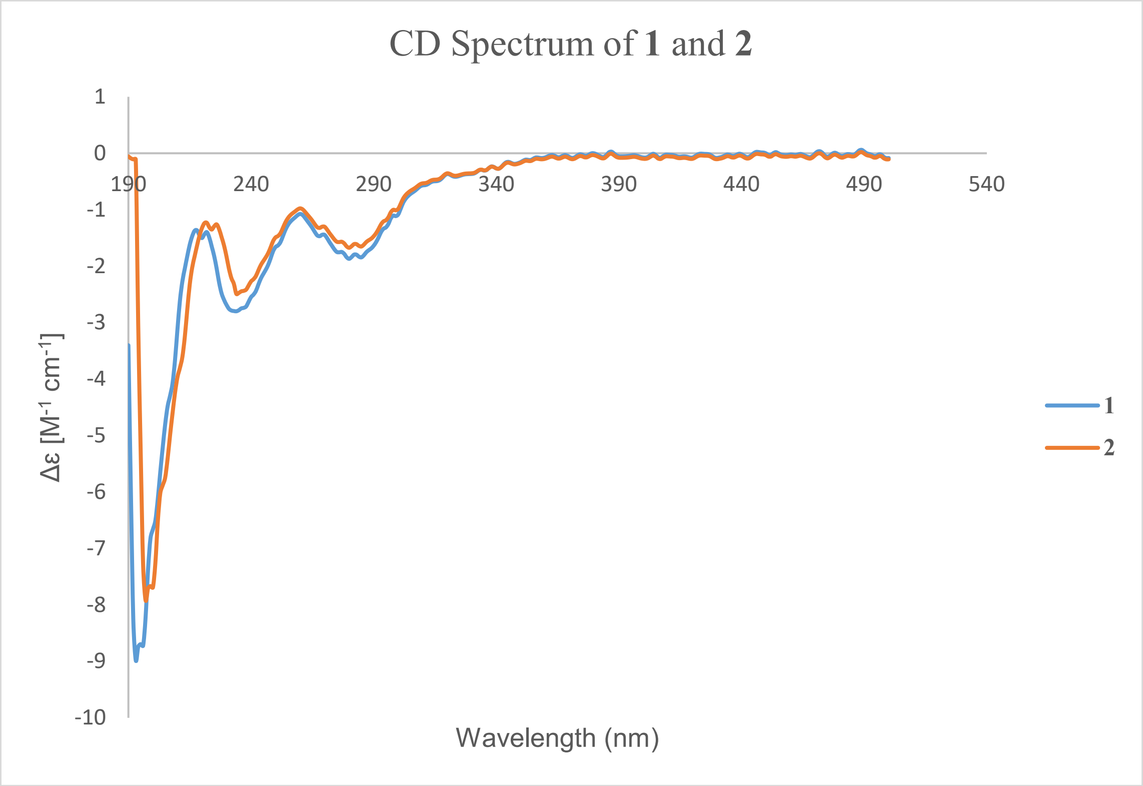

Figure S32. CD spectrum of compounds 1 and 2

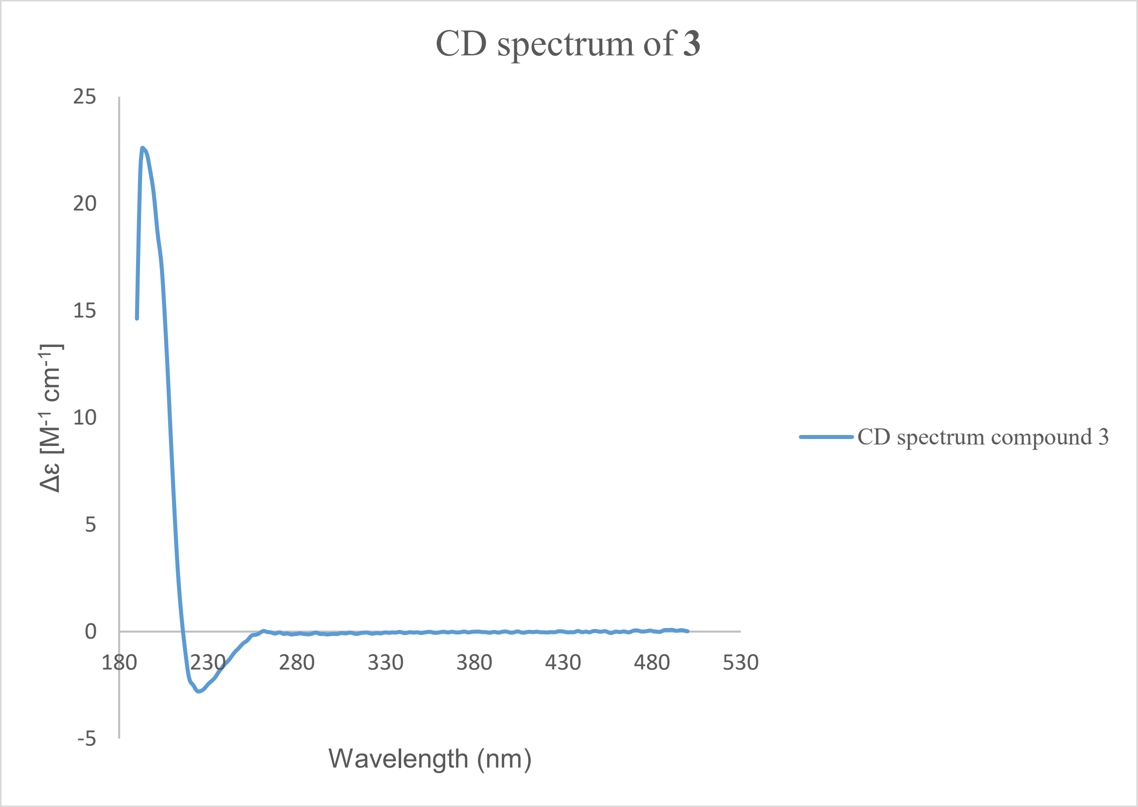

Figure S33. CD spectrum of compound 3.

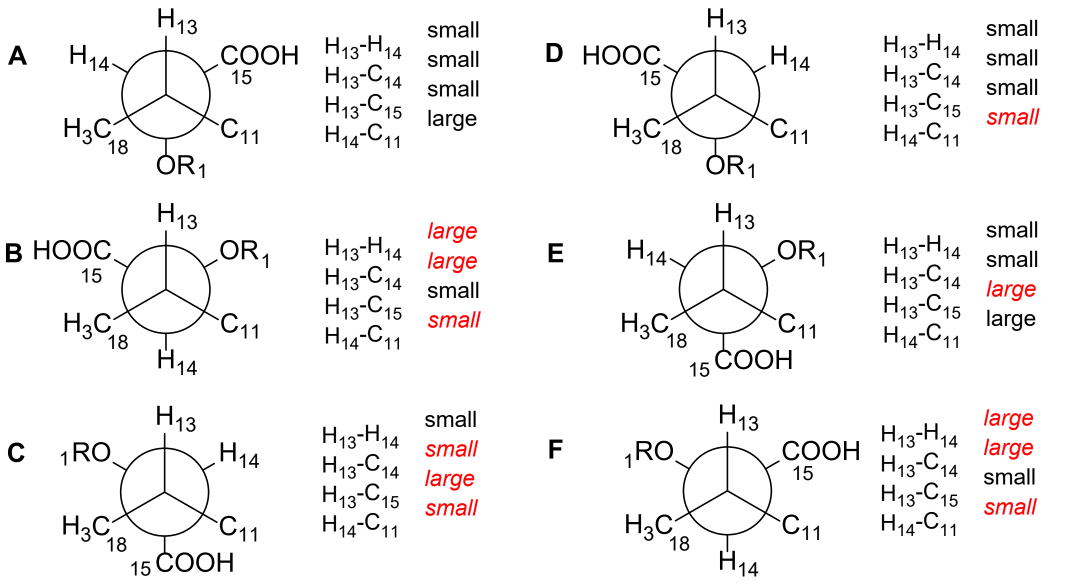

Figure S34. *J*-based configurational analysis of six hypothetical rotamers represents 13*S*, 14*S* (A-C), and 13*S*, 14*R* (D-F) configuration to determine the stereochemistry of **3**. Expected couplings contrary (shown in red) to the observed ones (^3J(H13,H14) = 2.3 Hz; ^2J(H13,C14) = 1.3 Hz; ^3J(H13,C15) = 2.1 Hz; ^3J(H14,C11) = 6.3 Hz) exclude all configurations except A.

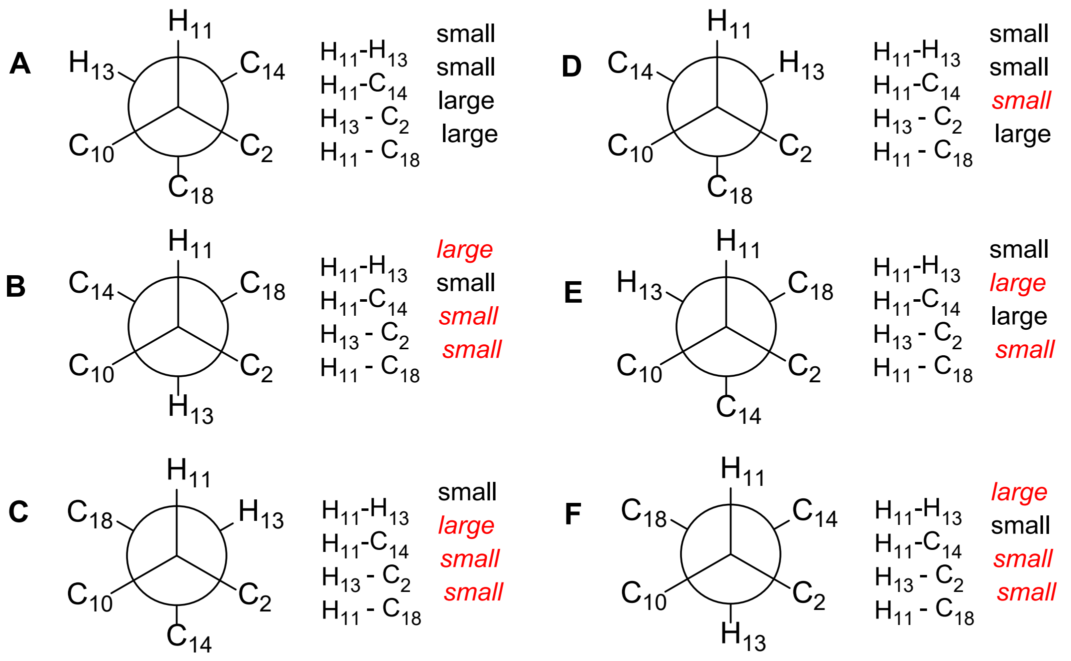

Figure S35. *J*-based configurational analysis of six hypothetical rotamers represents 11*R*, 13*S* (A-C), and 11*R*, 13*R* (D-F) configuration to determine the stereochemistry of **3**. Expected couplings contrary (shown in red) to the observed ones (^3J(H11,H13) = 4.0 Hz; ^3J(H11,C14) = 1.2 Hz; ^3J(H13,C2) = 6.7 Hz) exclude all configurations except A.

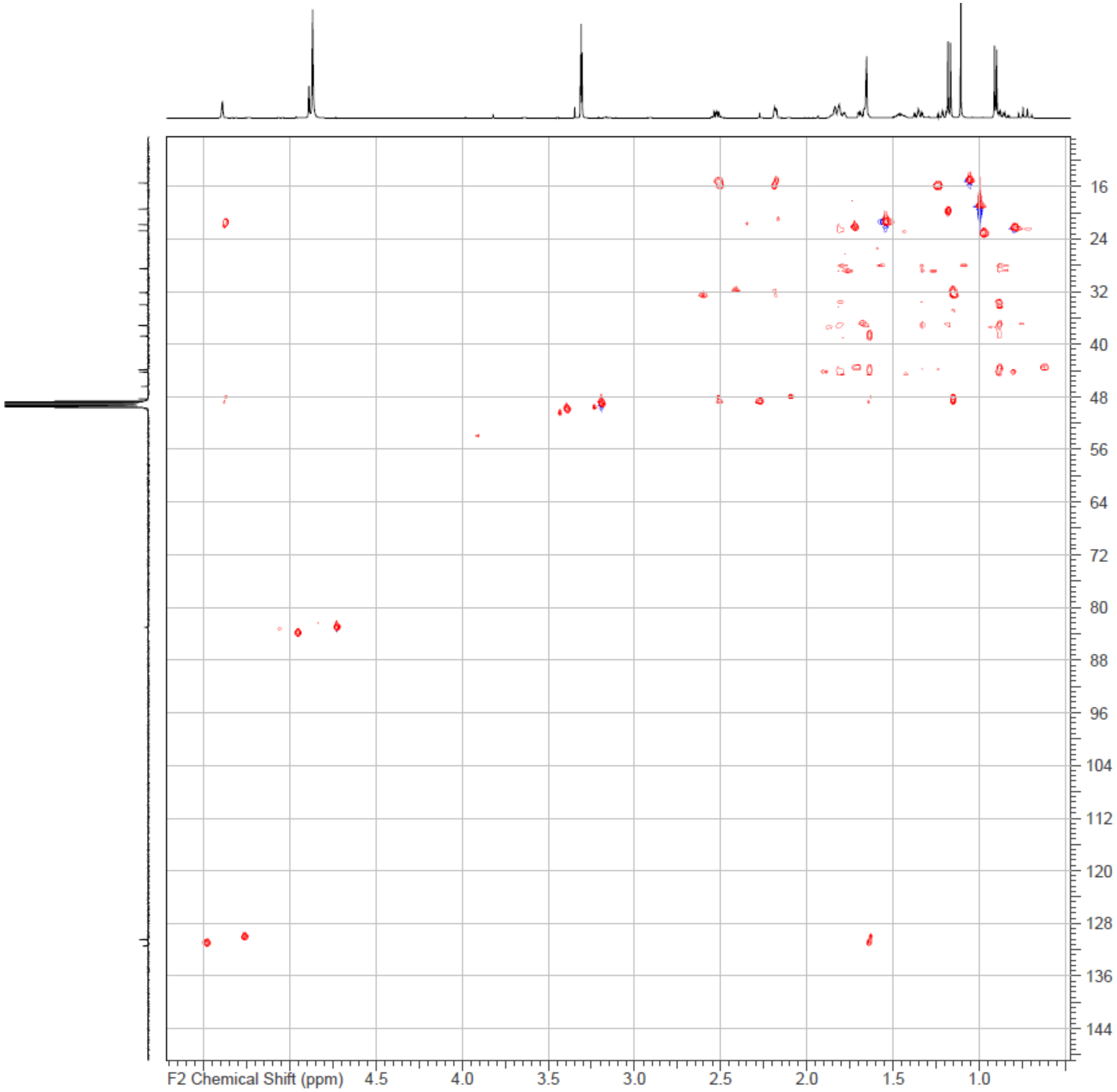

Figure S36. ^{1}H, ^{13}C HSQC-Hecade NMR spectrum of colposetin C in CD_3OD (700 MHz)

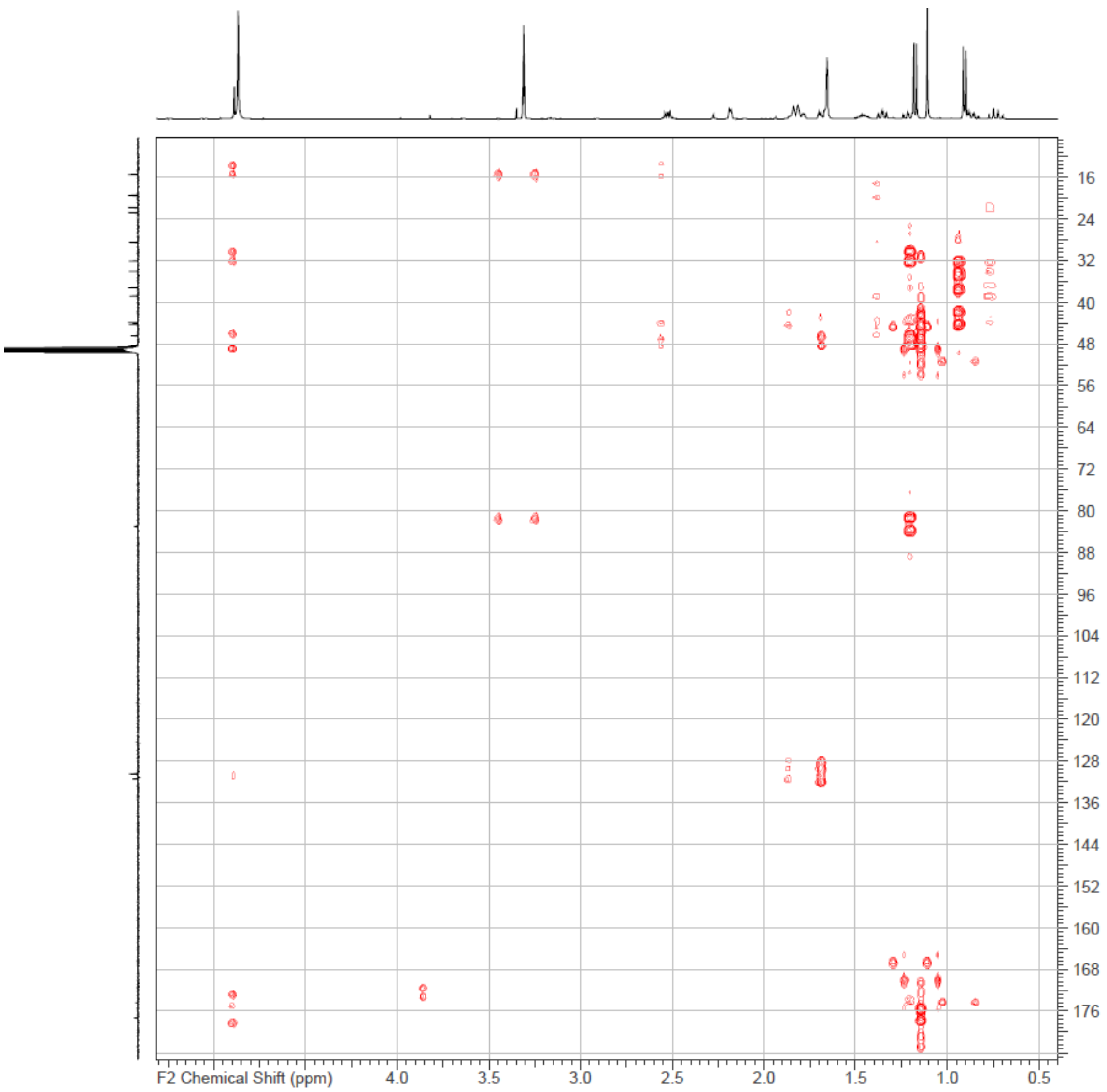

Figure S37. ^{1}H, ^{13}C *J*-HMBC NMR spectrum of colposetin C in CD_3OD (700 MHz)

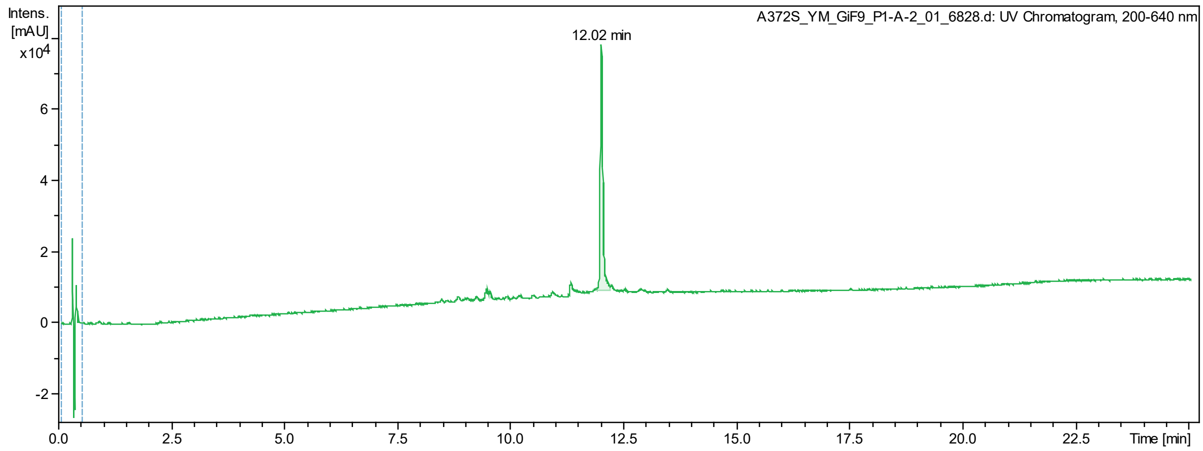

Figure S38. HPLC-DAD/MS chromatogram of colpomenoic acid A

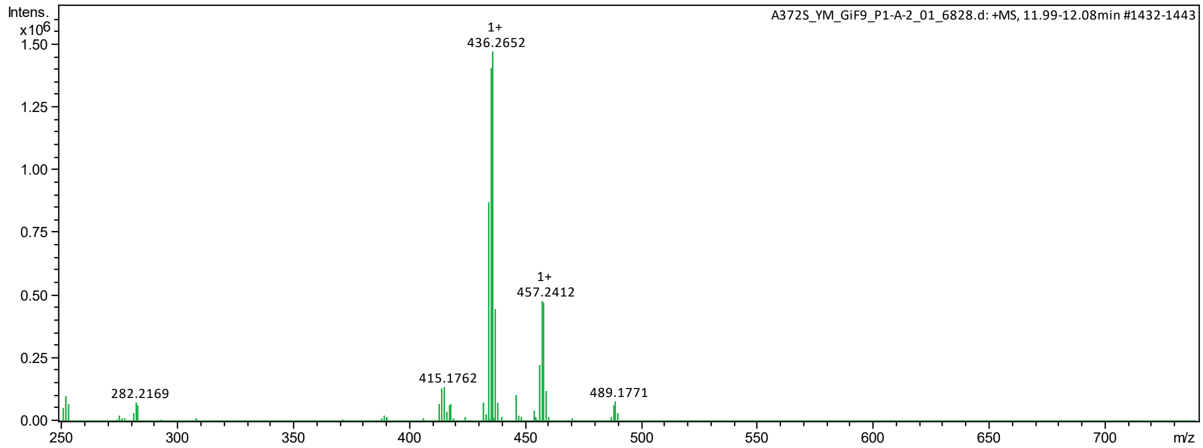

Figure S39. HR-ESIMS chromatogram of colpomenoic acid A

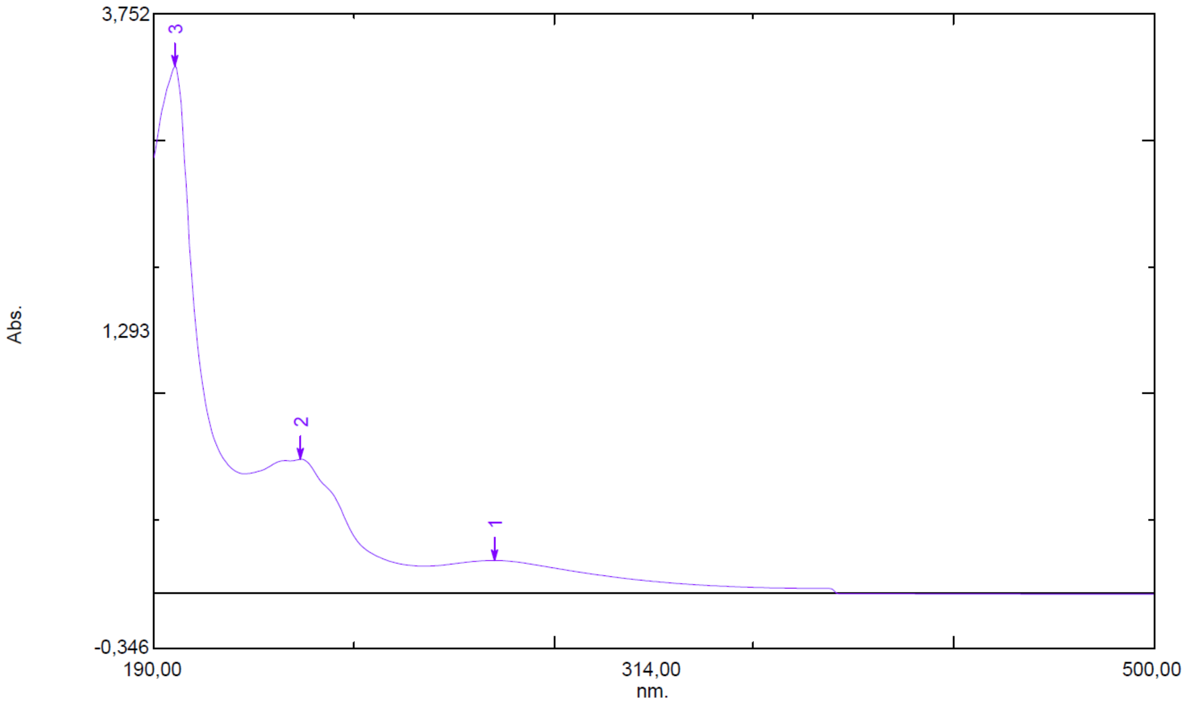

Figure S40. UV/vis spectrum of colpomenoic acid A in MeOH (log ε) [neutral]) λ_{max} (log ε) 235 (4.57), 295 (3.92) nm.

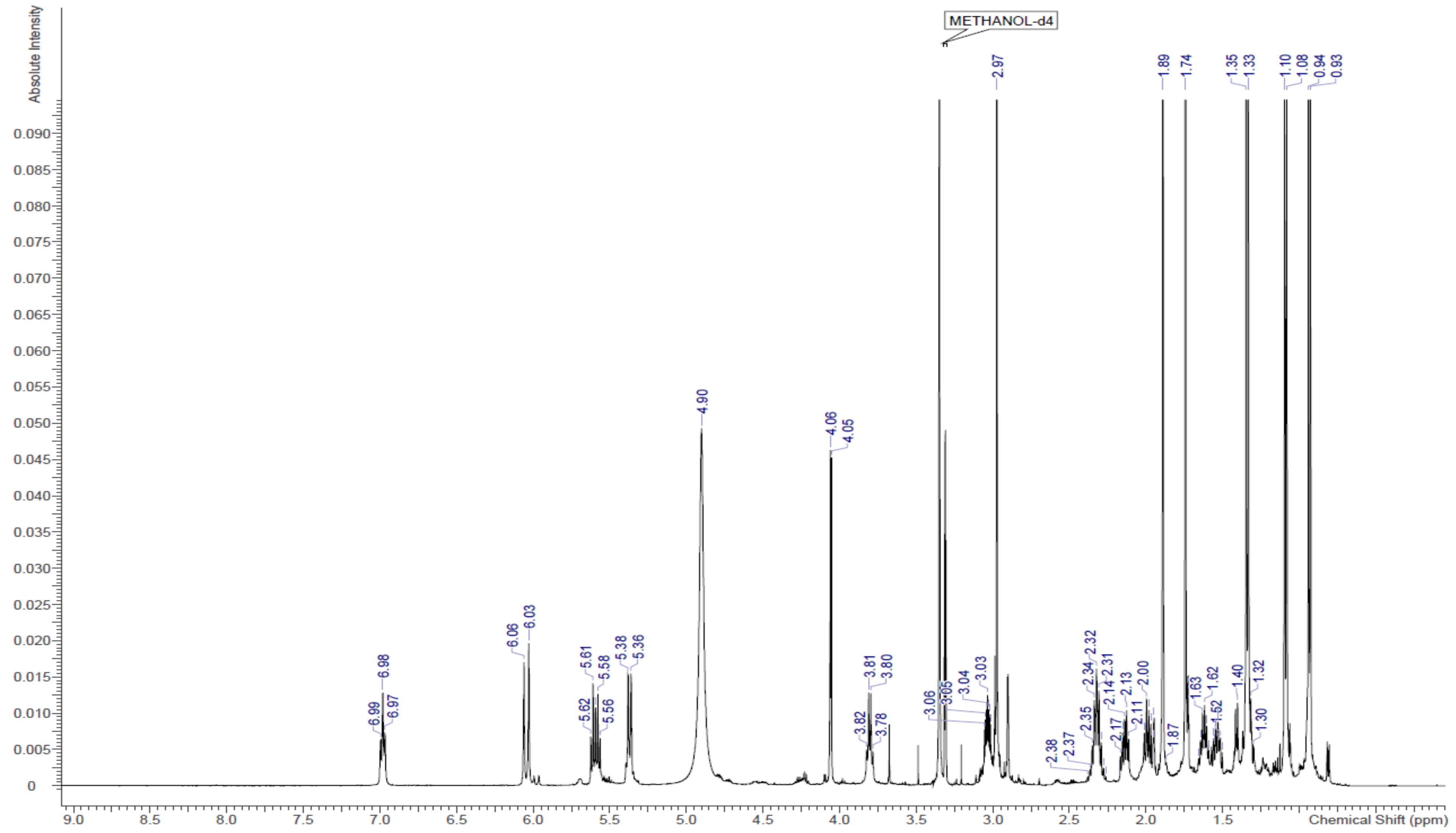

Figure S41. ^{1}H NMR spectrum of colpomenoic acid A in CD_3OD (700 MHz)

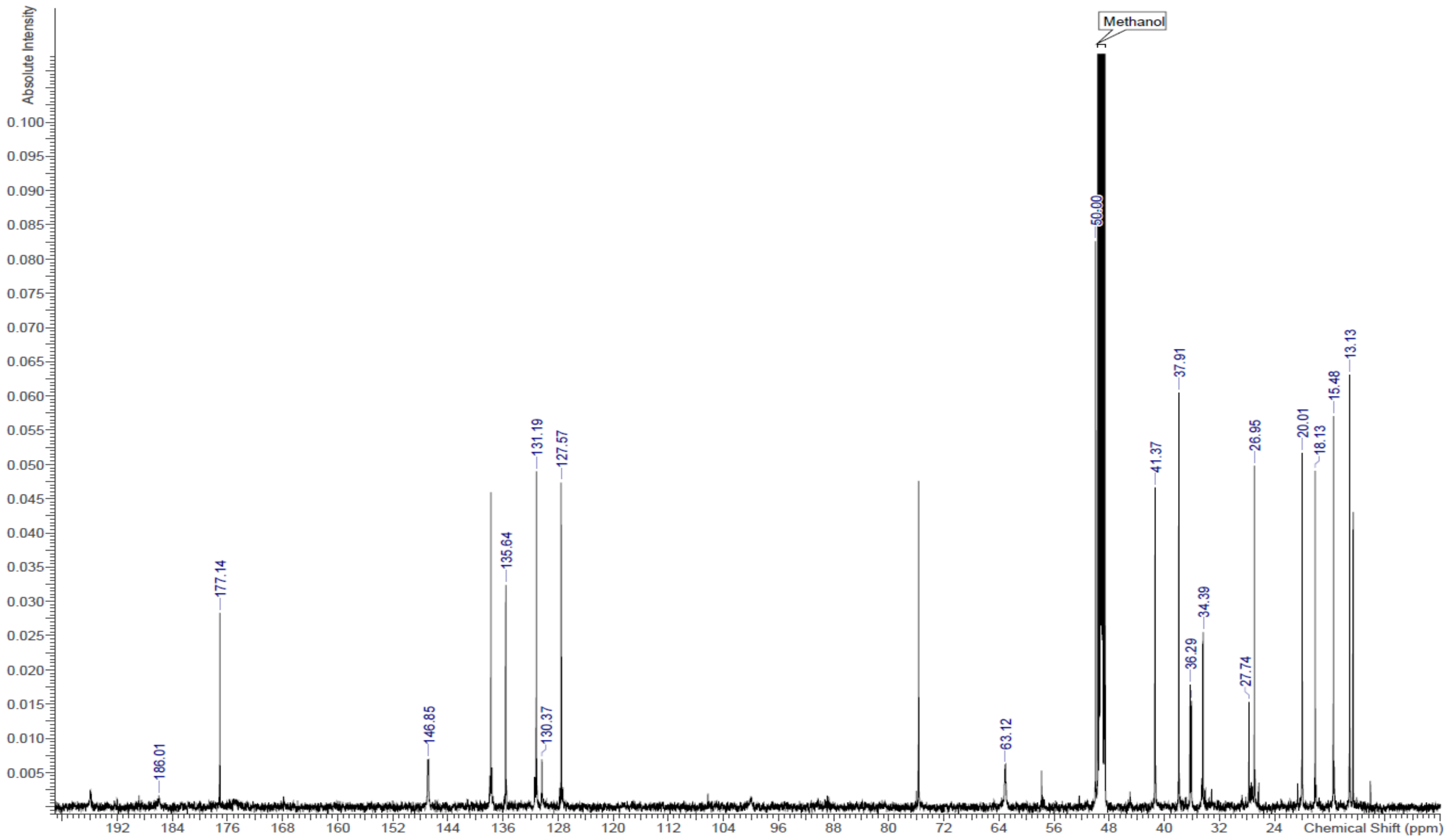

Figure S42. ^{13}C NMR spectrum of colpomenoic acid A in CD_3OD (700 MHz)

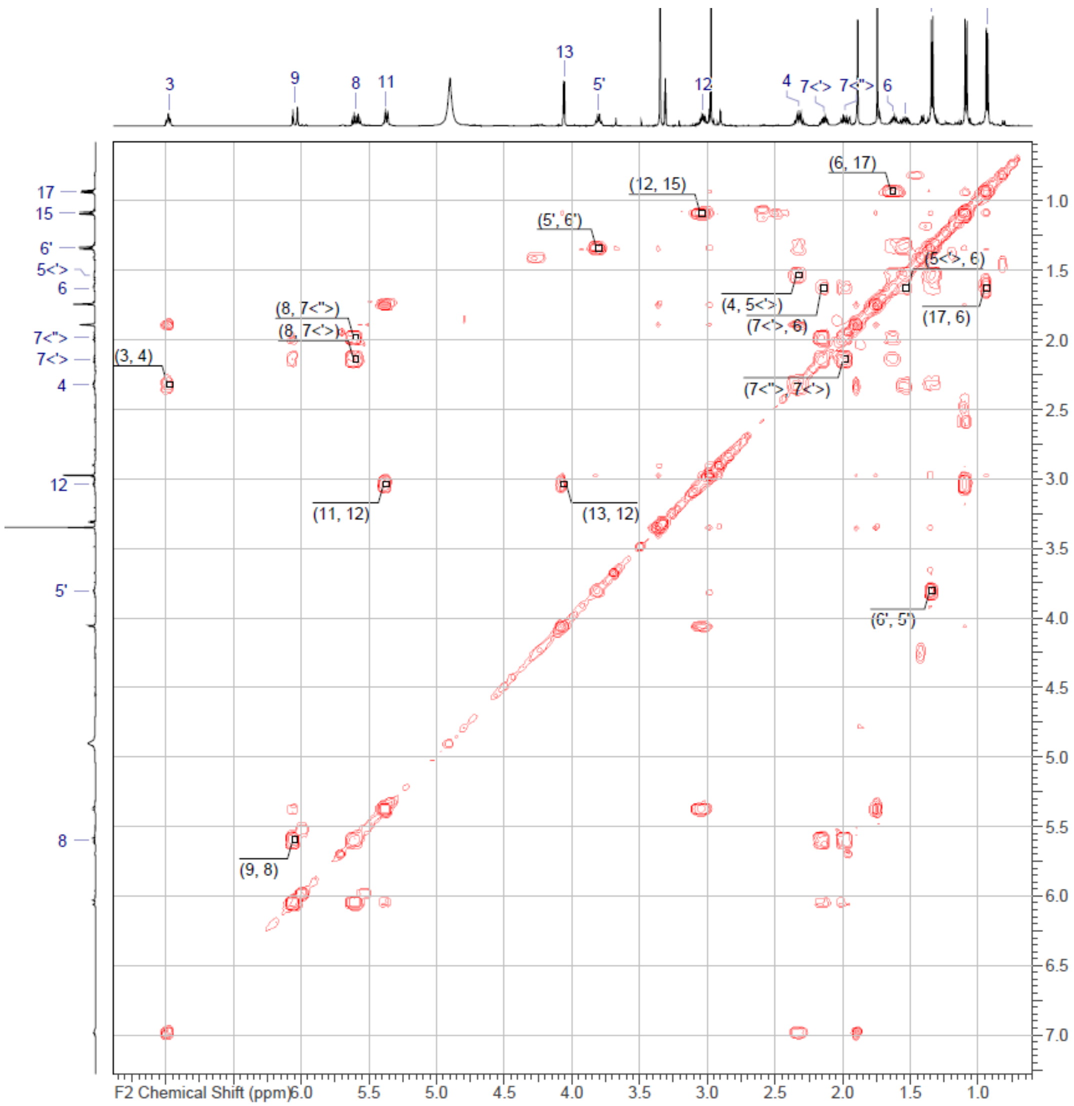

Figure S43. ^{1}H,^{1}H COSY NMR spectrum of colpomenoic acid A in CD_3OD (700 MHz)

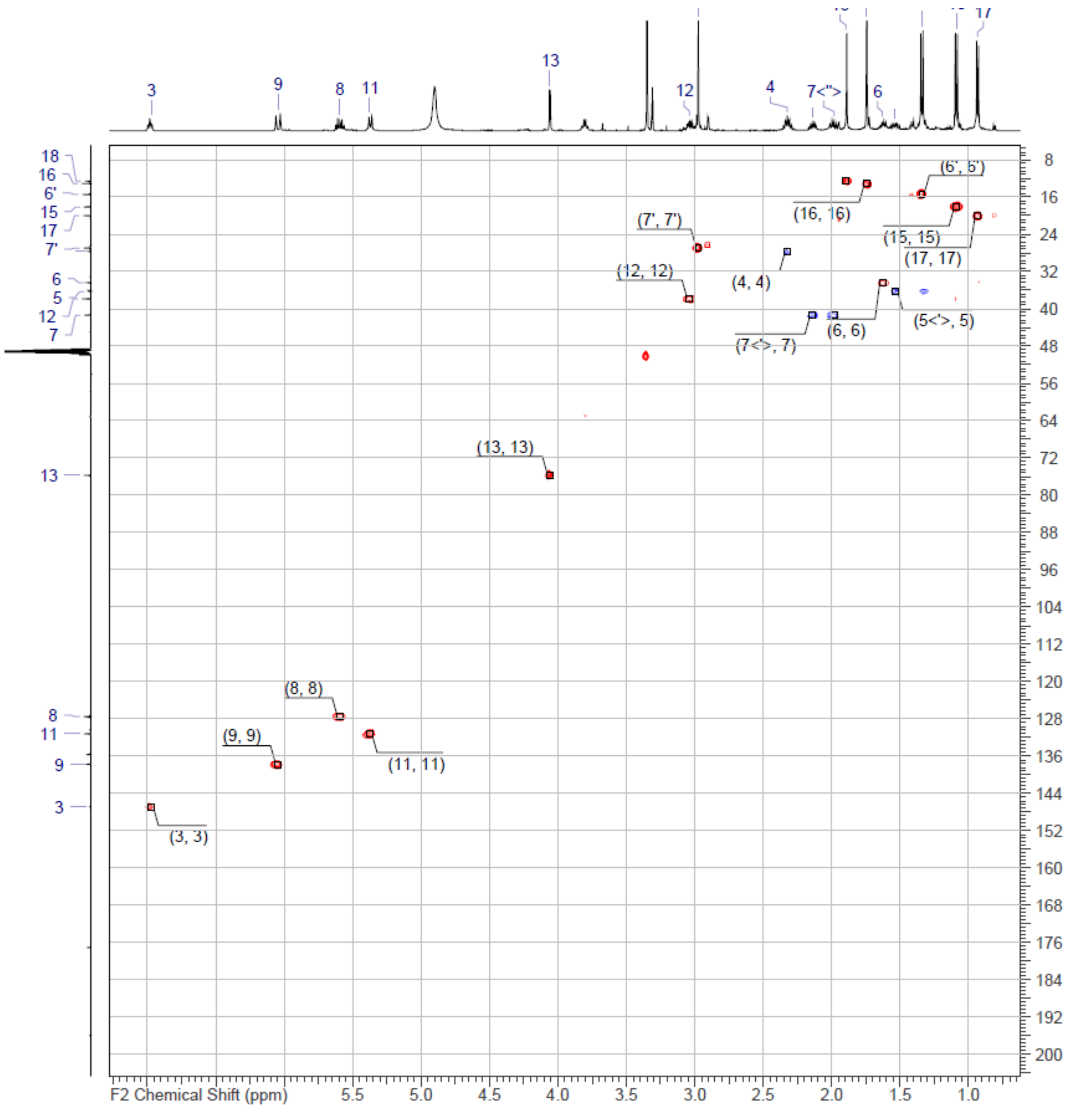

Figure S44. $^{1}H,^{13}C$ HSQC-DEPT NMR spectrum of colpomenoic acid A in CD_3OD (700 MHz, 176 MHz)

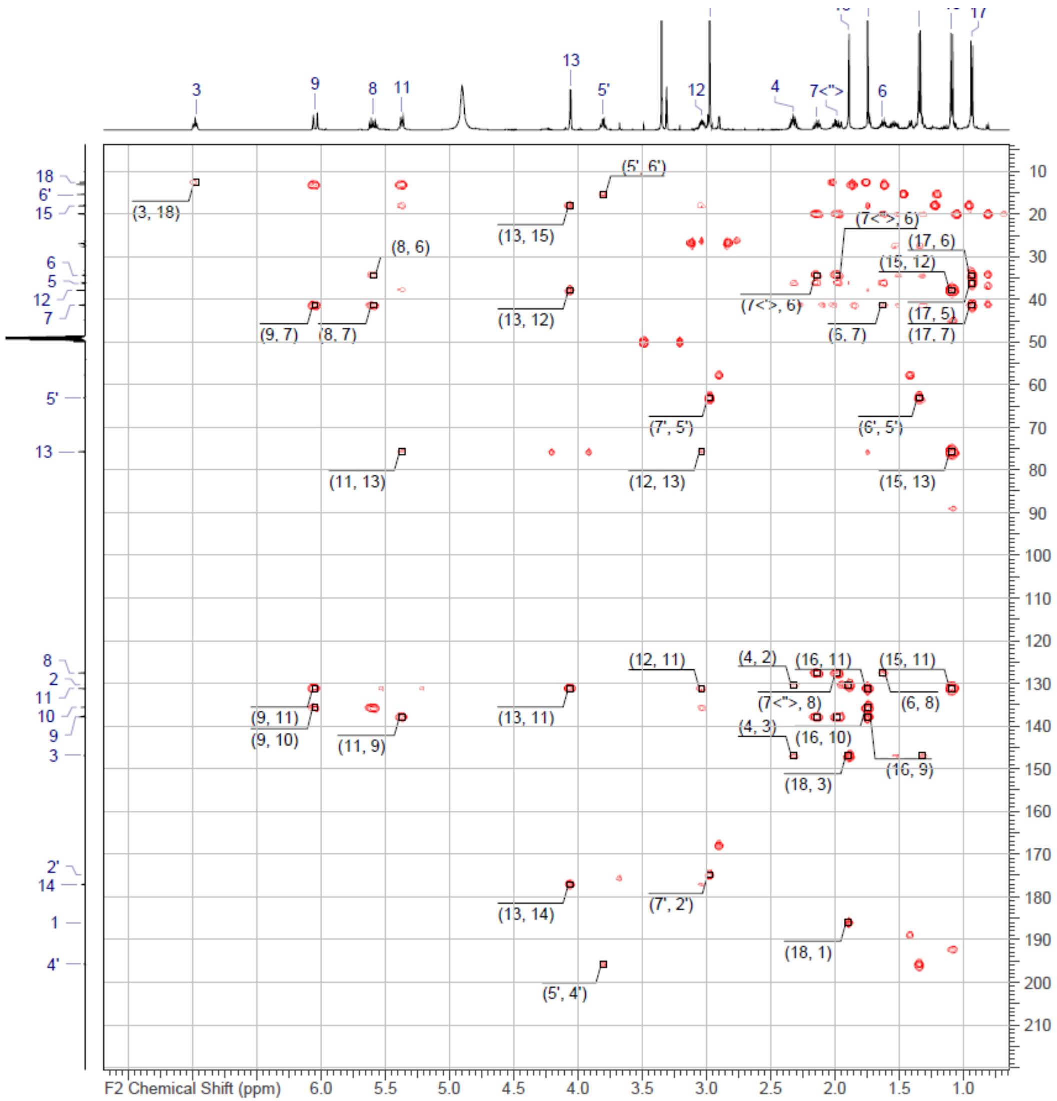

Figure S45. $^{1}H,^{13}C$ HMBC NMR spectrum of colpomenoic acid A in CD_3OD (700 MHz, 176 MHz)

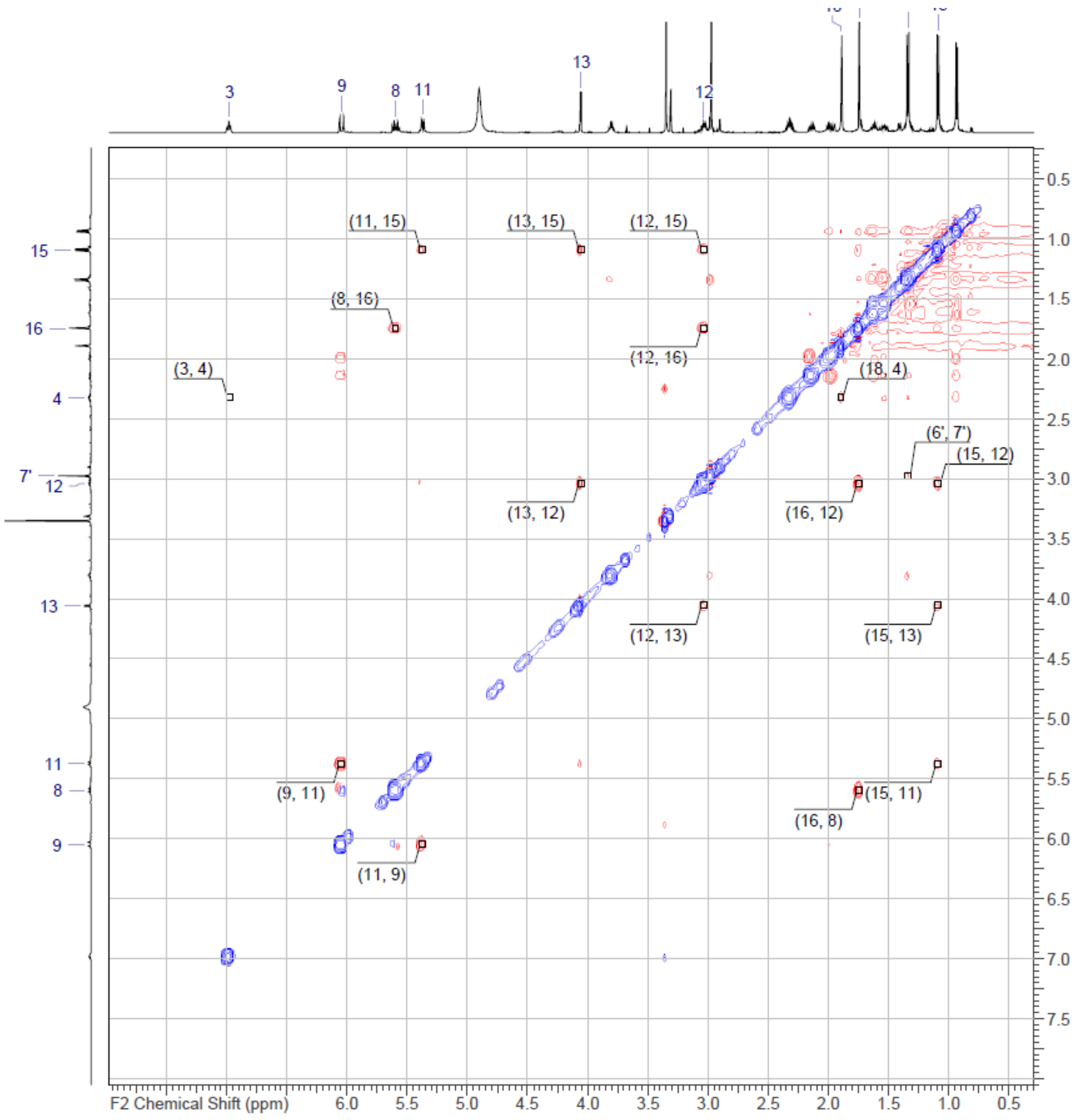

Figure S46. ROESY NMR spectrum of colpomenoic acid A in CD_3OD (700 MHz)

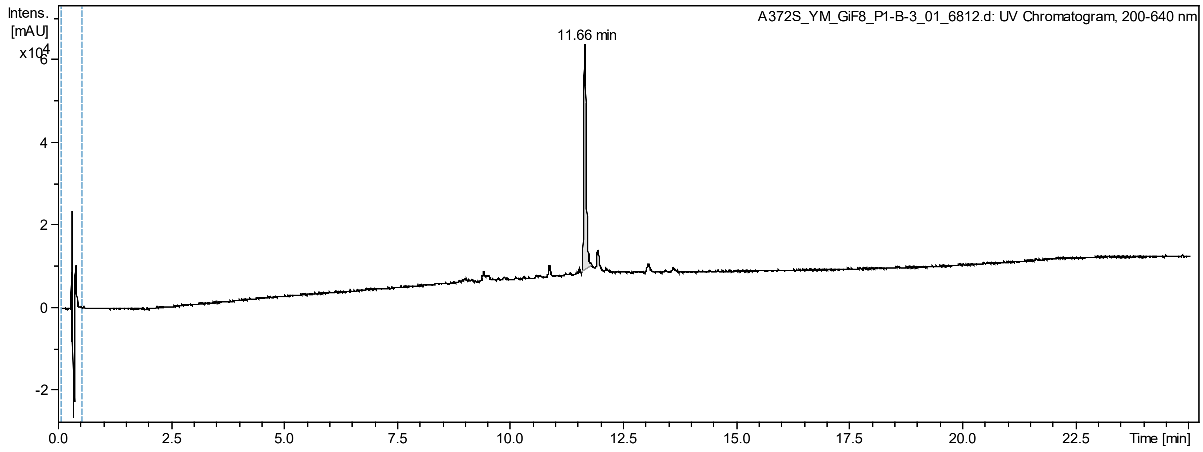

Figure S47. HPLC-DAD/MS chromatogram of colpomenoic acid B

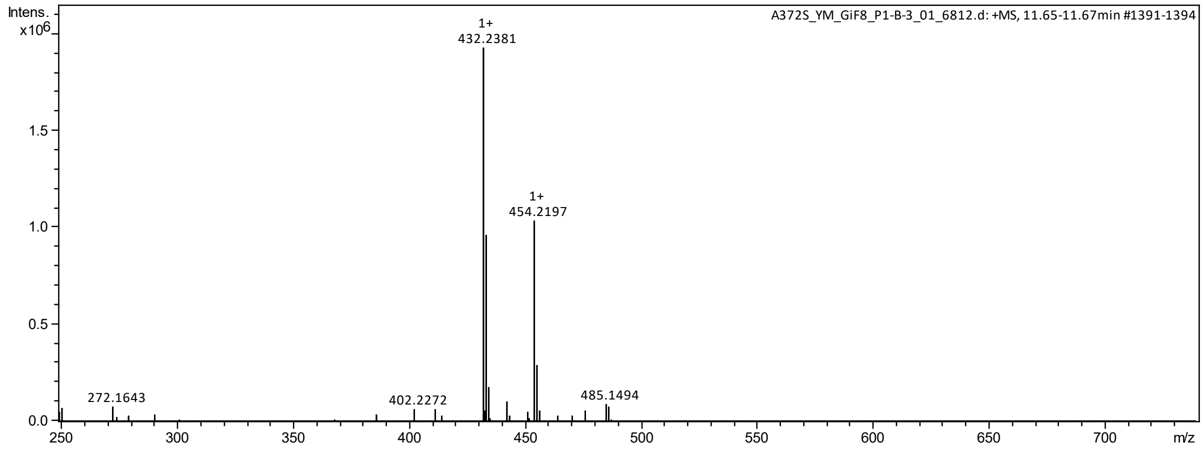

Figure S48. HR-ESIMS chromatogram of colpomenoic acid B

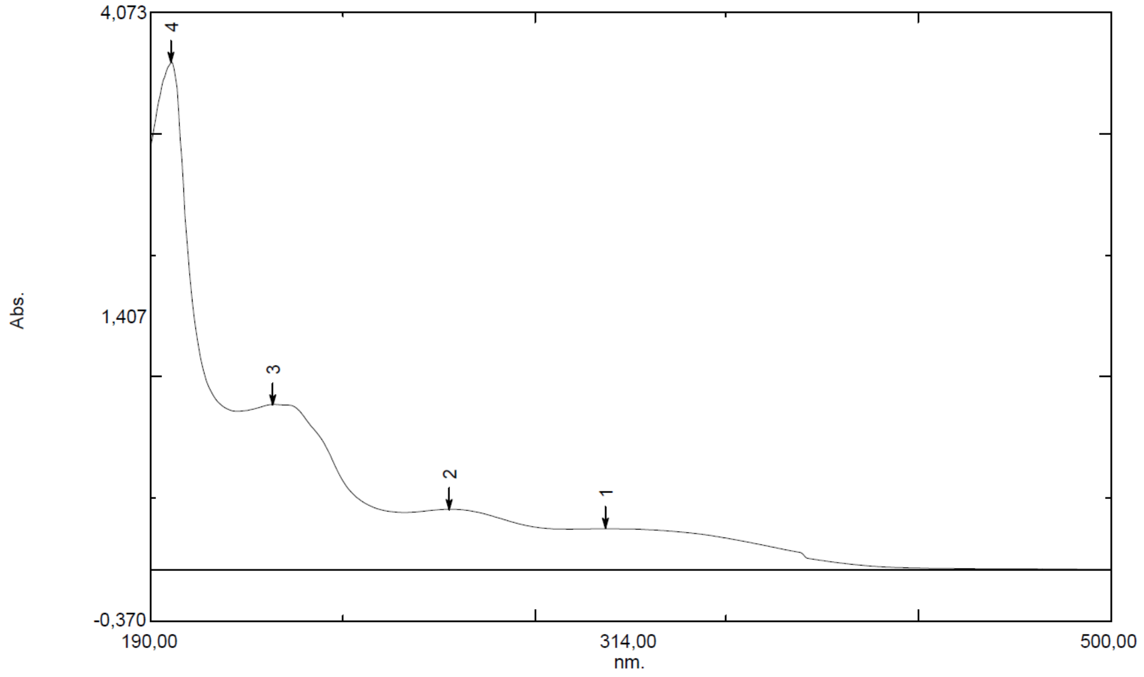

Figure S49. UV/vis spectrum of colpomenoic acid B in MeOH (log ε) [neutral]) λ_{max} (log ε) 229 (4.41), 286 (3.97) nm.

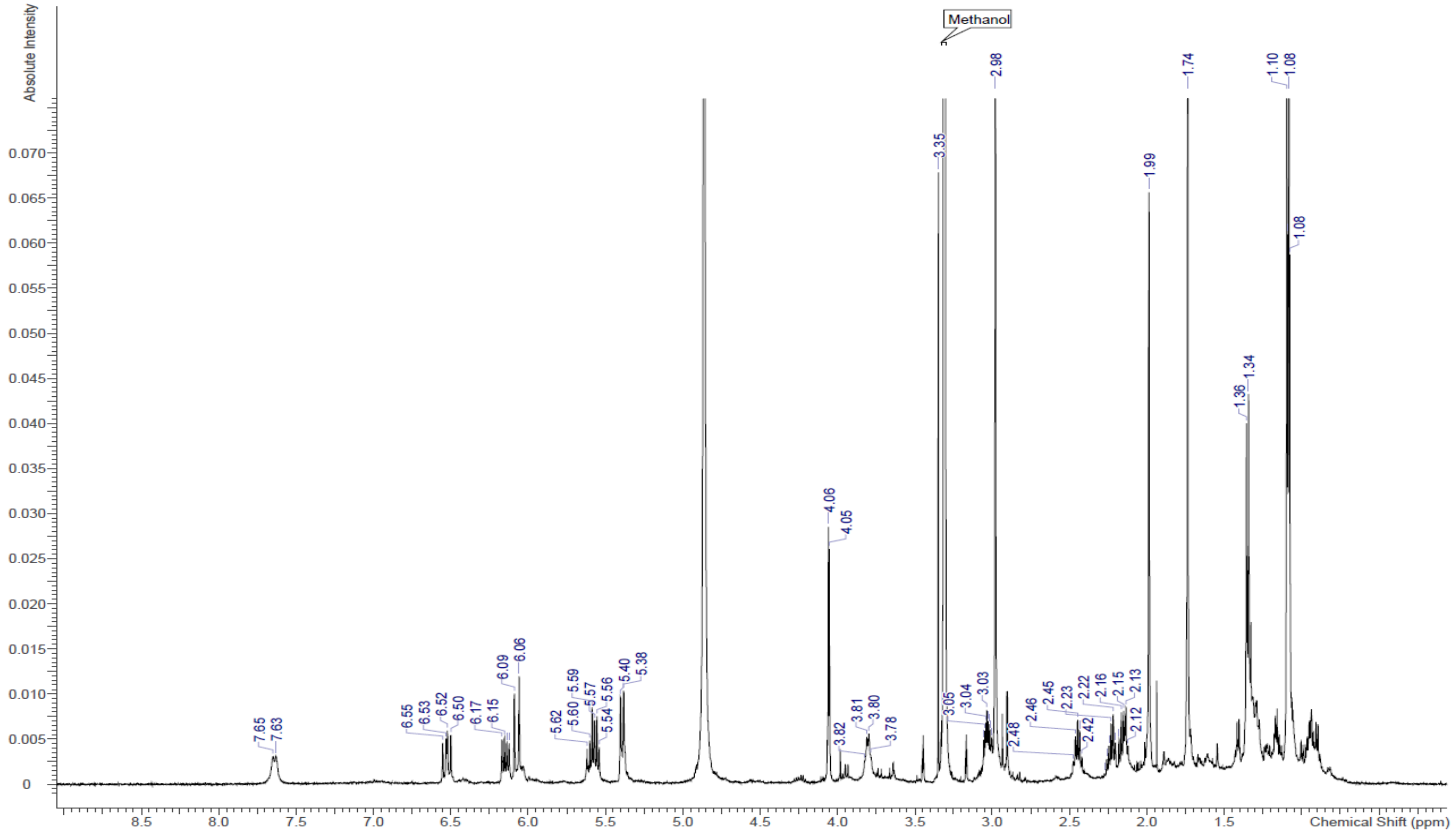

Figure S50. ^{1}H NMR spectrum of colpomenoic acid B in CD_3OD (700 MHz)

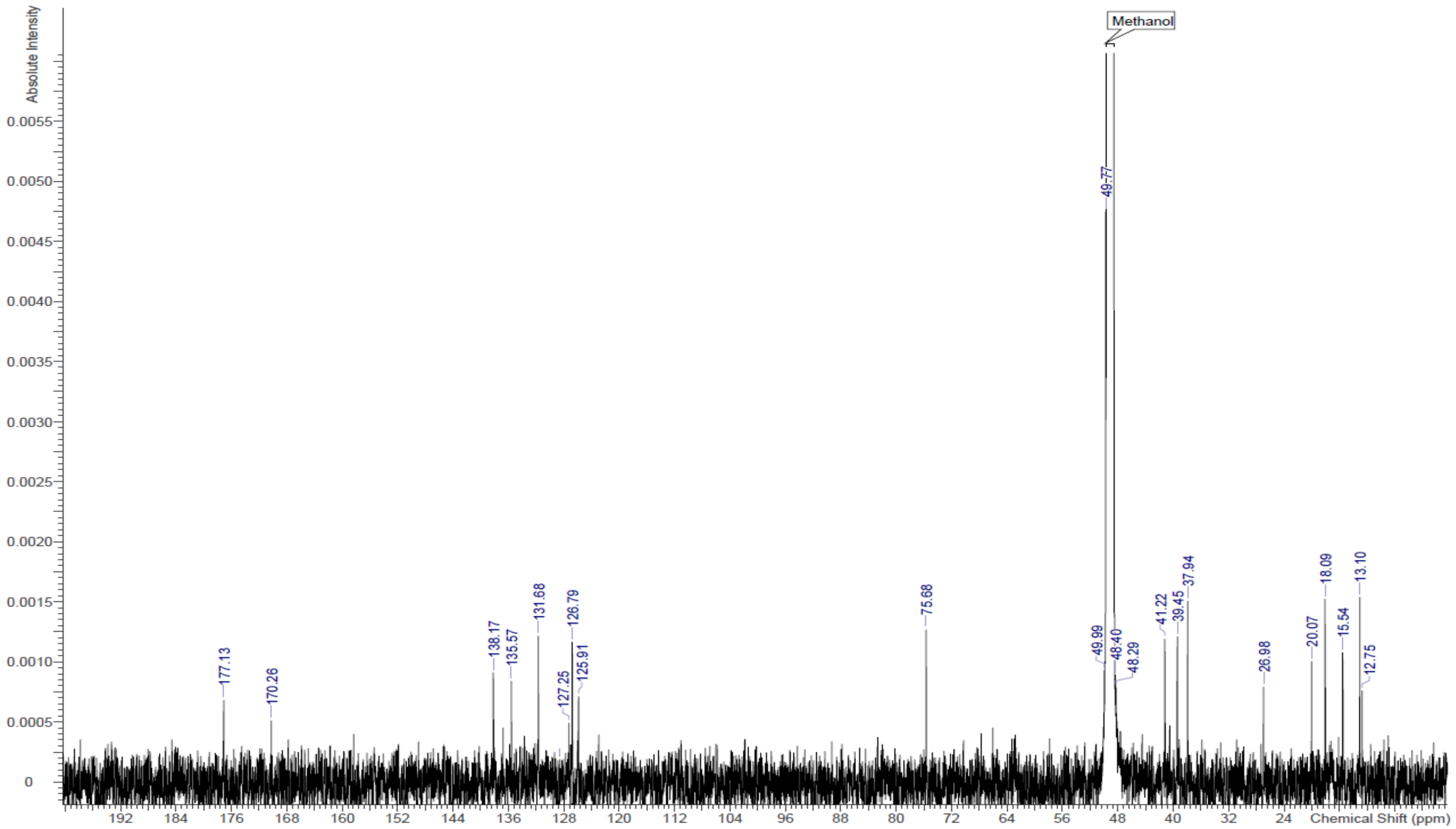

Figure S51. ^{13}C NMR spectrum of colpomenoic acid B in CD_3OD (700 MHz)

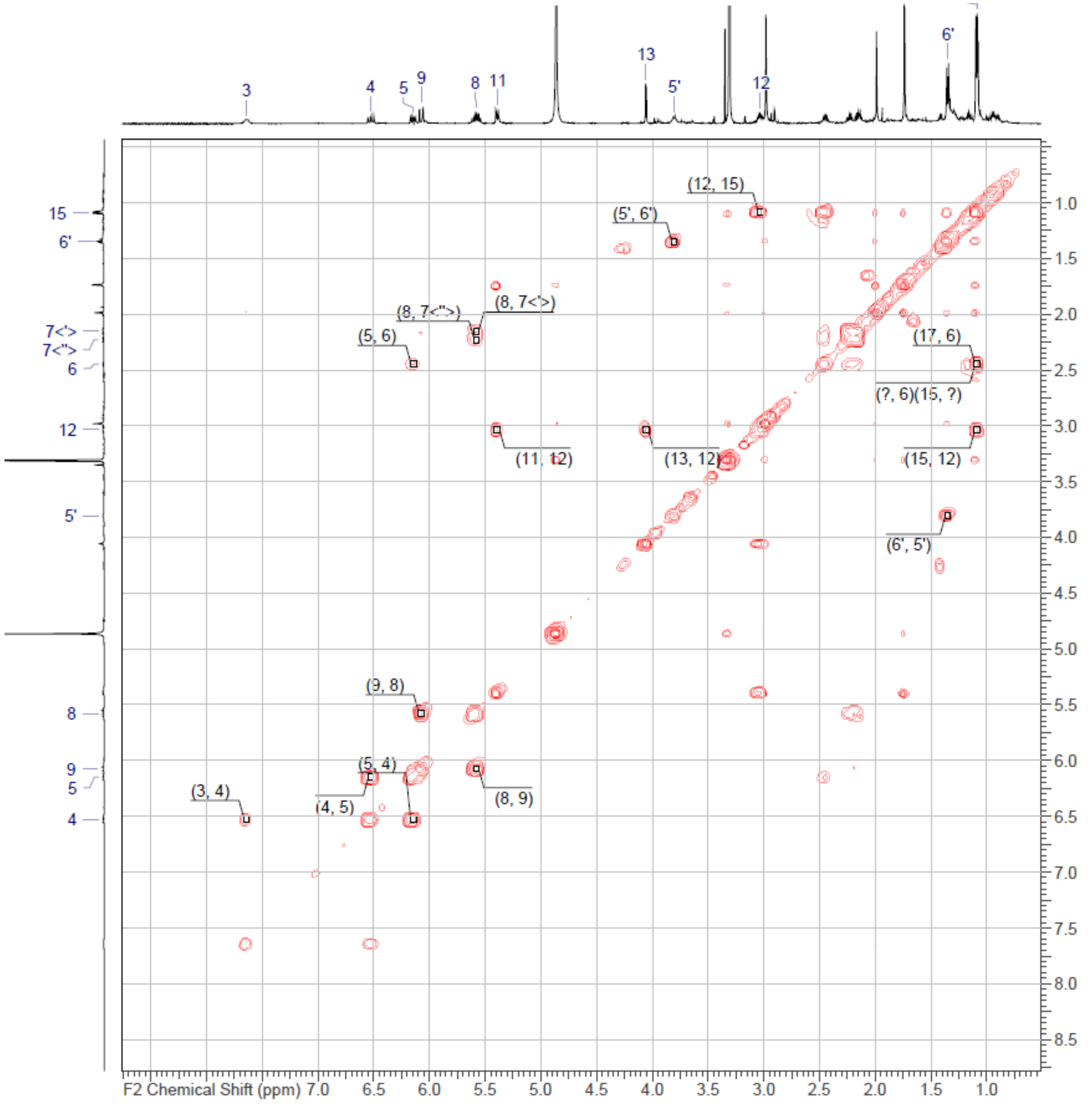

Figure S52. $^{1}H,^{1}H$ COSY NMR spectrum of colpomenoic acid B in CD_3OD (700 MHz)

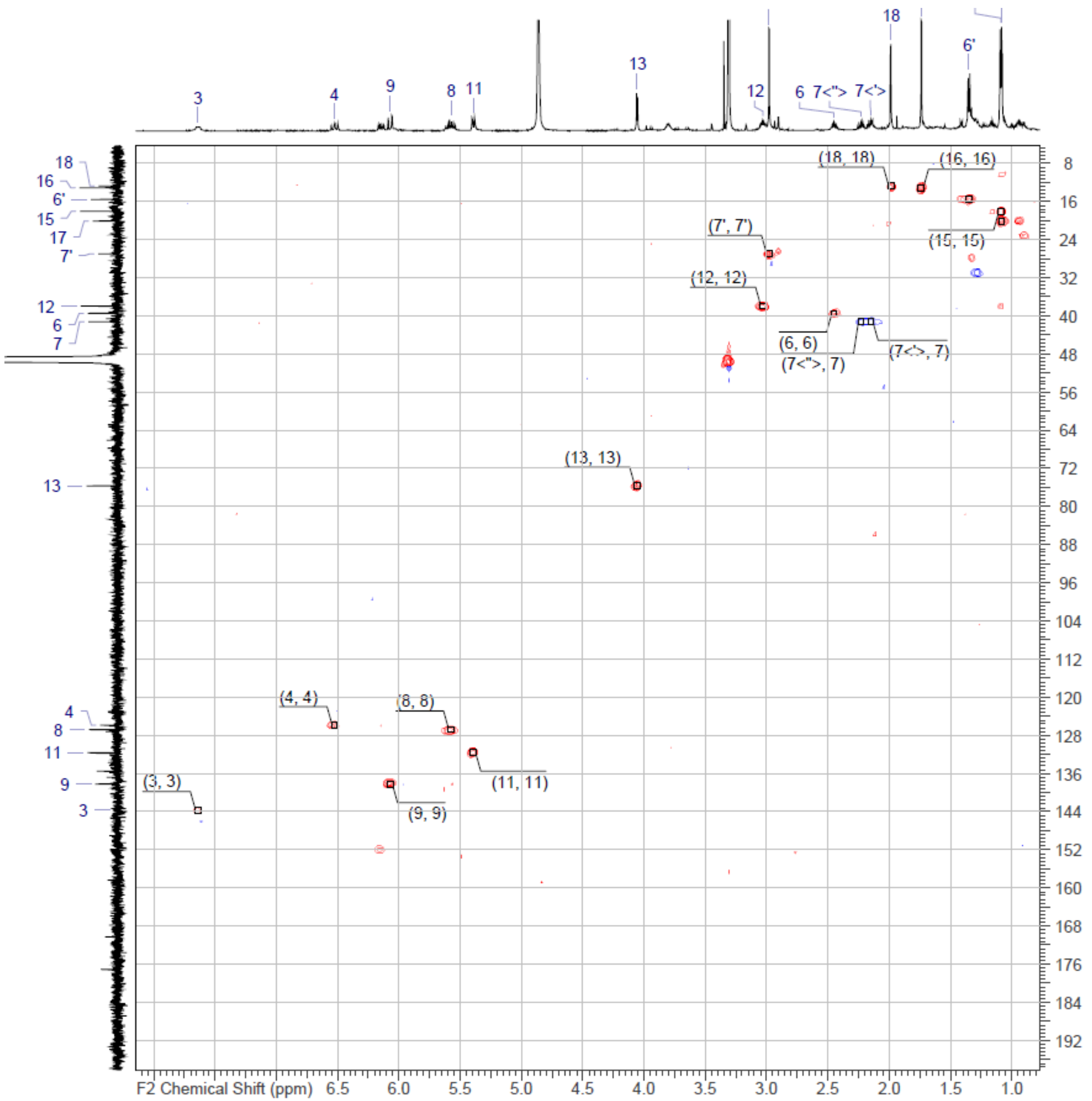

Figure S53. ^{1}H,^{13}C HSQC-DEPT NMR spectrum of colpomenoic acid B in CD_3OD (700 MHz, 176 MHz)

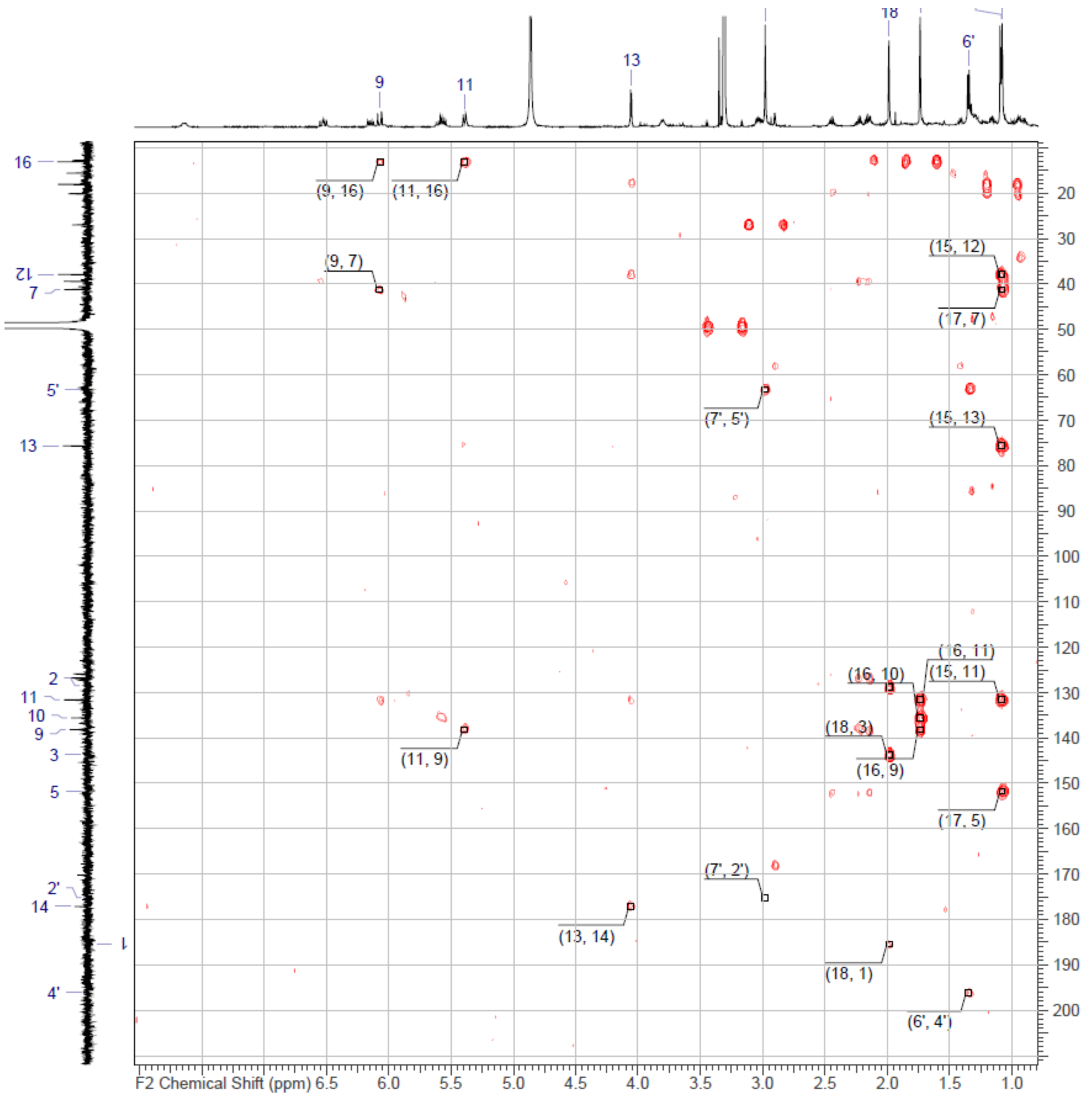

Figure S54. ^{1}H,^{13}C HMBC NMR spectrum of colpomenoic acid B in CD_3OD (700 MHz, 176 MHz)

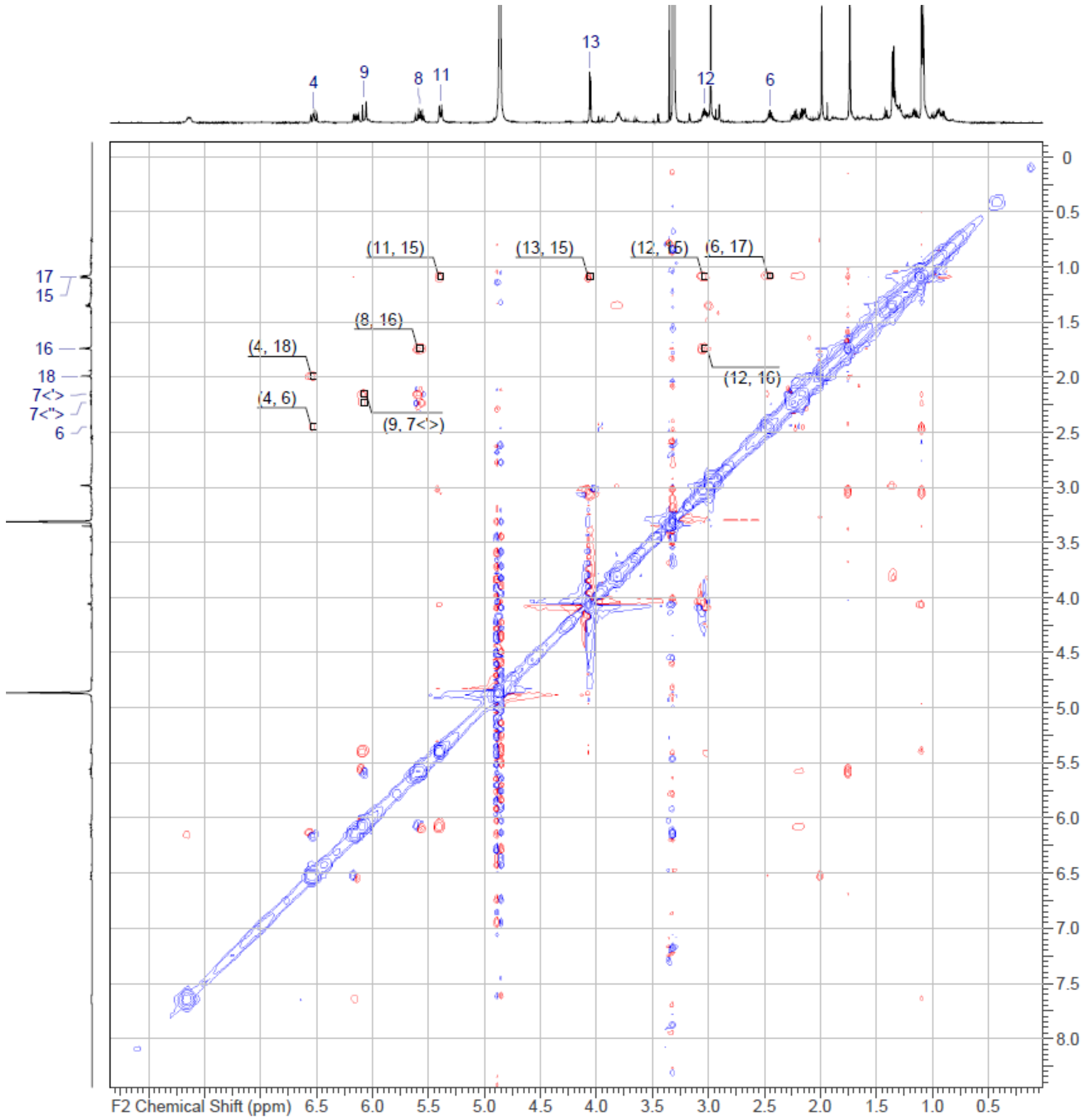

Figure S55. ROESY NMR spectrum of colpomenoic acid B in CD_3OD (700 MHz)

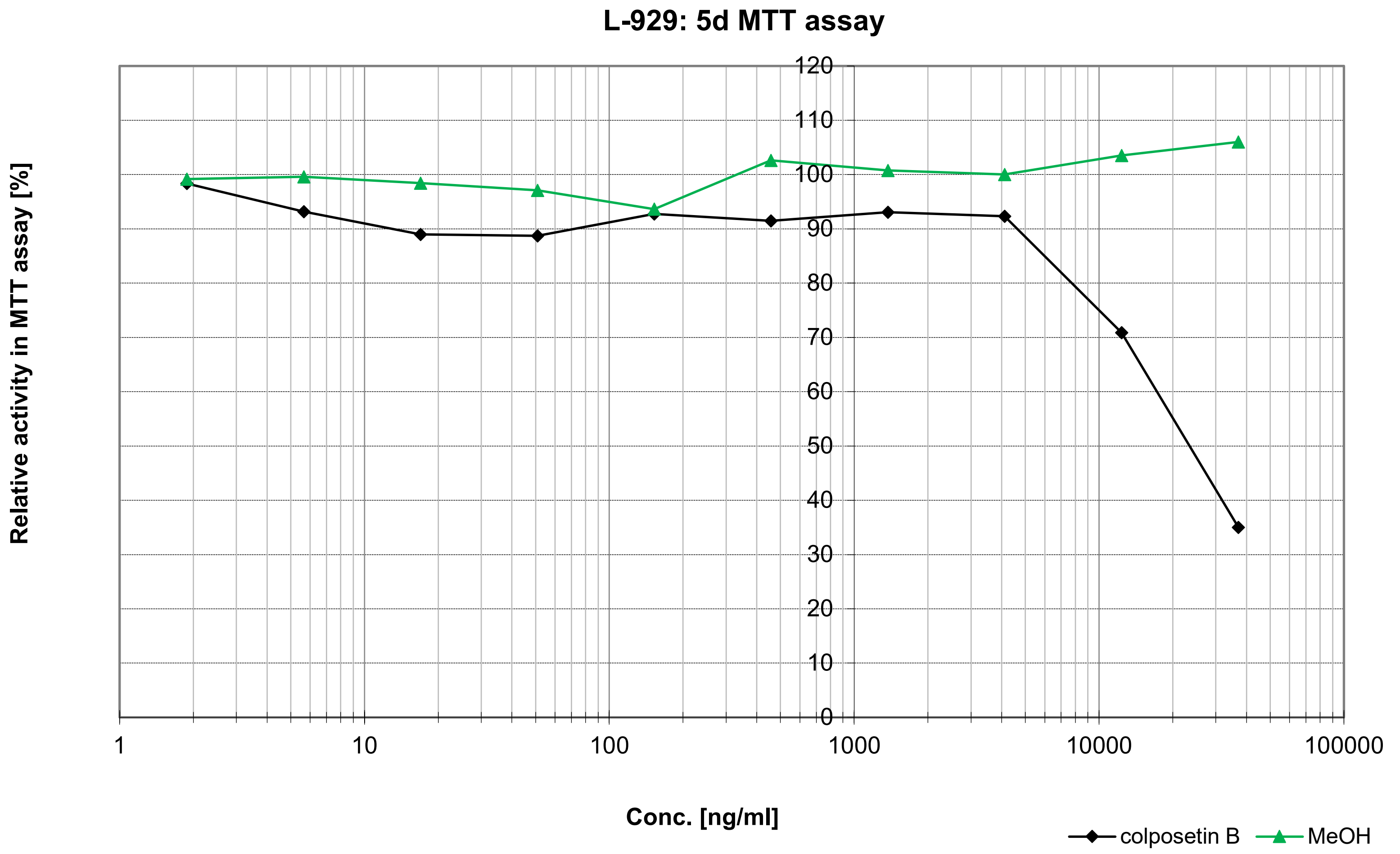

Figure S56. Graph for calculating IC_{50} of colposetin B against mouse fibroblasts L-929

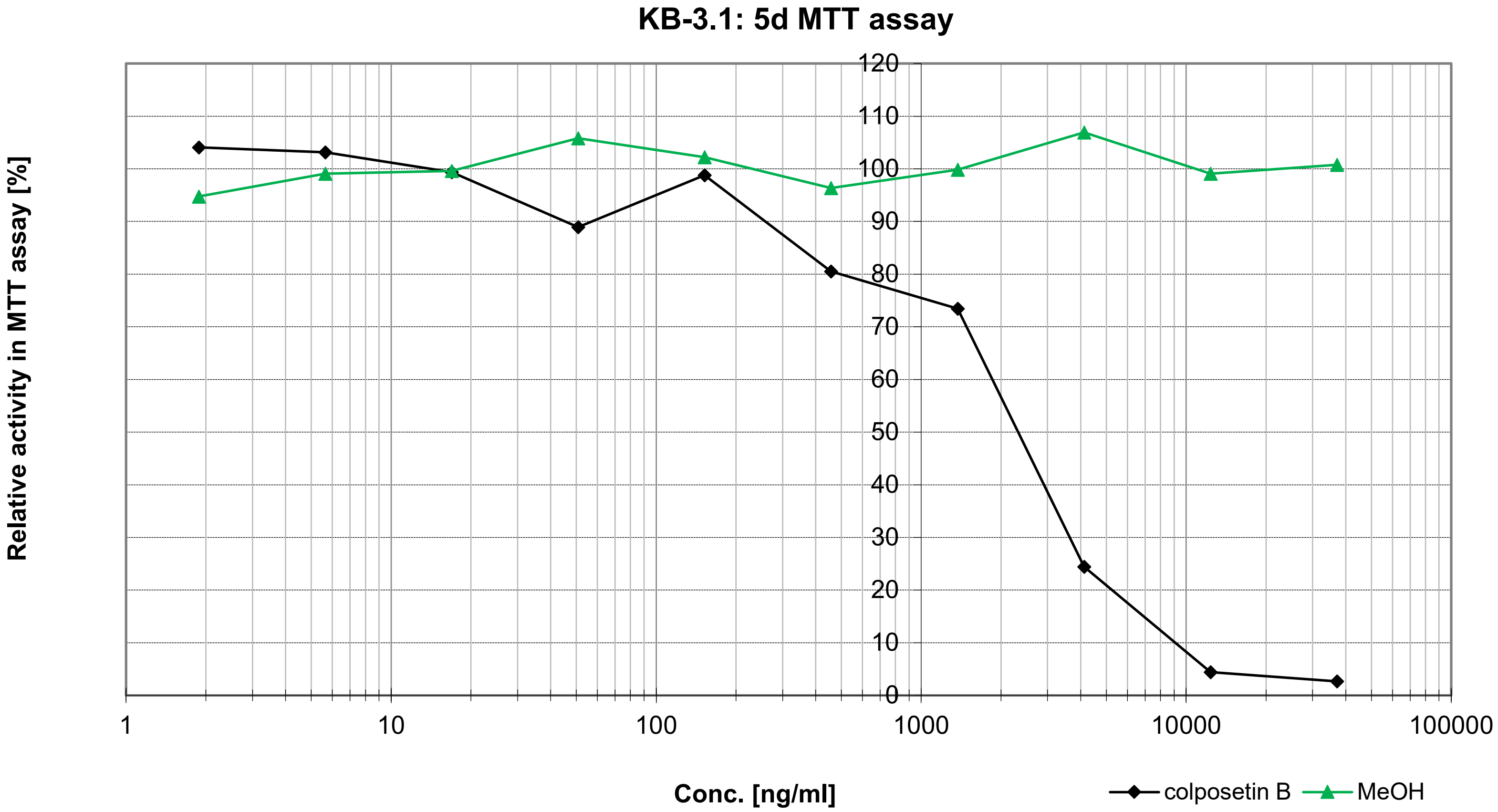

Figure S57. Graph for calculating IC_{50} of colposetin B against human endocervical adenocarcinoma KB-3.1

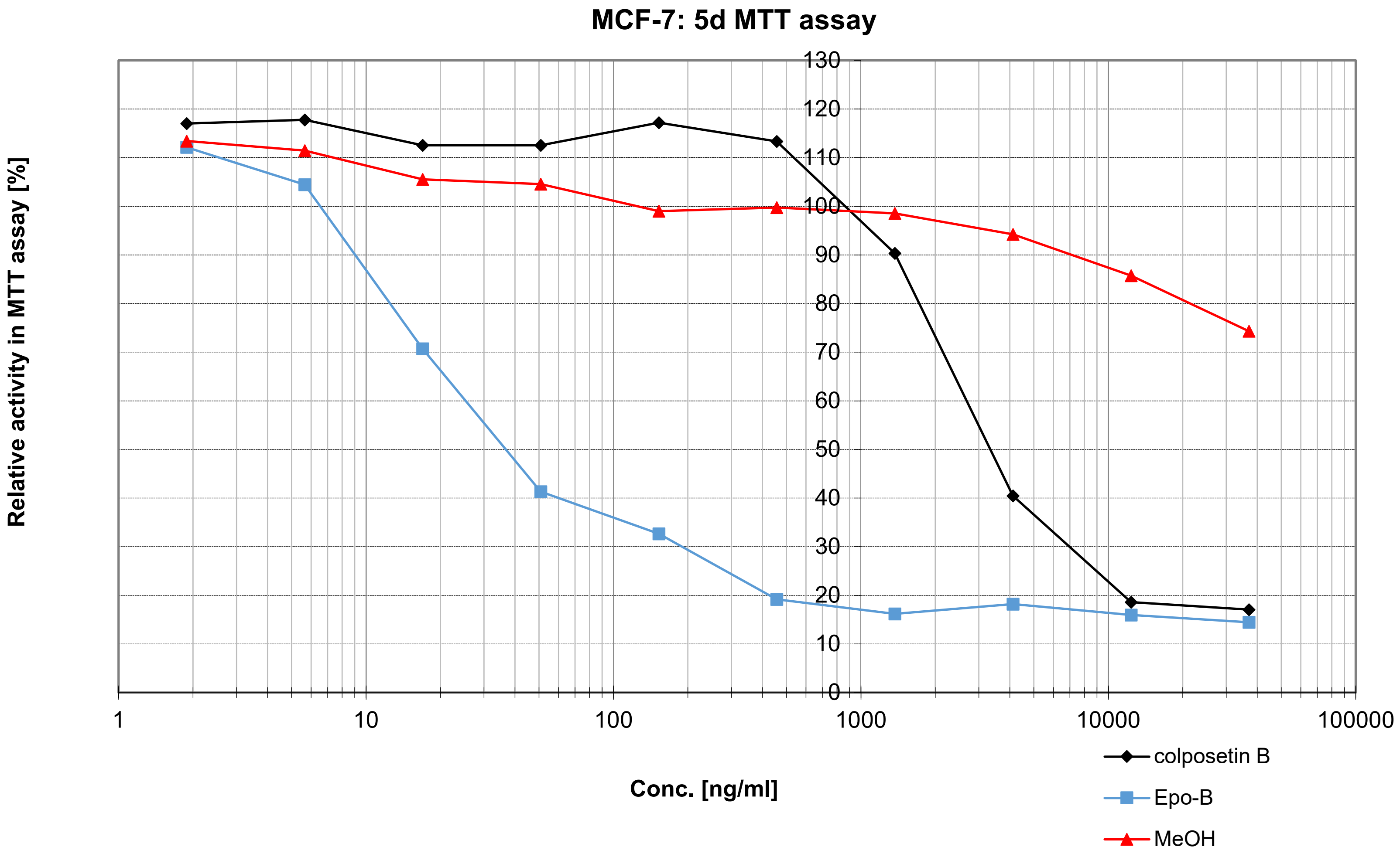

Figure S58. Graph for calculating IC_{50} of colposetin B against human breast adenocarcinoma MCF-7

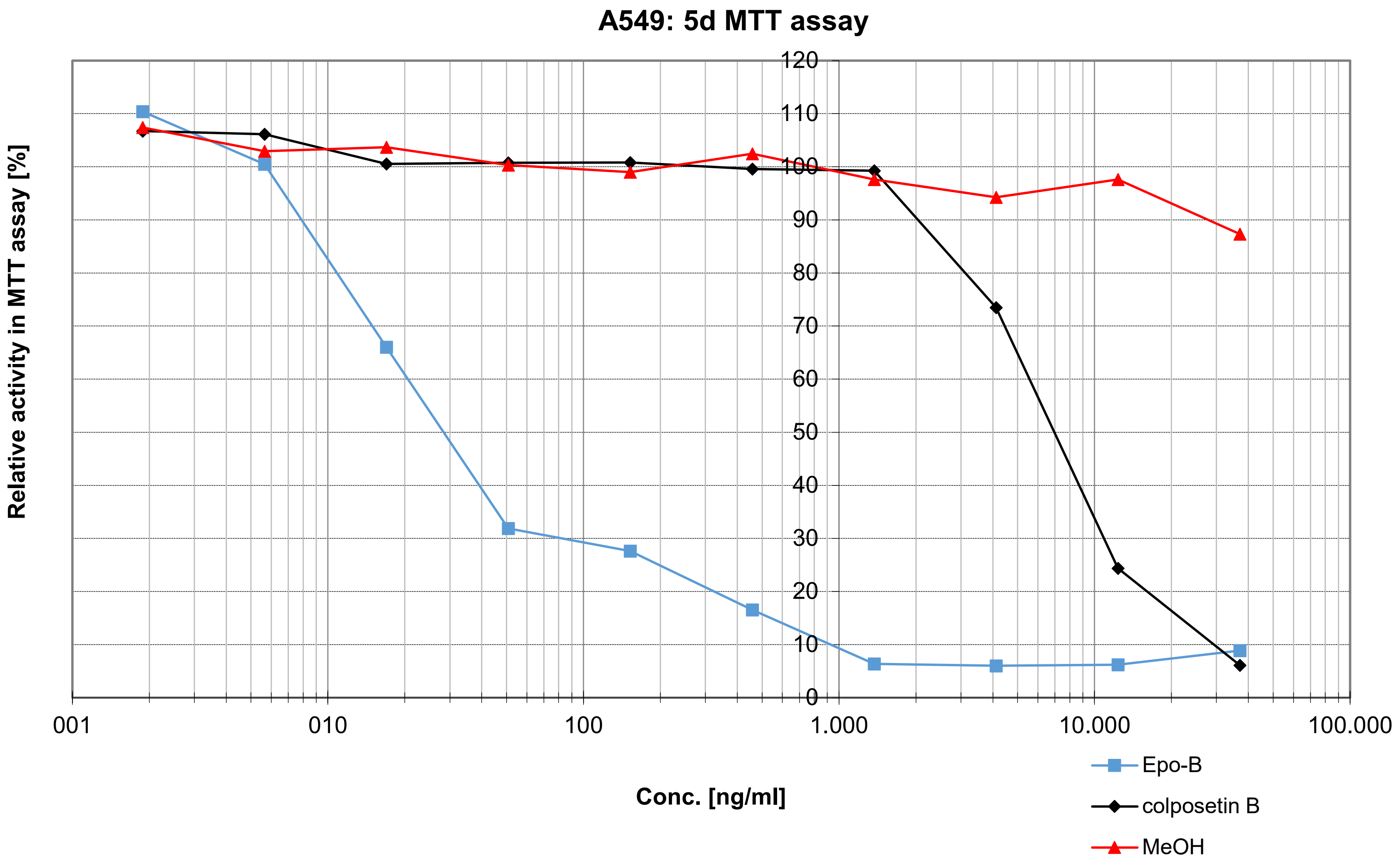

Figure S59. Graph for calculating IC_{50} of colposetin B against human lung carcinoma A-549

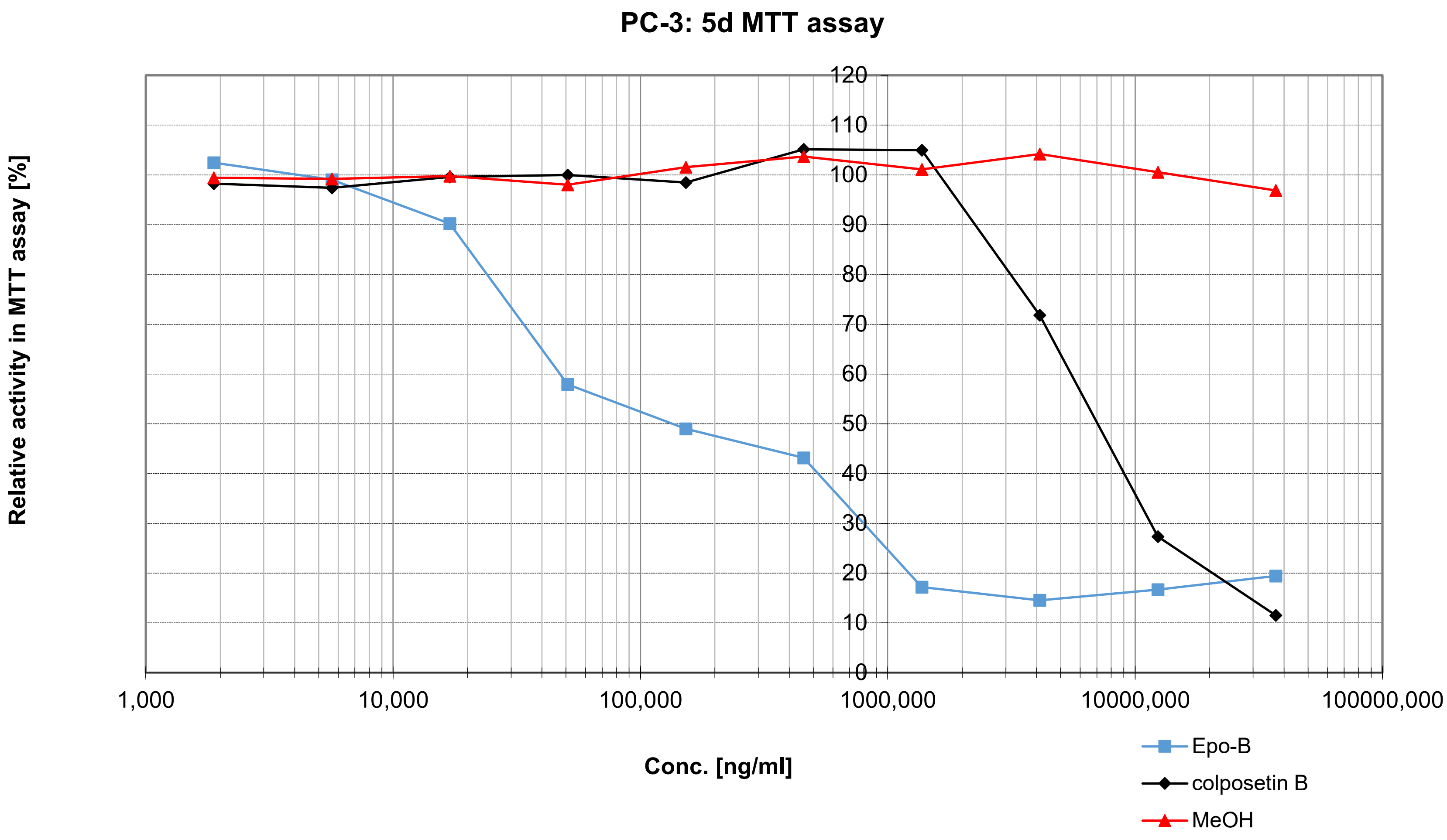

Figure S60. Graph for calculating IC_{50} of colposetin B against human prostate cancer PC-3

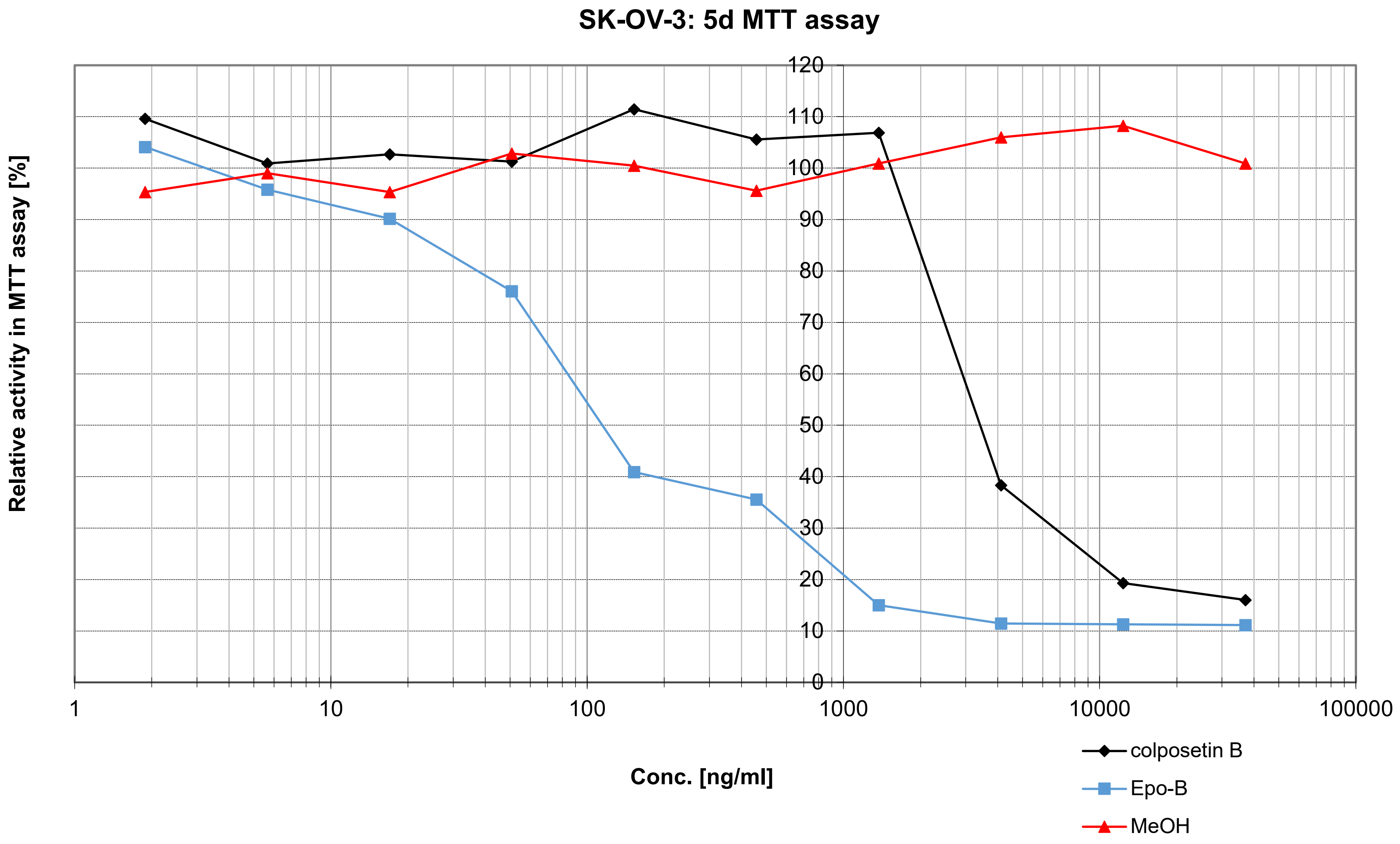

Figure S61. Graph for calculating IC_{50} of colposetin B against ovarian carcinoma SK-OV-3

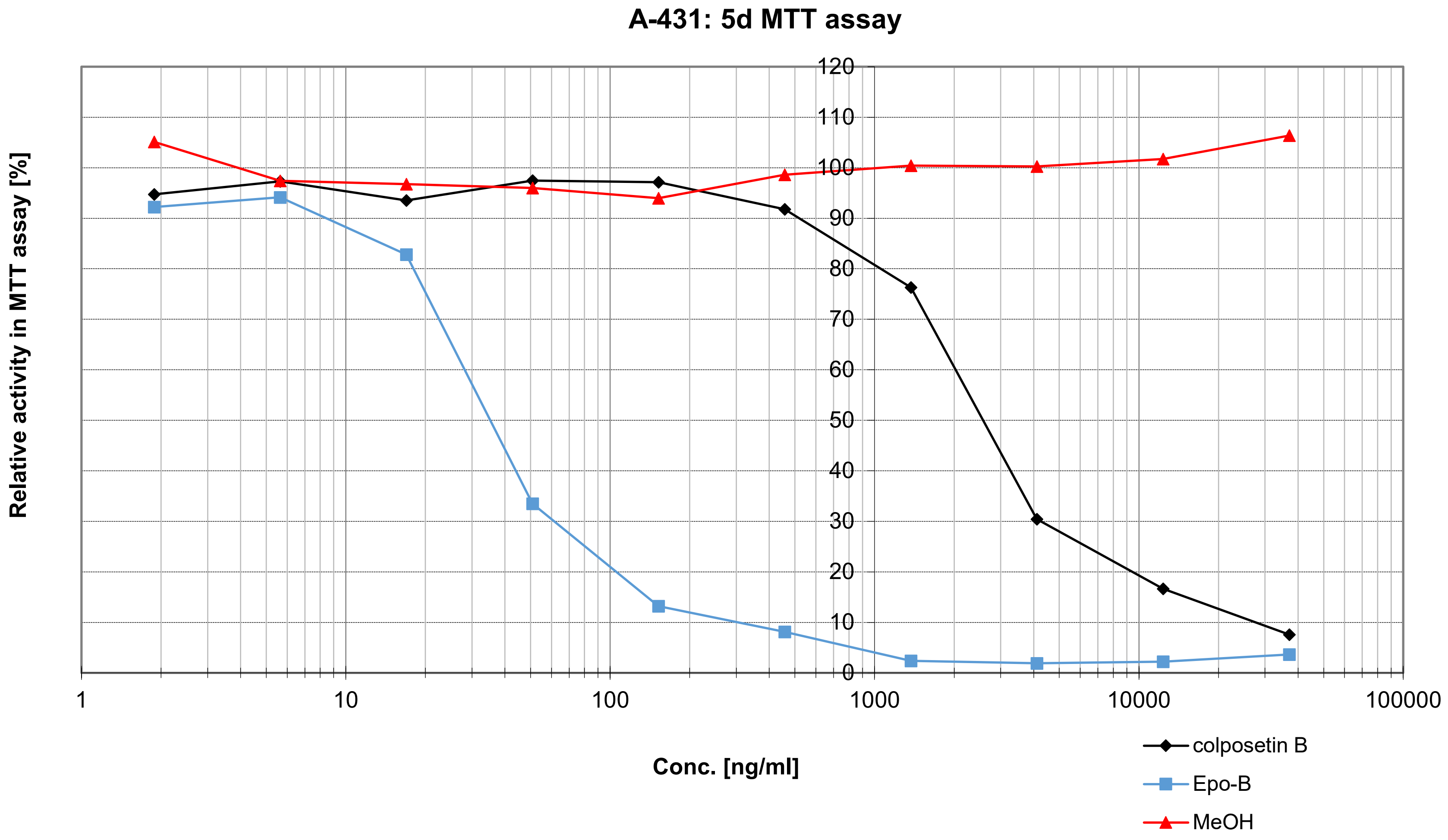

Figure S62. Graph for calculating IC_{50} of colposetin B against squamous cell carcinoma A-431

Protocol: Antimicrobial Activity Assay

The assay was conducted as a minimum inhibitory concentration (MIC) assay in 96-well round-bottom microtiter plates using the parameters summarized in Table S1 and as already described in [S1]. Stocks of the test organisms were generated by growing the organisms overnight in 50 mL shaking flasks filled with 25 mL of the growth medium at 140 rpm (media and temperatures see Table S1). If the organisms were well grown the next day, which was checked by the occurrence of an optical density (OD) >30 of the suspension (OD_{600} nm for bacteria, OD_{548} nm for fungi and *M. smegmatis*), aliquots of these were stored in 1.5 mL reaction tubes in a freezer at −80 °C for up to 12 months. Upon use, aliquots were unthawed and the OD of the suspension measured and adjusted by diluting with the respective growth medium. OD_{600} nm was adjusted to 0.01 and OD_{548} nm to 0.1. Subsequently, 150 µL of the adjusted suspensions were added to all wells of a 96-well microtiter plate (one test organism per plate). In row A, additional 130 µL of suspensions plus 20 µL of the test compounds (1 mg/mL) and the controls (one compound/column) were added. MeOH was used as negative controls, while different positive controls (references) were used for the test organisms (see Table S1). Then, starting from row A, 150 µL of the suspension were transferred to the next row, the contents thoroughly mixed, and 150 µL transferred to the following row. The remaining 150 µL after row H were discarded. This resulted in a serial dilution of the test compounds, ranging from 66.7 µg/mL in row A to 0.52 µg/mL in row H. The microtiter plates were then incubated overnight on a microplate shaker at 800 rpm at 30 or 37 °C (see Table S1) and were visually evaluated the next day. The MIC is defined as the lowest concentration where no growth of the test organism (clear zone) was observed. A lower MIC thus corresponds to a higher antimicrobial activity of the test compound.

Table S1: MIC assay experiment parameters

test organism	strain No	growth medium	incubation temp. [°C]	positive control (reference)
Bacillus subtilis	DSM10	MHB[1]	30	oxytetracyclin 1.0mg/mL
Staphylococcus aureus	DSM346	MHB[1]	30	oxytetracyclin 1.0mg/mL
Micrococcus luteus	DSM1790	MHB[1]	30	oxytetracyclin 1.0mg/mL
Chromobacterium violaceum	DSM30191	MHB[1]	30	oxytetracyclin 1.0mg/mL
Escherichia coli	DSM1116	MHB[1]	37	oxytetracyclin 1.0mg/mL
Pseudomonas aeruginosa	PA14	MHB[1]	37	gentamicin 0.1mg/mL
Mycobacterium smegmatis	ATCC700084	7H9+ADC2	37	kanamycin 0.1mg/mL
Candidaa lbicans	DSM1665	MYC[3]	30	nystatin 1.0mg/mL
Schizosaccharomyces pombe	DSM70572	MYC[3]	30	nystatin 1.0mg/mL
Mucor hiemalis	DSM2656	MYC[3]	30	nystatin 1.0mg/mL
Wickerhamomyces anomala	DSM6766	MYC[3]	30	nystatin 1.0mg/mL
Rhodotorula glutinis	DSM10134	MYC[3]	30	nystatin 1.0mg/mL

[1] MHB: Müller-Hinton Broth (SN X927.1, CarlRoth GmbH, Karlsruhe, Germany)
[2] 7H9+ADC: Middlebrook 7H9 Broth Base + Middlebrook ADC Growth Supplement (SN M0678+M0553, Merck, Darmstadt, Germany)
[3] MYC: 1 % w/v, bacto peptone, 1% w/v yeast extract, 2 % w/v glycerol, pH 6.3

[S1] Helaly, S.E.; Ashrafi, S.; Teponno, R.B.; Bernecker, S.; Dababat, A.A.; Maier, W.; Stadler, M. Nematicidal Cyclic Lipodepsipeptides and a Xanthocillin Derivative from a Phaeosphariaceous Fungus Parasitizing Eggs of the Plant Parasitic Nematode *Heterodera filipjevi*. *J. Nat. Prod*. **2018**, *81*, 2228–2234.

Protocol: Cytotoxicity Assay

The assay was conducted in 96-well flat-bottom microtiter plates using the parameters summarised in Table S2 and as described in [S2]. Cell lines L-929 and KB-3.1 were incubated at 37 °C under 10 % CO_2 in Gibco™ DMEM medium (Thermo Fisher Scientific, Waltham, MA, USA) supplemented with 10 % FBS. A microtiter plate was filled with 120 µL of this suspension (50,000/mL) in each well. Seperately, another microtiter plate was filled with 100 µL of growth medium in each well. Then, 50 µL of the test compound solutions (1 mg/mL) were given to wells of the first column in two replicates (one compound per row). Cells without additives and MeOH were used as negative controls. Starting from the first column, 50 µL of the solutions were gradually transferred to the next column, the contents thoroughly mixed, and 50 µL transferred to the following column. This created a serial dilution of the test compounds ranging from 333 µg/mL to 1.9×10^{-3} µg/mL. The remaining 50 µL after column twelve were discarded. From this microtiter plate, 60 µL of the solutions from 111 µg/mL to 1.9×10^{-3} µg/mL were given to the first plate containing 120 µL of the cell suspensions (i.e. the highest concentration 333 µg/mL was not used). This resulted in final compound concentrations ranging from 37 µg/mL to 0.6×10^{-3} µg/mL. After 5 days of incubation under the aforementioned incubation conditions, the half maximum inhibitory concentrations (IC_{50}) were determined using a colorimetric tetrazolium dye MTT assay [S3]. For this, 20 µL of a 5 mg/mL solution of 3-(4,5-dimethyl-2-thiazolyl)-2,5-diphenyl-2H-tetrazolium bromide (MTT) were added to each well and incubated for two hours at 37 °C. Then, the microtiter plate was centrifuged (3,000 rpm, 5 min) and the supernatant removed by holding the plate upside-down and gentle shaking. Afterwards, the wells were washed using 100 µL of phosphate buffered saline (PBS). The plate was again centrifuged and the supernatant removed as described before. Then, 100 µL of an isopropanol:HCl solution (1L isopropanol+4 mL HCl 37 % w/v) were added to the wells. After incubating for 10 min at ambient temperature, the absorption of the wells at 595 nm was measured with an Infinite® 200 Pro microplate reader (TECAN, Männedorf, Schweiz). The absorption values of the cells without additives were averaged and set to 100 % cell viability. Then, the means of absorption of the two compound replicates were set in relation to the blank media. These percentage values were plotted against the concentration range (37 µg/mL to $0.6\times10-3$ µg/mL). The IC_{50} value was read from the plot (in µg/mL) and the units conversed to µM. If effects were observed with cell lines L-929 and KB-3.1 ($IC_{50} < 50$ µM), the other cell lines were tested using the same protocol.

Table S1: MIC assay experiment parameters

cell line	type	No.	growth medium
L-929	mouse fibroblasts	ACC 2	DMEM[1] + 10 % FBS[2]
KB-3.1	human endocervical adenocarcinoma (AC)	ACC 158	DMEM[1] + 10 % FBS[2]
PC-3	human prostate AC	ACC 465	F-12K Nutmix[3] + 10 % FBS[2]
SK-OV-3	human ovary AC	n/a	McCoys 5a[4] + 10 % FBS[2]
MCF-7	human breast AC	ACC 115	RPMI 1640[5] + 10 % FBS[2]<br>+ 1 % MEMNEAA[6]<br>+ 1.25 mL/500 mL insulin[7]
A-431	human squamous AC	ACC 91	RPMI 16404 + 10 % FBS[2]
A-549	human lung carcinoma	ACC 107	DMEM[1]

[1] DMEM: Dulbecco's Modified Eagle Medium (SN 61965026, Thermo Fisher Scientific, Waltham, MA, USA)
[2] FBS: Fetal Bovine Serum (SN 10500064, Thermo Fisher Scientific)
[3] F-12K Nutmix: Ham's F-12K (Kaign's) Medium (SN 21127022, Thermo Fisher Scientific)
[4] McCoys 5a: McCoy's 5a (modified) Medium (SN 26600023, Thermo Fisher Scientific)
[5] RPMI 1640: RPMI 1640 Medium (SN 21875091, Thermo Fisher Scientific)
[6] MEMNEAA: MEM Non-Essential Amino Acids Solution 100× (SN 11140035, Thermo Fisher Scientific)
[7] Insulin: Human Recombinant Insulin, Zinc Solution (SN 12585014, Thermo Fisher Scientific)

[S2] Sandargo, B.; Michehl, M.; Praditya, D.; Steinmann, E.; Stadler, M.; Surup, F. Antiviral Meroterpenoid Rhodatin and Sesquiterpenoids Rhodocoranes A-E from the Wrinkled Peach Mushroom, *Rhodotus palmatus*. *Org. Lett.* **2019**, *21*, 3286–3289.

[S3] Mosmann, T. Rapid colorimetric assay for cellular growth and survival: Application to proliferation and cytotoxicity assays. *J. Immunol. Methods* **1983**, *65*, 55–63
